# Acetylenic Fatty Acids and Stilbene Glycosides Isolated from *Santalum yasi* Collected from the Fiji Islands

**DOI:** 10.3390/molecules30244752

**Published:** 2025-12-12

**Authors:** Khalid Al Maqbali, Miriama Vuiyasawa, Mercy Ayinya Gube-Ibrahim, Shubham Sewariya, Clément Balat, Kirsti Helland, Tamar Garcia-Sorribes, Mercedes de la Cruz, Bastien Cautain, Jeanette Hammer Andersen, Fernando Reyes, Jioji N. Tabudravu

**Affiliations:** 1 School of Pharmacy and Biomedical Sciences, University of Lancashire, Preston PR1 2HE, UK; khalid85@squ.edu.om (K.A.M.); shubhamsewariya94@gmail.com (S.S.); clement.balat@viacesi.fr (C.B.); tgarcia-sorribes@uclan.ac.uk (T.G.-S.); 2 Chemistry Department, College of Science, Sultan Qaboos University, Al Khoudh, Muscat 123, Oman; 3 Institute of Applied Sciences, University of the South Pacific, Suva, Private Mail Bag, Fiji; miriamavuiyasawa@gmail.com; 4 Marine Biodiscovery Centre, Department of Chemistry, University of Aberdeen, Meston Walk, Aberdeen AB24 3UE, UK; m.gube-ibrahim.22@abdn.ac.uk; 5 Department of Chemistry, College of Education Akwanga, Akwanga 960101, Nasarawa State, Nigeria; 6 Institute of Nano Medical Sciences, University of Delhi, New Delhi 110007, India; 7 Centre des Etudes Supérieures Industrielles, CESI Engineering School, Pôle Aéropolis-1 Cours de l’Industrie, 64510 Assat, France; 8 Marine Biotechnology and Biological Chemistry (Marbio), UiT the Arctic University of Norway, Breivika, 9019 Tromsø, Norway; kirsti.helland@uit.no (K.H.); jeanette.andersen@uit.no (J.H.A.); 9 Fundacion MEDINA, Centro de Excelencia en Investigacion de Medicamentos Innovadores en Andalucía, Avenida del Conocimiento 34, Parque Tecnologico de Ciencias de la Salud, 18016 Armilla, Granada, Spain; mercedes.delacruz@medinaandalucia.es (M.d.l.C.); cautainbastien@gmail.com (B.C.); fernando.reyes@medinaandalucia.es (F.R.)

**Keywords:** *Santalum yasi*, *Santalum album*, *Santalum yasi–album* hybrid, acetylenic fatty acids stilbene, cytotoxic

## Abstract

In our continuing search for new anticancer and/or antimicrobial compounds from natural products, we screened for these activities in bark and leaf extracts of sandalwood plants collected from the Fiji Islands and found *Santalum yasi* to be the most active. Resulting chemical workup enabled the isolation and structural characterization of a new acetylenic acid, methyl (*E*)-octadec-6-en-8-ynoate (**1**), and an atropisomeric stilbene glycoside (**4**) (Yasibeneoside) together with six known compounds: 11,13-octadecadien-9-ynoic acid (**2**), methyl octadeca-9,11-diynoate (**3**), gaylussacin (**5**) chrysin-7-beta-monoglucoside (**6**), neoschaftoside (**7**), and chrysin-6-C-glucoside-8-C-arabinoside (**8**). Compound **1** (18:2 (6t, 8a) is an example of a Δ^6^, Δ^8^ acetylenic system containing the *trans* double bond at C-6 and the triple bond at C-8, which is reported here for the first time. All molecular structure elucidations and dereplications were performed using spectroscopic techniques, including 2D NMR and HRMS-MS/MS spectrometry. Methyl (*E*)-octadec-6-en-8-ynoate showed moderate activity activity with an IC_50_ of 91.2 ug/mL against the human breast adenocarcinoma cell line MCF-7.

## 1. Introduction

Sandalwood represents a group of important medicinal and commercial plants belonging to the family Santalaceae, and the genus *Santalum* [1,2]. There are about 19 species of sandalwood known [3], of which one, *S. yasi*, is endemic to Fiji [4,5] and Tonga [6]. A successful genetic hybrid between *S. yasi* and *S. album* (native to India) [7] is also growing in Fiji [8]. *Santalum yasi*, like other sandalwoods, is a hemi-parasitic plant with a very slow growth rate (about 0.3–0.7 m per year) with mature trees reaching heights of about 10 metres [4]. *S. yasi* has traditionally been used in Fiji for medicine, incense, and in wedding ceremonies [9]. Commercially, *S. yasi* has often been regarded to be of high value due to its high content in α- and β-santalols that typically meet the East Indian Sandalwood ISO standard for sandalwood oil [5]. Most chemical investigative studies have been carried out on the essential oil content of sandalwood [10,11,12,13,14], but studies on other compounds [14] in sandalwood, such as *S. yasi*, are lacking. While screening for cytotoxicity and antimicrobial activities in sandalwood plants from Fiji, significant activities were observed in the bark fractions of *S. yasi*, and in the *Santalum yasi–album* hybrid species against methicillin-sensitive *Staphylococcus aureus* (MSSA ATCC-29213) and *Candida albicans* (*C. albicans* ATTCC-64124), and the cancer cell lines A549 (human lung carcinoma), A2058 (human Caucasian metastatic melanoma), HepG2 (human liver carcinoma cells), MCF-7 (human breast adenocarcinoma), MIA Paca-2 (human Caucasian pancreatic carcinoma), and PC-3 (human Caucasian prostate adenocarcinoma). Cytotoxicity activities were also observed in *S. album* leaf fractions against the cancer cell lines A2058, HepG2, and MCF-7. This report describes the biological activities of fractions of the three sandalwood species in Fiji and the structure elucidation of compounds isolated from *S. yasi*.

## 2. Results and Discussion

Bark and leaves of the three sandalwood plants were collected in Fiji and extracted with methanol (MeOH), followed by dichloromethane (DCM), to produce six crude extracts. Each crude extract was then processed using a modified Kupchan [15] liquid–liquid partitioning method to produce four fractions based on polarity: water–butanol (WB), water–methanol (FM), dichloromethane (FD), and hexane (FH). All fractions were screened for bioactivity against five clinically important human microbial pathogens (Table S1) and against six cancer cell lines (Table S2), with both *S. yasi* and the *S. yasi–album* hybrid bark fractions showing the most promising results (Figure 4). The *S. yasi* bark hexane fraction (SyB-FH) that displayed good activity against MSSA and against four of the cancer cell lines was purified further on a Sephadex LH-20 [16] column using MeOH-DCM (1:1), followed by reversed-phase purification on a C_18_ HPLC column using a H_2_O-CH_3_CN (40–60) solvent isocratic system to afford the new compound methyl (*E*)-octadec-6-en-8-ynoate (**1**), and the known compounds 11,13-octadecadien-9-ynoic acid (**2**) and methyl octadeca-9,11-diynoate (**3**).

The dichloromethane fraction of *S. yasi* leaf (SyL-FD) was fractionated on a C_18_ solid phase extraction column (SPE) using 50% H_2_O-MeOH and further purified on a C_18_ HPLC column using H_2_O-MeOH gradient system to afford 2.8 mg of yasibeneoside (4), and the known compounds gaylussacin (**5**) chrysin-7-beta-monoglucoside (**6**), neoschaftoside (**7**), and chrysin-6-C-glucoside-8-C-arabinoside (**8**).

### 2.1. Structure Elucidation

**Figure 1:**
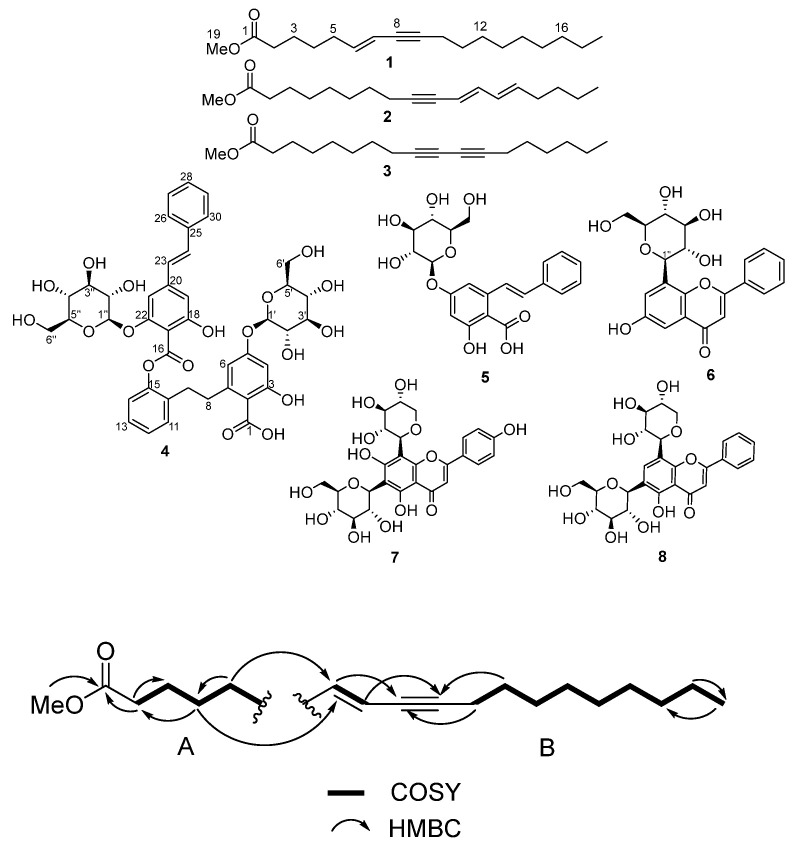
Substructures A and B showing key COSY and HMBC correlations establishing positions of unsaturation in the structure of **1**.

**Figure 2:**
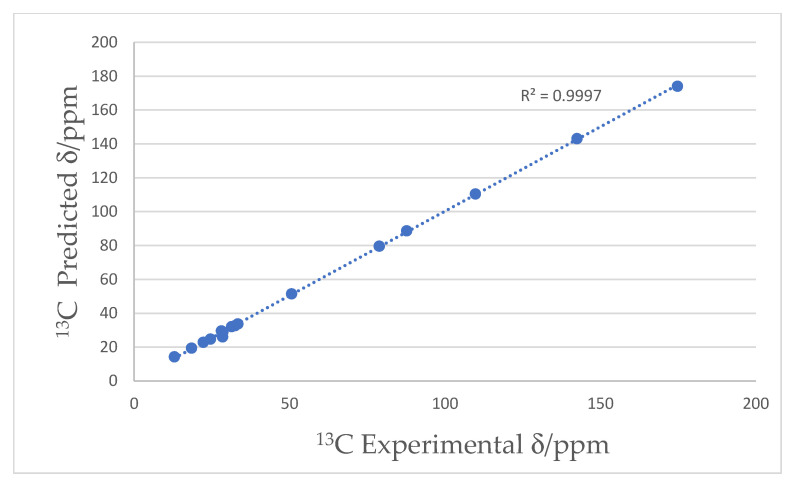
Plot of ^13^C experimental vs. predicted chemical shift data for **1**.

Compound **1** showed a HRESIMS ion at *m*/*z* 293.2475 [M + H]^+^ (Δ1.0 ppm) (Figure S11) for the expected molecular formula of C_19_H_32_O_2_ with four indices of hydrogen deficiency [17,18]. Interpretation of ^13^C, HSQC, and HMBC NMR data (Figures S1–S10, Table 1) of **1** showed the presence of 19 carbons in the form of two methyl groups (δ_C_, 50.7, 13.0), 12 sp^3^ methylenes (δ_C_ 33.4, 32.5, 31.4, 28.7, 28.6, 28.5, 28.4, 28.3, 26.1, 24.6, 22.3, 18.5), two sp^2^ methines (δ_C_ 142.5, 109.8), two non-protonated sp. carbons (δ_C_ 87.7, 78.9), and one ester carbonyl (δ_C_ 174.8). Two sp^2^ plus two sp carbons, plus one ester carbonyl, accounted for the four indices of deficiency, indicating that the structure of **1** was linear. Interpretation of 1D and 2D NMR (Figures S1–S10) data enabled the construction of two substructures (Figure 1). The position of the ester group was established by HMBC correlations of the methoxy proton at δ_H_ 3.60 (H-19) and methylenes at δ_H_ 2.27 (H-2), 1.56 (H-3) to the ester carbon at δ_C_ 174.8 (Table 1). Key COSY correlations between the methine proton at δ_H_ 5.92 (H-6) and δ_H_ 5.38 (H-7) to the methylene proton δ_H_ 2.02 (H-5) (Figures S4 and S5), HMBC correlations between the methine proton δ_H_ 5.92 (H-6) to the carbon at δ_C_ 32.5 (C-5) and the methylene protons at δ_H_ 2.27 (H-2), 1.56 (H-3), 2.02 (H-5) to the carbon at δ_C_ 28.6 (C-4) (Figures S7–S9) unambiguously established the position of the alkene unsaturation at C-6/C-7. HMBC correlations between δ_H_ 5.38 (H-7) to the carbon at δ_C_ 87.7 (C-9) and between δ_H_ 5.92 (H-6) to δ_C_ 87.7 (C-8) (Figure S10, Table 1) established the ‘ene-yne’ spin system. The *E* geometry of the double bond in the molecule was secured through a ^1^H-^1^H coupling constant of 15.7 Hz between H-6 and H-7. Further evidence for the proposed structure came from NMR chemical shift predictions. A plot of ^13^C experimental data against the predicted data calculated by ACD/Labs Structure Elucidator [19] using the HOSE [20] code is shown in Figure 2 with a linear regression of R^2^ = 0.9997, suggesting that the proposed structure is most likely correct [21,22]. Additional evidence for the structure of **1** was provided by HR-MS/MS fragmentation data, which has been annotated with fragments calculated by the ACD/labs MS Fragmenter (Version 19.2.0) [23] software (Figure S12).

**Figure 3:**
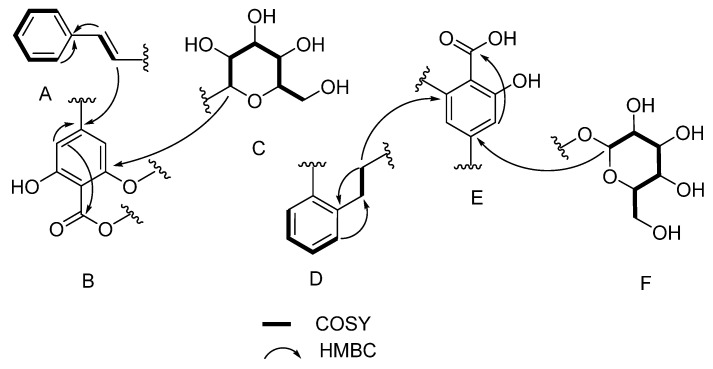
Substructures A–F derived from COSY (bold line) and HMBC correlations (H to C) for **4**.

Compound **4** showed a HRESIMS ion at *m*/*z* 837.2587 [M + H]^+^ calculated for C_42_H_45_O_18_ (837.2606, Δ = 1.6 ppm) with 21 indices of hydrogen deficiency (Figure S27). Interpretation of Edited-HSQC and HMBC NMR data (Figures S17 and S18, Table 2) indicated the presence of 42 carbons, including 15 sp^2^ methines (δ_C_ 130.8, 129.0, 129.0, 128.9, 128.1, 127.4, 127.3, 126.3, 126.3, 125.4, 125.4, 111.0, 107.4, 102.9, 101.8), 10 sp^3^ methines (δ_C_ 100.1, 99.9, 77.0, 76.9, 76.8, 76.5, 73.4, 69.9, 69.8), 4 sp^3^ methylenes (δ_C_ 61.1, 61.0, 38.5, 37.9), and 13 sp^2^ quaternary carbons (δ_C_ 172.9, 172.7, 164.7, 164.0, 161.6, 161.5, 151.5, 147.1, 142.8, 142.0, 137.6, 106.4, 106.2). The four benzene ring systems, plus one alkene, plus one ester linkage, plus one carboxylic acid group, fully accounted for the 21 indices of hydrogen deficiency in the structure of **4**. Extensive interpretation of one- and two-dimensional NMR data (Figures S15–S18) enabled the construction of six substructures (Figure 3).

The six substructures or spin systems include one mono-substituted benzene ring coupled to an alkene system (substructure A), two 1,2,3,5-tetra-substituted benzene rings (substructures B and E), one 1,2-di-substitued benzene ring linked to an ethyl moiety (substructure D), and two glycosidic spin systems (substructures C and F). Long-range HMBC correlations were used to connect these substructures to attain the full structure of **4**. HMBC correlations were observed between the proton signal at δ_H_ 6.91 (H-23) to the sp^2^ carbon at δ_C_ 142.8 (C-20), linking substructures A to B. Similarly, HMBC correlations were observed between the diastereotopic protons at δ_H_ 3.20/3.17 (H-8) to the sp^2^ quaternary carbon at δ_C_ 147.1 (C-7), linking substructures D and E. Despite the absence of HMBC correlations between substructures B and D, substructure B was most likely linked to substructure D at C-15 based on the chemical shift in this carbon (δ_C_ 151.5), suggesting that C-15 was linked to C-16 via an ester linkage. An HMBC correlation between the anomeric proton at δ_H_ 5.05 (H-1″) and the sp^2^ quaternary carbon at 161.6 ppm (C-22) linked one of the glycosides (substructure C) to substructure B. The second glycoside (substructure F) was linked to substructure E through an observed HMBC correlation between the anomeric proton at δ_H_ 4.89 (H-1′) to the sp^2^ quaternary carbon at δ_C_ 161.5 (C-5) to complete the planar structure for **4**. Additional proof for the structure of **4** was obtained by plotting predicted and experimental ^13^C NMR data (Figure S26). A strong correlation (R^2^ = 0.9996) was obtained, suggesting that the proposed planar structure for **4** is most likely correct [21,24]. Further evidence for the structure of **4** was provided by the analysis of HRMS/MS fragmentation data (Figure S28).

The geometry of the alkene system at C-23/C-24 was determined to be *E* based on the large coupling constant of 16.0 Hz between H-23 and H-24. The relative configurations of the two glycoside units were determined to be *β* based on the coupling constant of 7.4 Hz for H-1′ and H-2′, and 6.9 Hz for H-1″ and H-2″. Further evidence was shown by NOE correlations between H-1′ and H-3′/H-4′; H-2″ and H-3″/H-4″ (Figures S19–S22). NMR data show that the protons of the CH_2_ group at C-8, as well as C-9, resonate at different chemical shifts (Figures S15–S19), suggesting chirality associated with atropisomers [25,26]. The possible room temperature conformation of the structure of **4** is supported by key NOE correlations between H-8A/B and H-9A/B to H-11 and H-6 (Figures S19, S23 and S24). Figure S25 shows the minimized energy structure calculated using Chem3D Ultra (Version 16.0.0.82) [27]. The structure shows key H-bonding between the carbonyl group C-1 (carboxylic acid) and the hydroxy group at C-4″, hindering free rotation between C-8 and C-9, resulting in chemical and magnetic non-equivalence of the methylene protons at C-8/C-9 [28,29,30].

Compound **2** showed an *m*/*z* of 291.2318 [M + H]^+^ calculated for C_19_H_31_O_2_ (291.2319, Δ = −0.2 ppm) (Figure S40). Interpretation of ^1^H, 2D NMR, and HR-MS/MS data (Figures S31–S39, S41 and S42) identified 2 as 11,13-octadecadien-9-ynoic acid [31].

Compound **3** showed an *m*/*z* of 291.2319 [M + H]^+^ calculated for C_19_H_31_O_2_ (291.2319, Δ = 0.4 ppm) (Figure S48). Interpretation of ^1^H, 2D NMR, and HR-MS/MS data (Figures S43–S47 and S49) identified compound **3** as methyl octadeca-9,11-diynoate [32].

Compound **5** showed an *m*/*z* of 419.1347 [M + H]^+^ calculated for C_21_H_23_O_9_ (419.1342, Δ = −2.5 ppm) (Figure S54). Interpretation of ^1^H and 2D NMR data (Figures S50–S53) identified compound **5** as gaylussacin [32,33].

Compound **6** showed an *m*/*z* of 417.1191 [M + H]^+^ calculated for C_21_H_21_O_9_ (417.1186, Δ = −2.6 ppm) (Figure S59). Interpretation of ^1^H, ^13^C, and 2D NMR data (Figures S55–S58) identified the compound as chrysin-7-beta-monoglucoside [34,35].

Compound **7** showed an *m*/*z* of 565.1570 [M + H]^+^ calculated for C_26_H_29_O_14_ (565.1557, Δ = 2.9 ppm) (Figure S64). Interpretation of ^1^H and 2D NMR data (Figures S60–S63) identified the compound as neoschaftoside [36,37].

Compound **8** showed an *m*/*z* of 549.1618 [M + H]^+^ calculated for C_26_H_29_O_13_ (549.1608, Δ = 2.9 ppm) (Figure S69). Interpretation of ^1^H and 2D NMR data (Figures S65–S68) identified the compounds as chrysin-6-C-glucoside-8-C-arabinoside [38].

### 2.2. Biological Activity

**Figure 4:**
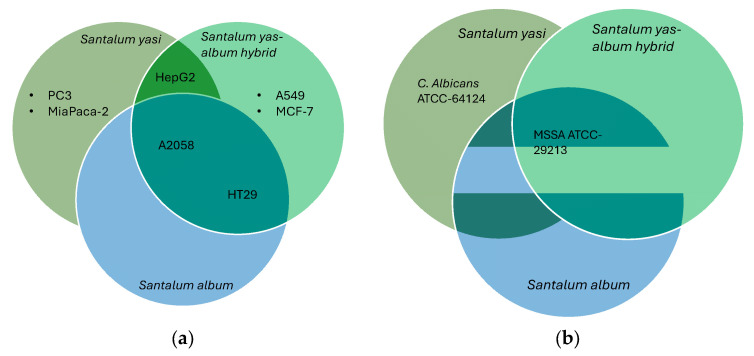
(**a**) Cytotoxic activity of the bark hexane fractions of *S. yasi*, *S. album*, and *S. yasi–album* hybrid. A549 (human lung carcinoma), A2058 (human Caucasian metastatic melanoma), HepG2 (human liver carcinoma cells), MCF7 (human breast adenocarcinoma), MiaPaca-2 (human Caucasian pancreatic carcinoma), PC-3 (human Caucasian prostate adenocarcinoma), and HT-29 (human colorectal adenocarcinoma). Both *S. yasi* and *S. yasi–album* hybrid were tested at 0.10 mg/mL, while *S. album* was tested at 0.05 mg/mL (Table S2). (**b**) Antimicrobial assay: MSSA ATCC-29213 (Methicillin-susceptible *Staphyloccocus aureus* subspecies *aureus* strain Wichita), *C. albicans* ATCC-64124 (*Candida albicans*). The *C. albicans* activity (>50% inhibition, Table S1) was reported only in the WB fraction, while the MSSA activity was recorded in the bark FH fractions of the three sandalwood plants. Antimicrobial assays of fractions were performed at 0.06 mg/mL.

The modified Kupchan fractions of leaf and bark of *S. yasi*, *S. album*, and *S. yasi–album* hybrid were screened for antibacterial, antifungal, and cytotoxic activities. Leaf fractions of the three sandalwood plants displayed low to mild activity at 0.32 mg/mL against the pathogens tested (Table S1), with the butanol (WB) fraction of the bark of *S. yasi* showing good activity against *Candida albicans*. The strongest activities were shown by the FH fractions of the bark of *S. yasi* and *S. yasi–album* hybrid against MSSA ATCC-29213 (>90% inhibition) (Figure 4b, Table S1).

For the cytotoxicity assay, both *S. yasi bark* (FH) and *S. album* leaf (FM) showed activity (60–90%) at 0.10 mg/mL against human Caucasian metastatic melanoma (A2058), human liver carcinoma cells (HepG2), human breast (adenocarcinoma) (MCF7), human Caucasian pancreatic carcinoma (MIA PaCa-2), and human Caucasian prostate adenocarcinoma (PC-3), with the strongest activity shown by the *S. yasi* bark fraction (FH) at 90% inhibition of HepG2 cells. *S. yasi–album* hybrid bark (FH) inhibited both human lung carcinoma (A549) and human colorectal adenocarcinoma (HT-29), in addition to the cell lines inhibited by *S. yasi and S. album* (Figure 4a, Table S2).

Methyl (*E*)-octadec-6-en-8-ynoate (**1**) showed an IC_50_ of 91.2 ug/mL (Table S3 against MCF7, but showed no activity against A2058 and human fibroblast cells (MRC-5). Compounds **5**–**8** showed weak cytotoxic activity.

## 3. Materials and Methods

### 3.1. Reagents and Solvents

All solvents used for chromatography purifications were HPLC grade, while LCMS solvents were MS grade. Both were obtained from Fisher Scientific (West Sussex, UK) [39], and NMR solvents were obtained from Goss Scientific (Crew, UK) [40].

### 3.2. Main Instruments

UV spectra were recorded on an Agilent Technologies (Stockport, UK) 1220 Infinity Photodiode array detector [41]. IR spectra were recorded on a Shimadzu (Milton Keynes, UK) Fourier transform infrared spectrophotometer (IRTracer-100) [42]. NMR spectroscopic data for compounds **4**–**8** were recorded at the University of Edinburgh [43] at 25 °C on a Bruker (Coventry, UK) Avance NEO 800 MHz with a He-cooled cryoprobe. Compounds **1**–**3** were recorded at the Biodiscovery Centre, University of Aberdeen [44], on a Bruker AVANCE III HD Prodigy TCI cryoprobe at 600 and 150 MHz for ^1^H and ^13^C, respectively. This instrument was optimized for ^1^H observation with pulsing/decoupling of ^13^C and ^15^N with ^2^H lock channels equipped with shielded z-gradients and cooled preamplifiers for ^1^H and ^13^C. The ^1^H and ^13^C chemical shifts were referenced to the solvent signals (δ_H_ 3.31 and δ_C_ 49.00 in CD_3_OD). LC-HRESIMS analysis for compounds **1**–**4** was performed on an Agilent (Stockport, UK) 1290 LC system with a photodiode array detector (DAD) coupled to an Agilent (Stockport, UK) 6546 LC-QTOF [41], equipped with dual-spray jet stream technology electrospray ion source (AJS) [45]. The system was controlled by the Mass Hunter 11.0 [46] software. Liquid chromatographic separations were performed at 40 °C on a Phenomenex (Maccelesfield, UK) Kinetex phenyl hexyl 100 × 3.0 mm, 1.7 μm [47] equipped with a security guard column. A linear CH_3_CN-H_2_O gradient of 20% CH_3_CN–water to 100% CH_3_CN in 12 min was applied at a constant flow rate of 0.4 mL/min; then, 100% CH_3_CN was maintained for 3 min before returning to the starting conditions in 1 min and equilibrating for a further 3 min. Formic acid (0.1% *v*/*v*) was added to all solvents, and UV spectra were collected by a DAD from 200 to 500 nm with a resolution of 2 nm. Tuning in ESI^+^ mode was performed using Agilent’s tuning mix [48] of six masses to a resolution of FWHM between 40,495 for [M + H]^+^ of 118.086255 at the lower end and 64,549 for 1521.971475 at the upper end, thereby giving rise to an average accuracy of <1.0 ppm within the mass range. Mass accuracy was maintained throughout the sample analysis via the use of dual-spray technology using Agilent′s reference solution mix [48]. Scanning source parameters for ESI^+^ were as follows: capillary (3500 V), nozzle (1000 V), fragmentor (190 V), skimmer1 (65 V), and octapole RF peak (750 V). Targeted MS^2^ (ES^+^) fragmentation mode for compound **1** (*m*/*z* 293.2501) was performed at four collision energies: 10, 20, 30, and 40 V at peak retention time (t_R_) of 9.80 ± 0.06 min. Targeted MS^2^ (ES^−^) fragmentation mode for compound **4** (*m*/*z* 835.2455) was performed at four collision energies: 5, 10, 15, and 30 V at peak retention time (t_R_) of 3.08 ± 0.06 min.

LC-HRESIMS for compounds **5**–**8** were performed on a Bruker Maxis II (Bremen, Germany) Time of Flight instrument [44,49] using the following parameters: capillary voltage 45 V, capillary temperature 320 °C, auxiliary gas flow rate 10–20 arbitrary units, sheath gas flow rate 40–50 arbitrary units, spray voltage 4.5 kV, mass range 100–2000 amu, and resolution 80,000 for HRESIMS.

### 3.3. Chromatography

Sephadex LH-20 [50] was sourced from Merk (Felthem, UK) [16]. Solid phase extractions were performed using Phenomenex (Maccelesfield, UK) C18 cartridges (Strata C18-E, 55 um, 70 Å) [51]. Semipreparative HPLC purifications were performed on an Agilent (Stockport, UK) 75 1100 HPLC system consisting of a binary pump, degasser, and photodiode array detector.

### 3.4. Plant Collection and Extraction

Sandalwood leaves and bark were collected from the Fiji Islands: *S. yasi* from 114 Milverton road, Suva (−18.1349, 178.4476) [52], *S. album* from Vavalagi road, Nakasi (−18.06517, 178.5154) [52], and *S. yasi–album* hybrid from Yauvula, Wainunu, Bua (−16.805842, 178.889114) [52]. All samples were taken to the Institute of Applied Sciences, University of the South Pacific [53], Suva, for processing. All plants were taxonomically identified based on morphological features at the South Pacific Regional Herbarium [54], where voucher specimens are kept with the following collection numbers: *S. yasi* (Tuiwawa5237), *S. album* (Tuiwawa5238), and *S. yasi–album* hybrid (Tuiwawa5239). Bark (10 g) and leaves (5 g) were extracted separately with methanol (3×), followed by dichloromethane (3×). Extracts were dried under vacuum and fractionated using the modified Kupchan liquid–liquid partitioning technique [15,55] to produce four fractions: hexane (FH), dichloromethane (FD), methanol–water (FM), and butanol–water (WB). These four fractions were dried and shipped to the University of Central Lancashire (UCLan) in the United Kingdom.

### 3.5. Purification and Isolation

The FH fraction of the bark of *S. yasi* was subjected to size exclusion chromatography using the Sephadex LH-20 gel (Merck, Felthem, UK) as the stationary phase, eluted with dichloromethane-MeOH (1:1) to yield 10 Sephadex fractions. The most interesting fraction in terms of ^1^H NMR profile was fraction Sephadex-4 (S-4), which was subjected to HPLC purification on a Waters (Wilmslow, UK) Sunfire C18 OBD^TM^ semi-Prep column (250 × 10 mm) using a solvent gradient from 0 to 100% CH_3_CN in 25 min and maintained at 100% CH_3_CN for a further 10 min with a flow rate of 1.5 mL/min to yield 2.5 mg of **1**, 2.3 mg of **2**, and 1.8 mg of **3**.

The FD fraction of the leaf extract of *S. yasi* was fractionated further into four sub-fractions using reversed-phase C-18 SPE cartridges, where, after conditioning of the column as recommended by Phenomenex [51], the column was flushed with 100% water to remove salts and other polar compounds, followed by 25%, 50%, and 100% (MeOH-H_2_O). After drying under nitrogen flow, fractions were profiled by ^1^H NMR with the SPE-50% fraction selected for further purification on an ACE 5 C18 HL 250 × 10 mm using an isocratic solvent system of 30% CH_3_CN-H_2_O at a flow rate of 1.5 mL/min to yield 3.0 mg of **4**, 1.9 mg of **5**, 1.8 mg of **6**, 1.7 mg of **7**, and 1.6 mg of **8**.

### 3.6. Cytotoxicity Assays

Cytotoxicity assays for compound **1** were performed as follows, following the method described by Schlüter et al. [56]: Human melanoma cells (A2058, ATCC no: CRL-1147) were grown and assayed in Dulbecco’s Modified Eagle’s Medium (DMEM, Sigma D6171) (Sigma, Madrid, Spain) supplemented with fetal bovine serum (FBS), glutamine stable and gentamycin. Human breast adenocarcinoma (MCF7, ATCC no HTB-22) and normal human lung fibroblasts (MRC-5, ATCC no: CCL-171) were grown and assayed in minimum essential medium eagle (MEM, Sigma M7278) supplemented with FBS, stable glutamine, non-essential amino acids, sodium pyruvate, and gentamycin. Cells were seeded in 96-well microtiter plates and incubated for 24 h at 37 °C with 5% CO_2_. After 24 h, the samples were added at a concentration of 100 µg/mL, and the cells were incubated for 72 h at 37 °C with 5% CO_2_. Cell seeding density was 2000 cells/well for A2058 and MCF7, respectively. MR-C5 cells were seeded at 4000 cells/well. Cytotoxicity assay for 1 was measured by adding CellTiter 96 Aqueous One Solution (Promega, Madrid, Spain). Metabolically active cells will reduce the yellow MTS salt to purple formazan. After 60 min of incubation with Aqueous One Solution, absorbance was read at 490 nm. The quantity of formazan measured at 490 nm is directly proportional to the number of living cells. Cell survival was then calculated by comparing the samples with a negative control (100% cell survival) and a positive control (100% cell death).

The cytotoxic activity of sandalwood fractions were tested against seven different human cancer cell lines: A549 (lung carcinoma), A2058 (metastatic melanoma), MCF7 (breast adenocarcinoma), HT-29 (colorectal adenocarcinoma MIA PaCa-2 (pancreatic carcinoma), PC-3 (prostate adenocarcinoma), and HepG2 (hepatocyte carcinoma), based on the MTT (3-(4,5-dimethylthiazol-2-yl)-2,5-diphenyltetrazolium bromide) assay [57]. Fractions were tested in duplicate following an established methodology [58].

### 3.7. Antimicrobial Assays

Antimicrobial testing against human pathogenic Gram-positive bacteria (methicillin-sensitive *Staphylococcus aureus* (MSSA) ATCC29213 and methicillin-resistant *Staphylococcus aureus* (MRSA) MB5393) and Gram-negative bacteria (*Escherichia coli* ATCC 25922, *Klebsiella pneumoniae* ATCC700603, *Pseudomonas aeruginosa* PAO-1, and *Acinetobacter baumannii* MB5973), yeast (*Candida albicans* ATCC64124), and fungi (*Aspergillus fumigatus* ATCC46645) was performed following established procedures [59]. Fractions were tested in duplicate.

## 4. Conclusions

The study has shown the potential of sandalwood plants as a source of cytotoxic compounds, in particular *S. yasi* (and *S. yasi–album* hybrid), resulting in the isolation and characterization of two new compounds, one of which is compound **1**, an acetylenic acid, showing moderate cytotoxic activity against breast cancer cells, MCF-7. Even though acetylenic acids (**1**–**3**) have been isolated for the first time from the bark of *S. yasi,* this class of compound has been previously identified in sandalwood. Ximenynic acid has been known to occur in seeds of *S. album*, *S. insulare*, and others [13,60], and the compound is known for its various biological activities, including anti-inflammatory, anticancer, larvicidal, and antimicrobial, and widely used in the cosmetic industries [61,62]. New acetylenic acids have been isolated from the bark of *Nanodea muscosa* [63], a plant that belongs to the same family as *S. yasi*. Unsaturated fatty acids with double and/or triple bonds have been of interest due to their potency against fungal pathogens [64], with undecylenic acid (UDA) being an example of such a compound that is on the market as an antifungal agent [65]. However, the position of the double and triple bond can vary [66], and bioactivity is dependent on the length of the fatty acid chain and position of unsaturation [65]. Known acetylenic acids previously isolated from *Santalum* sp. have the usual Δ^9^, Δ^12^ unsaturation carbons [66]. Compound **1** has the *trans* double bond at C-6 and a triple bond at C-8, which is different from those previously reported in the literature, where C-6 is either a *cis*-double bond conjugated to *cis* double bonds at C-9 [67] or a triple bond at C-6, either alone or in conjugation with *cis*/*trans* double [67,68] or triple bonds [65]. The closest related compound is 6-octadecen-9-ynoic acid isolated from the nuts of *Ongokea klaineana* [69]. Furthermore, alkyne bond-containing natural products, such as polyacetylenes, have been known for their strong antimicrobial activities [70,71] against drug-resistant strains such as MRSA [72,73], as well as anticancer activities [74], making them potential drug lead templates. Yasibeneoside (**4**) adds a new structure to the stilbene family of structures that have been extensively studied for their cancer preventative and tumour suppression effects [75,76,77,78,79]. Stilbene-containing scaffolds, such as tamoxifen and raloxifene, are FDA-approved synthetic drugs in clinical settings and have been known to lower the risk for breast cancer [80], underpinning the importance of this scaffold in drug discovery and development. In addition, yasibeneoside displays atropisomerism, a property that is attracting a lot of interest in the drug discovery and drug development field due to its potential effects on biological systems [26]. However, due to sample limitations in this study, bioactivity investigation for yasibeneoside was not carried out.

## Figures and Tables

**Table 1:** NMR spectroscopic data (600/150 MHz, CD_3_OD) for compound **1**.

Pos.	δ_C_, Type	δ_H_ (*J* in Hz)	COSY ^1^H−^1^H	HMBC ^1^H→^13^C
1	174.8, C			
2	33.4, CH_2_	2.27, t, 7.6	3	1, 3, 4
3	24.6, CH_2_	1.56, t, 7.4	2, 4	1, 2, 4, 6
4	28.6, CH_2_	1.28, ovlp ^a^	3,4	6
5	32.5, CH_2_	2.02, m	4, 6, 7	6, 5
6	142.5, CH	5.92, m	4,7	8
7	109.8, CH	5.38, d, 15.7	5, 6, 5	9
8	78.9, C			
9	87.7, C			
10	18.5, CH_2_	2.20, t, 7.5	11	8, 9
11	28.5, CH_2_	1.45, ovlp	10	9
12	28.4, CH_2_	1.36, ovlp	11, 13	10
13	28.3, CH_2_	1.32, ovlp	12, 14	
14	28.2, CH_2_	1.28, ovlp	13,15	
15	28.1, CH_2_	1.29, ovlp	14,16	
16	31.4, CH_2_	1.24, ovlp	15, 17	15, 17
17	22.3, CH_2_	1.26, ovlp	16,17	18
18	13.0, CH_3_	0.85, t, 7.02	17, 19	16, 17, 31
19	50.7, CH_3_	3.60, s	18	1

^a^ ovlp = overlap.

**Table 2:** NMR spectroscopic data (800/200 MHz, CD_3_OD) for compound **4**.

Pos	δ_C_, ^a^ Type	δ_H_ (*J*/Hz)	COSY ^1^H−^1^H	HMBC ^1^H→^13^C
1	172.9, C			
2	106.4, C			
3	164.7, C			
4	101.8, CH	6.50, d, 7.4		1, 3, 5
5	161.5, C			
6	111.0, CH	6.46, d, 2.3		1, 5, 7
7	147.1, C			
8	38.5, CH_2_	A: 3.20, m	9	2, 6, 7, 10
		B: 3.17, m	9	2, 7,10
9	37.9, CH_2_	A: 2.89, m	8A, 8B	7, 10
		B: 2.85, m	8A, 8B	7, 10
10	142.0, C			
11	128.2, CH	7.22, d, 8.0	12	10, 12, 15
12	125.6, CH	7.17, t, 7.7	11	13
13	128.0, CH	7.26, t, 8.0	14	12, 15
14	127.6, CH	7.17, d, 8.0	13	12, 15
15	151.5, C			
16	172.7, C			
17	106.2, C			
18	164.0 C			
19	102.9, CH	6.60, d, 2.3	21	16, 17, 18, 21, 23
20	142.8, C			
21	107.4, CH	6.92, d, 2.2	19	16, 17, 19, 22, 23
22	161.6, C			
23	128.9, CH	6.91, d, 16.0	24	19, 20
24	130.8, CH	7.98, d, 16.0	23	20, 24
25	137.6, C			
26/30	126.3, CH	7.52, d, 8.0	28	25, 27, 28
27/29	129.0, CH	6.95, d, 8.0	28	25, 28
28	128.0, CH	7.35, d, 8.0	27/29, 26/30	
1′	99.9, CH	4.74, d, 7.0	2′	2′, 5
2′	73.4 CH	3.49, m	1′, 3′	
3′	76.8, CH	3.39, m	2′, 4′	2′
4′	69.8, CH	3.48, m	3′	
5′	76.5, CH	3.44, m	4′	5′
6′	61.0, CH_2_	A: 3.91, dd, 11.8, 2.2	5′	
		B: 3.72, m	5′	
1″	100.1, CH	5.05, d, 7.0	2″	22
2″	73.4, CH	3.50, m	1″, 4″	2″
3″	76.9, CH	3.46, m	2″, 4″	
4″	69.9, CH	3.41, m	3″	2″
5″	77.0, CH	3.53, m	4″, 6″A, 6″B	
6″	61.1, CH_2_	A: 3.93, dd, 11.8, 2.2	5″	5″
		B: 3.72, m	5″	

^a^ Carbons extracted from 2D NMR (HSQC and HMBC).

## Data Availability

Data are contained within the article and Supplementary Materials.

## Supplementary Materials

The following supporting information can be downloaded at: https://www.mdpi.com/article/10.3390/molecules30244752/s1.

## References

[1] Diaz-Chavez M.L., Moniodis J., Madilao L.L., Jancsik S., Keeling C.I., Barbour E.L., Ghisalberti E.L., Plummer J.A., Jones C.G., Bohlmann J. (2013). Biosynthesis of Sandalwood Oil: Santalum Album CYP76F Cytochromes P450 Produce Santalols and Bergamotol. PLoS ONE.

[2] Boruah T., Parashar P., Ujir C., Dey S.K., Nayik G.A., Ansari M.J., Nejad A.S.M., Nayik G.A., Ansari M.J. (2023). Chapter 6—Sandalwood Essential Oil. Essential Oils.

[3] Santalum L. Plants of the World Online|Kew Science. http://powo.science.kew.org/taxon/urn:lsid:ipni.org:names:30113685-2.

[4] Parham J.W. Plants of the Fiji Islands. https://www.pemberleybooks.com/product/plants-of-the-fiji-islands/16584/.

[5] Thomson L.A.J., Bush D., Lesubula M. (2020). Participatory Value Chain Study for Yasi Sandalwood (*Santalum yasi*) in Fiji. Aust. For..

[6] CSIRO Research Publications Repository—Publication. https://publications.csiro.au/publications/publication/PIcsiro:EP204211.

[7] Solanki N.S., Chauhan C.S., Vyas B., Marothia D. (2015). Santalum Album Linn: A Review. Int. J. PharmTech Res..

[8] (PDF) Assessing Genetic Diversity of Natural and Hybrid Populations of Santalum Yasi in Fiji and Tonga. https://www.researchgate.net/publication/299425345_Assessing_genetic_diversity_of_natural_and_hybrid_populations_of_Santalum_yasi_in_Fiji_and_Tonga.

[9] ThriftBooks Secrets of Fijian Medicine Book by Michael A Weiner. https://www.thriftbooks.com/w/secrets-of-fijian-medicine_unknown/22012057/.

[10] Struthers R., Lamont B.B., Fox J.E.D., Wijesuriya S., Crossland T. (1986). Mineral Nutrition of Sandalwood (*Santalum spicatum*). J. Exp. Bot..

[11] Ochi T., Shibata H., Higuti T., Kodama K., Kusumi T., Takaishi Y. (2005). Anti- *Helicobacter p ylori* Compounds from *Santalum album*. J. Nat. Prod..

[12] Kuttan R., Nair N.G., Radhakrishnan A.N., Spande T.F., Yeh H.J., Witkop B. (1974). Isolation and Characterization of γ-L-Glutamyl-S-(Trans-1-Propenyl)-L-Cysteine Sulfoxide from Sandal (*Santalum album*). Interesting Occurrence of Sulfoxide Diastereoisomers in Nature. Biochemistry.

[13] Butaud J.-F., Raharivelomanana P., Bianchini J.-P., Gaydou E.M. (2008). Santalum Insulare Acetylenic Fatty Acid Seed Oils: Comparison within the Santalum Genus. J. Am. Oil Chem. Soc..

[14] Howes M.-J.R., Simmonds M.S.J., Kite G.C. (2004). Evaluation of the Quality of Sandalwood Essential Oils by Gas Chromatography–Mass Spectrometry. J. Chromatogr. A.

[15] Kupchan S.M., Stevens K.L., Rohlfing E.A., Sickles B.R., Sneden A.T., Miller R.W., Bryan R.F. (1978). Tumor Inhibitors. 126. New Cytotoxic Neolignans from Aniba Megaphylla Mez. J. Org. Chem..

[16] Sephadex|Sigma-Aldrich. https://www.sigmaaldrich.com/GB/en/search/sephadex?focus=products&page=1&perpage=30&sort=relevance&term=sephadex&type=product.

[17] Fred W., McLafferty Interpretation of Mass Spectra, Third Edition. University Science Books, Mill Valley, California, 1980. pp. Xvii + 303—White V—1982—Biological Mass Spectrometry—Wiley Online Library. http://onlinelibrary.wiley.com/doi/10.1002/bms.1200090610/abstract.

[18] Pellegrin V. (1983). Molecular Formulas of Organic Compounds: The Nitrogen Rule and Degree of Unsaturation. J. Chem. Educ..

[19] CASE NMR Software|Structure Elucidator Suite. https://www.acdlabs.com/products/spectrus-platform/structure-elucidator-suite/.

[20] Bremser W. (1978). Hose—A Novel Substructure Code. Anal. Chim. Acta.

[21] Elyashberg M., Williams A. (2021). ACD/Structure Elucidator: 20 Years in the History of Development. Molecules.

[22] Rateb M.E., Tabudravu J., Ebel R., Ramesh V. (2016). NMR Characterisation of Natural Products Derived from Under-Explored Microorganisms. Nuclear Magnetic Resonance.

[23] Mass Spec Fragment Prediction Software|MS FragmenterTM. ACD/Labs. http://www.acdlabs.com.

[24] Elyashberg M.E., Williams A., Blinov K. (2012). Contemporary Computer-Assisted Approaches to Molecular Structure Elucidation; New Developments in NMR.

[25] Roos C.B., Chiang C.-H., Murray L.A.M., Yang D., Schulert L., Narayan A.R.H. (2023). Stereodynamic Strategies to Induce and Enrich Chirality of Atropisomers at a Late Stage. Chem. Rev..

[26] Smyth J.E., Butler N.M., Keller P.A. (2015). A Twist of Nature—The Significance of Atropisomers in Biological Systems. Nat. Prod. Rep..

[27] PerkinElmer ChemDraw Professional. Get the Software Safely and Easily. https://perkinelmer-chemdraw-professional.software.informer.com/16.0/.

[28] Fragkiadakis M., Thomaidi M., Stergiannakos T., Chatziorfanou E., Gaidatzi M., Michailidis Barakat A., Stoumpos C., Neochoritis C.G. (2024). High Rotational Barrier Atropisomers. Chem.—A Eur. J..

[29] Lanman B.A., Parsons A.T., Zech S.G. (2022). Addressing Atropisomerism in the Development of Sotorasib, a Covalent Inhibitor of KRAS G12C: Structural, Analytical, and Synthetic Considerations. Acc. Chem. Res..

[30] Parker D., Taylor R.J., Ferguson G., Tonge A. (1986). Origins of the Proton NMR Chemical Shift Non-Equivalence in the Diastereotopic Methylene Protons of Camphanamides. Tetrahedron.

[31] PubChem Methyl (11E,13E)-Octadeca-11,13-Dien-9-Ynoate. https://pubchem.ncbi.nlm.nih.gov/compound/92040344.

[32] Methyl 9,11-Octadecadiynoate—PubChem—CompoundNCBI. https://pubchem.ncbi.nlm.nih.gov/compound/14957561.

[33] Askari A., Worthen L.R., Shimizu Y. (1972). Gaylussacin, a New Stilbene Derivative from Species of Gaylussacia. Lloydia.

[34] Chrysin-7beta-Monoglucoside—PubChem Compound—NCBI. https://www.ncbi.nlm.nih.gov/pccompound/?term=chrysin+monoglucoside.

[35] Chrysin-7beta-Monoglucoside—Chemical Compound|PlantaeDB. https://plantaedb.com/compounds/chrysin-7beta-monoglucoside.

[36] PubChem Neoschaftoside. https://pubchem.ncbi.nlm.nih.gov/compound/442619.

[37] Besson E., Chopin J., Markham K.R., Mues R., Wong H., Bouillant M.-L. (1984). Identification of Neoschaftoside as 6-C-β-d-Glucopyranosyl-8-C-β-l-Arabinopyranosylapigenin. Phytochemistry.

[38] PubChem Chrysin 6-C-Glucoside 8-C-Arabinoside. https://pubchem.ncbi.nlm.nih.gov/compound/21722007.

[39] Lab Equipment and Lab Supplies | Fisher Scientific. https://www.fishersci.co.uk/gb/en/home.html.

[40] Stable Isotopes, NMR Solvents and Tubes. https://www.ukisotope.com/.

[41] LC/MS Instruments, HPLC MS, LC/MS Systems, LC/MS Analysis|Agilent. https://www.agilent.com/en/product/liquid-chromatography-mass-spectrometry-lc-ms/lc-ms-instruments/quadrupole-time-of-flight-lc-ms.

[42] FTIR Spectroscopy. https://www.shimadzu.com/an/products/molecular-spectroscopy/ftir/index.html.

[43] University of Edinburgh—Connect NMR UK. https://www.connectnmruk.ac.uk/facility/edinburgh-university/.

[44] Marine Biodiscovery. The School of Natural and Computing Sciences. The University of Aberdeen. https://www.abdn.ac.uk/ncs/departments/chemistry/research/marine-biodiscovery/.

[45] High Sensitivity MS, High Sensitivity Ion Source, Jet Stream|Agilent. https://www.agilent.com/en/product/liquid-chromatography-mass-spectrometry-lc-ms/lc-ms-ion-sources/jet-stream-technology-ion-source-ajs.

[46] Robust Mass Spectrometry Application Software, MassHunter|Agilent. https://www.agilent.com/en/product/software-informatics/mass-spectrometry-software.

[47] Phenomenex UHPLC, HPLC, SPE, GC—Leader in Analytical Chemistry Solutions. https://www.phenomenex.com/?gclid=EAIaIQobChMIlYLs75LP_wIVw9_tCh0eAgYsEAAYASAAEgJA4PD_BwE.

[48] G1969-85000|Agilent. https://www.agilent.com/store/productDetail.jsp?catalogId=G1969-85000.

[49] Quadrupole Spectrometer—maXis II^TM^—Bruker Daltonics—TOF-MS/MS/MS/for Pharmaceutical Applications. https://www.directindustry.com/prod/bruker-daltonics/product-30029-991983.html.

[50] Moore P.M., Rodrigues A.E., LeVan M.D., Tondeur D. (1989). Gel Filtration Chromatography. Adsorption: Science and Technology.

[51] Solid Phase Extraction (SPE) Method Development Tool from Phenomenex. https://www.phenomenex.com/Tools/SPEMethodDevelopment.

[52] Google Maps. https://www.google.com/maps/place/Koronivia/@-18.0499986,178.5127337,8040m/data=!3m1!1e3!4m14!1m7!3m6!1s0x6e1be1dc6b6b9e01:0x3298dfc740562b9!2sKoronivia!8m2!3d-18.05!4d178.5333333!16s%2Fg%2F11cnyr0crw!3m5!1s0x6e1be1dc6b6b9e01:0x3298dfc740562b9!8m2!3d-18.05!4d178.5333333!16s%2Fg%2F11cnyr0crw?entry=ttu.

[53] The Institute of Applied Sciences—The Institute of Applied Sciences. https://www.usp.ac.fj/the-institute-of-applied-sciences/.

[54] South Pacific Regional Herbarium and Biodiversity Centre. https://www.usp.ac.fj/the-institute-of-applied-sciences/south-pacific-regional-herbarium-and-biodiversity-centre/.

[55] Tabudravu J.N., Jaspars M. (2001). Stelliferin Riboside, a Triterpene Monosaccharide Isolated from the Fijian Sponge *Geodia globostellifera*. J. Nat. Prod..

[56] Schlüter L., Hansen K.Ø., Isaksson J., Andersen J.H., Hansen E.H., Kalinowski J., Schneider Y.K.-H. (2024). Discovery of Thiazostatin D/E Using UPLC-HR-MS2-Based Metabolomics and σ-Factor Engineering of *Actinoplanes* sp. SE50/110. Front. Bioeng. Biotechnol..

[57] Basic Colorimetric Proliferation Assays: MTT, WST, and Resazurin. Springer Nature Experiments. https://experiments.springernature.com/articles/10.1007/978-1-4939-6960-9_1.

[58] Koagne R.R., Annang F., Cautain B., Martín J., Pérez-Moreno G., Bitchagno G.T.M., González-Pacanowska D., Vicente F., Simo I.K., Reyes F. (2020). Cytotoxycity and Antiplasmodial Activity of Phenolic Derivatives from Albizia Zygia (DC.) J.F. Macbr. (Mimosaceae). BMC Complement Med. Ther..

[59] Audoin C., Bonhomme D., Ivanisevic J., Cruz M.D.l., Cautain B., Monteiro M.C., Reyes F., Rios L., Perez T., Thomas O.P. (2013). Balibalosides, an Original Family of Glucosylated Sesterterpenes Produced by the Mediterranean Sponge *Oscarella balibaloi*. Mar. Drugs.

[60] Vickery J.R., Whitfield F.B., Ford G.L., Kennett B.H. (1984). Ximenynic Acid in *Santalum obtusifolium* Seed Oil. J. Am. Oil Chem. Soc..

[61] Cai F., Hettiarachchi D., Hu X., Singh A., Liu Y., Sunderland B., Li D. (2022). Chapter 20—Ximenynic Acid and Its Bioactivities. Advances in Dietary Lipids and Human Health.

[62] XYMENYNIC ACID—Cosmetics Ingredient INCI. https://cosmetics.specialchem.com/inci-ingredients/xymenynic-acid.

[63] El-Jaber N., Estévez-Braun A., Ravelo A.G., Muñoz-Muñoz O., Rodríguez-Afonso A., Murguia J.R. (2003). Acetylenic Acids from the Aerial Parts of *Nanodea muscosa*. J. Nat. Prod..

[64] Li X.-C., Jacob M.R., ElSohly H.N., Nagle D.G., Smillie T.J., Walker L.A., Clark A.M. (2003). Acetylenic Acids Inhibiting Azole-Resistant Candida Albicans from *Pentagonia gigantifolia*. J. Nat. Prod..

[65] Li X.-C., Jacob M.R., Khan S.I., Ashfaq M.K., Babu K.S., Agarwal A.K., ElSohly H.N., Manly S.P., Clark A.M. (2008). Potent In Vitro Antifungal Activities of Naturally Occurring Acetylenic Acids. Antimicrob. Agents Chemother..

[66] Okada S., Zhou X.-R., Damcevski K., Gibb N., Wood C., Hamberg M., Haritos V.S. (2013). Diversity of Δ12 Fatty Acid Desaturases in Santalaceae and Their Role in Production of Seed Oil Acetylenic Fatty Acids. J. Biol. Chem..

[67] Sperling P., Lee M., Girke T., Zähringer U., Stymne S., Heinz E. (2000). A Bifunctional Δ6-Fatty Acyl Acetylenase/Desaturase from the Moss Ceratodon Purpureus. Eur. J. Biochem..

[68] Vierengel A., Kohn G., Vandekerkhove O., Hartmann E. (1987). 9-Octadecen-6-Ynoic Acid from Riccia Fluitans. Phytochemistry.

[69] Acetylenic FA|Cyberlipid. https://cyberlipid.gerli.com/description/simple-lipids/fatty-acids/acetylenic-fa/.

[70] Minto R.E., Blacklock B.J. (2008). Biosynthesis and Function of Polyacetylenes and Allied Natural Products. Prog. Lipid Res..

[71] Ondeyka J.G., Zink D.L., Young K., Painter R., Kodali S., Galgoci A., Collado J., Tormo J.R., Basilio A., Vicente F. (2006). Discovery of Bacterial Fatty Acid Synthase Inhibitors from a Phoma Species as Antimicrobial Agents Using a New Antisense-Based Strategy. J. Nat. Prod..

[72] Tobinaga S., Sharma M., Aalbersberg W., Watanabe K., Iguchi K., Narui K., Sasatsu M., Waki S. (2009). Isolation and Identification of a Potent Antimalarial and Antibacterial Polyacetylene from *Bidens pilosa*. Planta Med..

[73] da Silva J., Cerdeira C.D., Chavasco J.M., Cintra A.B.P., da Silva C.B.P., de Mendonça A.N., Ishikawa T., Boriollo M.F.G., Chavasco J.K. (2014). In vitro screening antibacterial activity of *Bidens pilosa linné* and *Annona crassiflora mart*. against oxacillin resistant *Staphylococcus aureus* (ORSA) from the aerial environment at the dental clinic. Rev. Inst. Med. Trop. Sao Paulo.

[74] Yan Z., Chen Z., Zhang L., Wang X., Zhang Y., Tian Z. (2022). Bioactive Polyacetylenes from Bidens Pilosa L and Their Anti-Inflammatory Activity. Nat. Prod. Res..

[75] Ahamad J., Ismail S.A., Jalal A.Z., Akhtar M.S., Ahmad J. (2025). Stilbenes in Breast Cancer Treatment: Nanostilbenes an Approach to Improve Therapeutic Efficacy. Pharmacol. Res. Nat. Prod..

[76] Lee Y.-H., Chen Y.-Y., Yeh Y.-L., Wang Y.-J., Chen R.-J. (2019). Stilbene Compounds Inhibit Tumor Growth by the Induction of Cellular Senescence and the Inhibition of Telomerase Activity. Int. J. Mol. Sci..

[77] Sirerol J.A., Rodríguez M.L., Mena S., Asensi M.A., Estrela J.M., Ortega A.L. (2016). Role of Natural Stilbenes in the Prevention of Cancer. Oxidative Med. Cell. Longev..

[78] Tian J., Jin L., Liu H., Hua Z. (2023). Stilbenes: A Promising Small Molecule Modulator for Epigenetic Regulation in Human Diseases. Front. Pharmacol..

[79] Piekuś-Słomka N., Mikstacka R., Ronowicz J., Sobiak S. (2019). Hybrid Cis-Stilbene Molecules: Novel Anticancer Agents. Int. J. Mol. Sci..

[80] Breast Cancer Prevention: Tamoxifen and Raloxifene. https://www.cancer.org/cancer/types/breast-cancer/risk-and-prevention/tamoxifen-and-raloxifene-for-breast-cancer-prevention.html.

