# Supplementary material for: Acetylenic Fatty Acids and Stilbene Glycosides Isolated from Santalum yasi Collected from the Fiji Islands
Source: Molecules. 2025 Dec 12;30(24):4752. doi: 10.3390/molecules30244752 (PMC12736004; doi:10.3390/molecules30244752)
Supplement: Supplementary file 1 [file molecules-30-04752-s001.zip › molecules-3738890-supplementary.pdf]

Supporting Materials

Acetylenic Fatty Acids and Stilbene Glycosides Isolated from *Santalum yasi* Collected from the Fiji Islands

Khalid AL Maqbali, Miriama Vuiyasawa, Mercy Ayinya Gube-Ibrahim, Shubham Sewariya, Clément Balat, Kirsti Helland, Tamar Garcia-Sorribes, Mercedes de la Cruz, Bastien Cautain, Jeanette Hammer Andersen, Fernando Reyes, Jioji N. Tabudravu*

Abstract

In our continuing search for new anticancer and/or antimicrobial compounds from natural products, we screened for these activities in bark and leaf extracts of sandalwood plants collected from the Fiji Islands and found *Santalum yasi* to be the most active. Resulting chemical workup enabled the isolation and structural characterization of a new acetylenic acid, methyl (*E*)-octadec-6-en-8-ynoate (**1**), and an atropisomeric stilbene glycoside (**4**) (Yasibeneoside) together with six known compounds: 11,13-octadecadien-9-ynoic acid (**2**), methyl octadeca-9,11-diynoate (**3**), gaylussacin (**5**) chrysin-7-beta-monoglucoside (**6**), neoschaftoside (**7**), and chrysin-6-C-glucoside-8-C-arabinoside (8). Compound **1** (18:2 (6t, 8a) is an example of a Δ^6, Δ^8 acetylenic system containing the *trans* double bond at C-6 and the triple bond at C-8, which is reported here for the first time. All molecular structure elucidations and dereplications were performed using spectroscopic techniques, including 2D NMR and HRMS-MS/MS spectrometry. Methyl (*E*)-octadec-6-en-8-ynoate showed moderate activity with an IC_{50} of 91.2 ug/mL against the human breast adenocarcinoma cell line MCF-7.

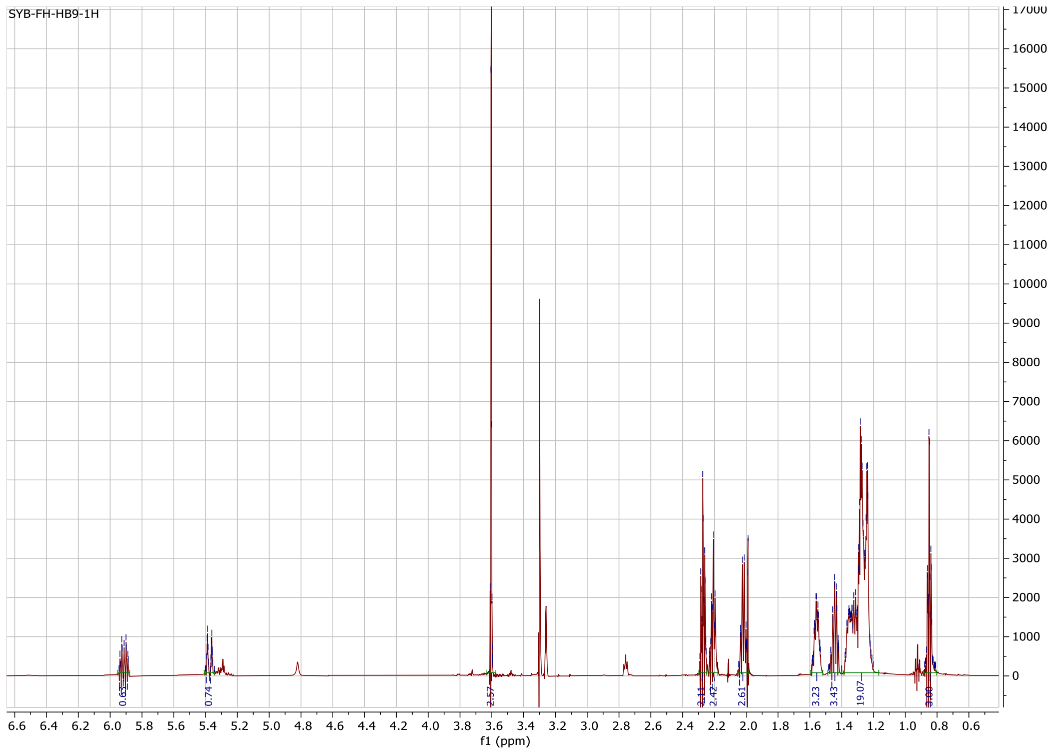

Figure S1. ^{1}H spectrum for **1** in CD_3OD at 600 MHz.

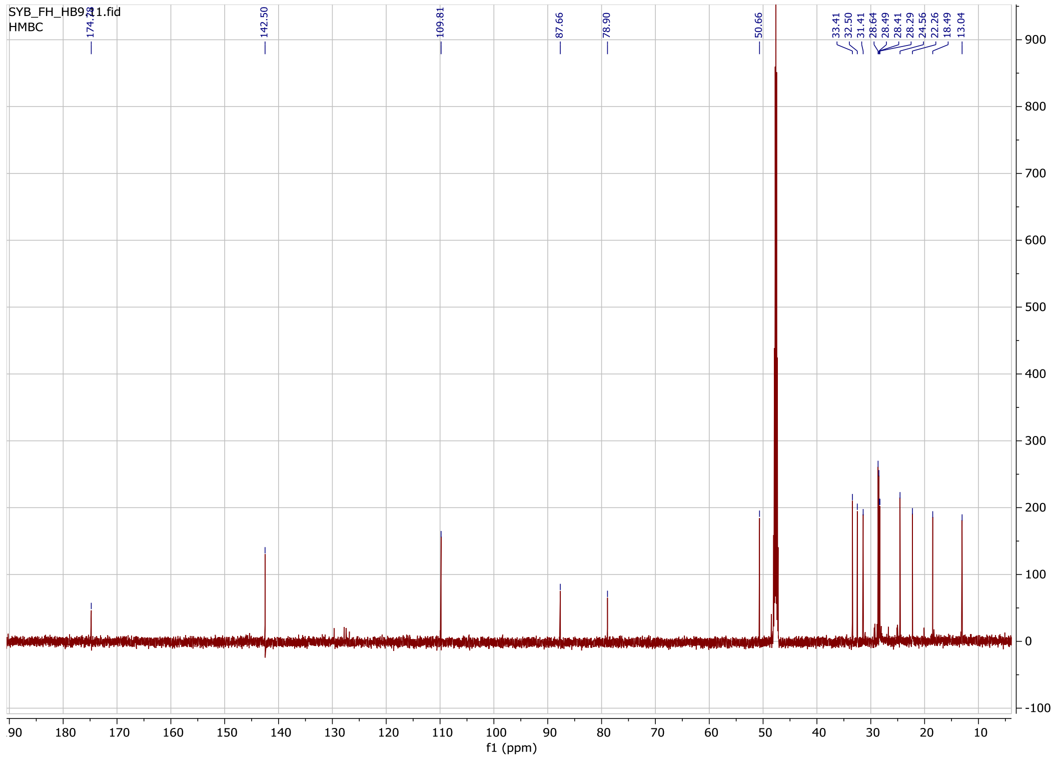

Figure S2. ^{13}C spectrum for **1** in CD_3OD at 150 MHz.

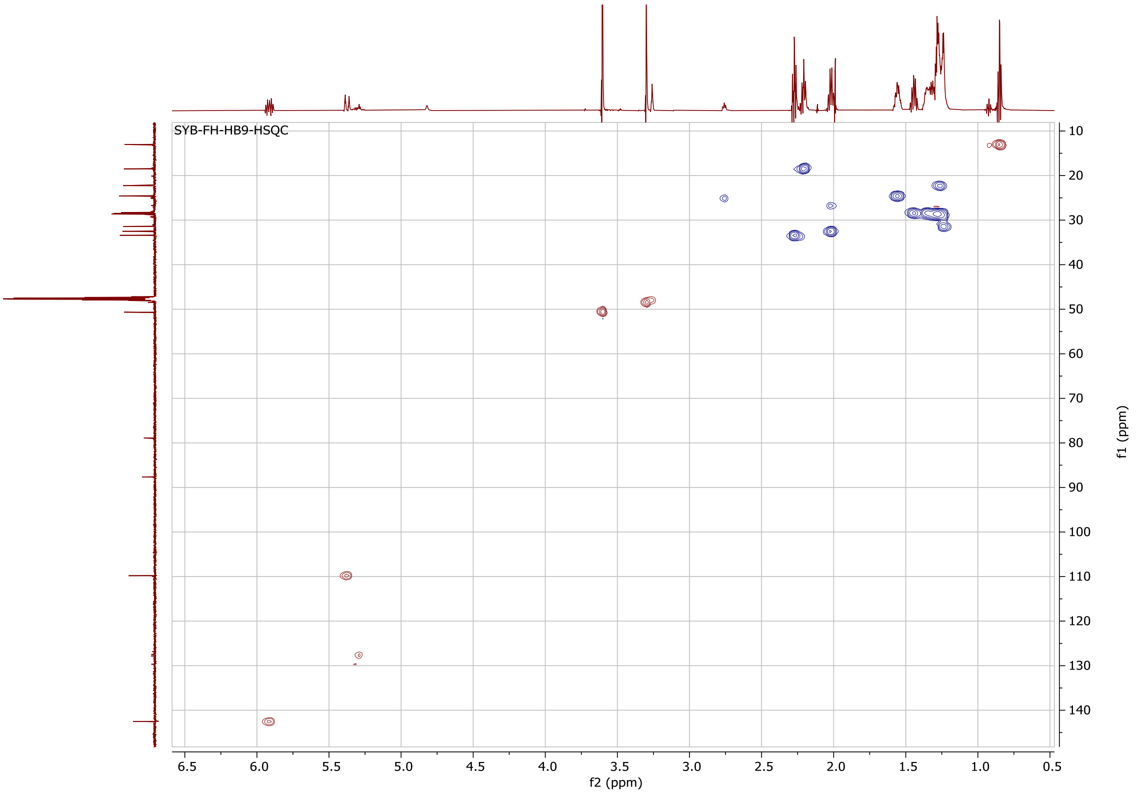

Figure S3. Edited-HSQC spectrum for **1** in CD_3OD at 600 MHz.

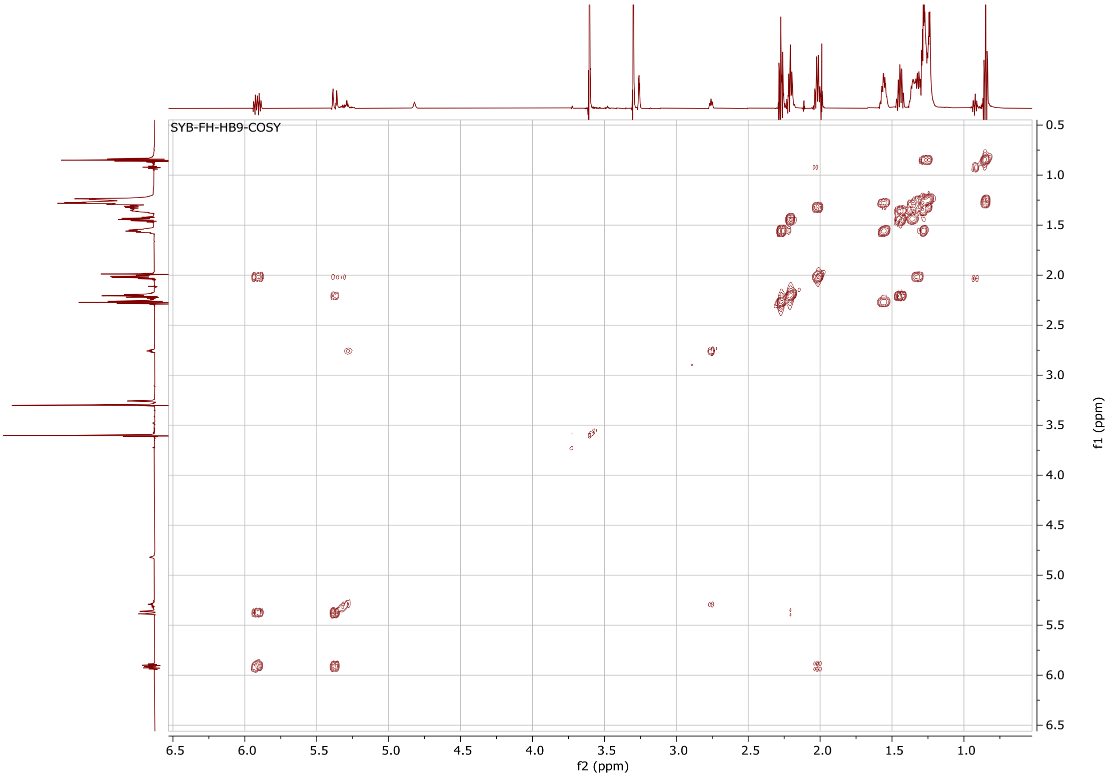

Figure S4. COSY spectrum for **1** in CD_3OD at 600 MHz.

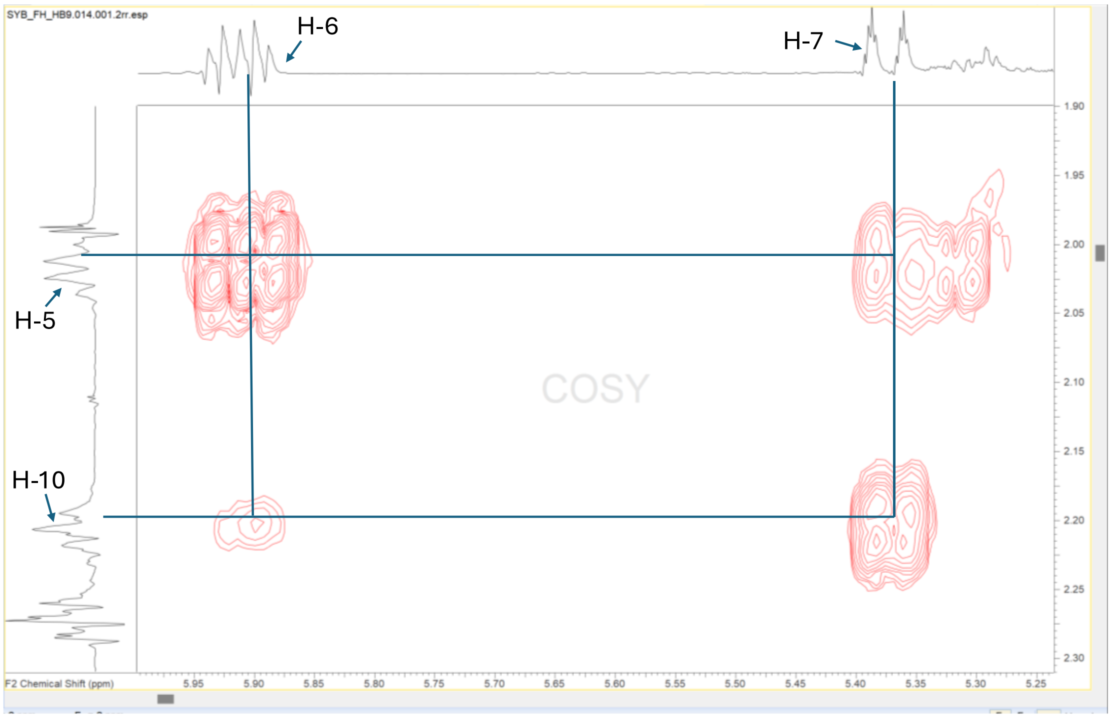

Figure S5. COSY spectrum for **1** in CD_3OD at 600 MHz confirming position of unsaturated double bond at C-5/C-6. Stronger correlations between H-6 and H-5 indicating ^3J coupling. Other correlations are non-standard COSY correlations.

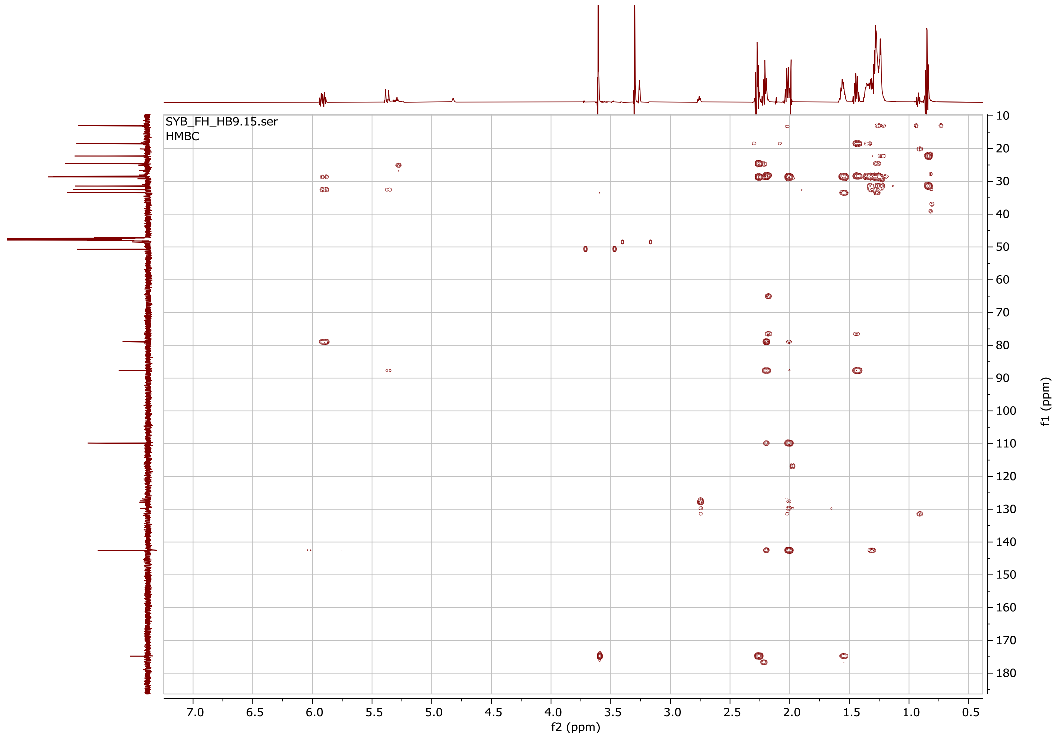

Figure S6. HMBC spectrum for **1** in CD_3OD at 600 MHz.

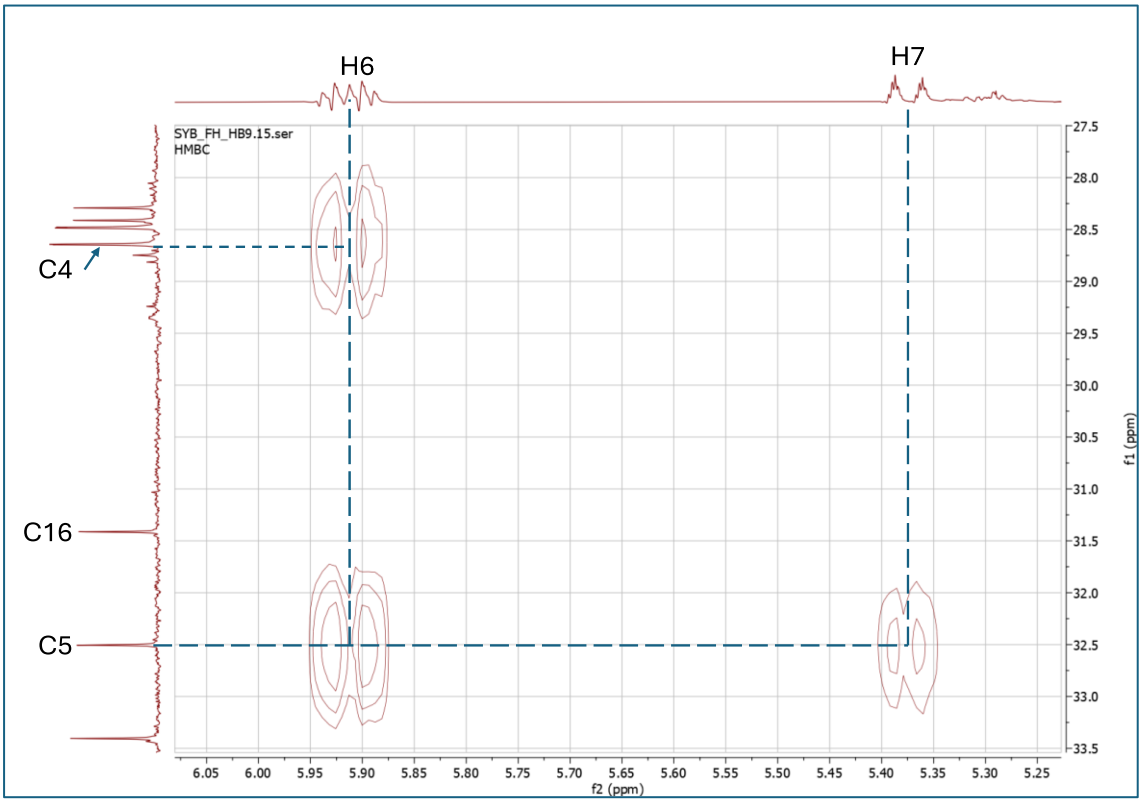

Figure S7. HMBC correlations between H-6/H-7 to the carbon at C-5; H-6 to C-4/C-5 for **1** establishing the alkene unsaturation at C6/C7.

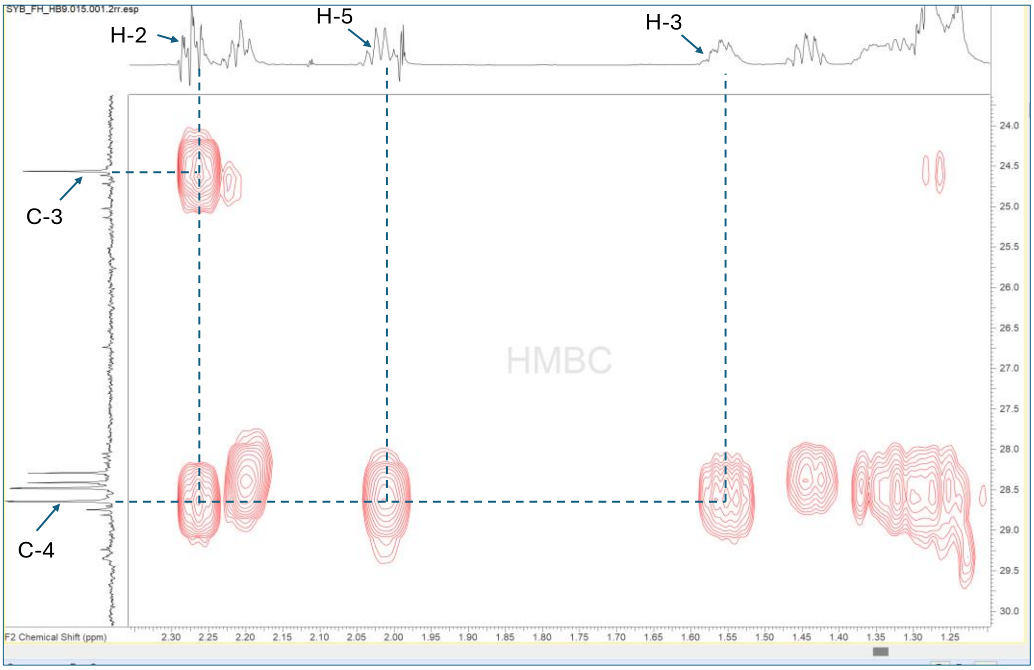

Figure S8. HMBC correlations between H-2/H-3/H-5 to C-4; H-2 to C-3 for **1** established position of alkene unsaturation at C-6/C-7.

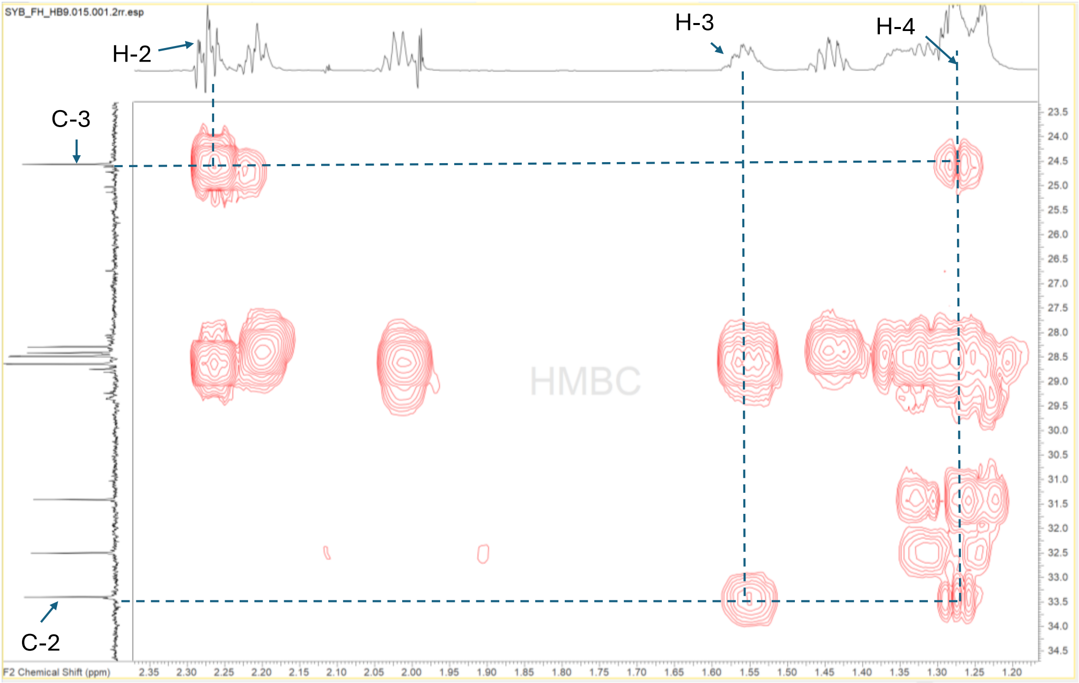

Figure S9. HMBC correlations between H-2/H-4 to C-3; H-3/H-4 to C-2 for **1** established the alkene system was at C-6/C-7, and the triple bond at C-8/C-9.

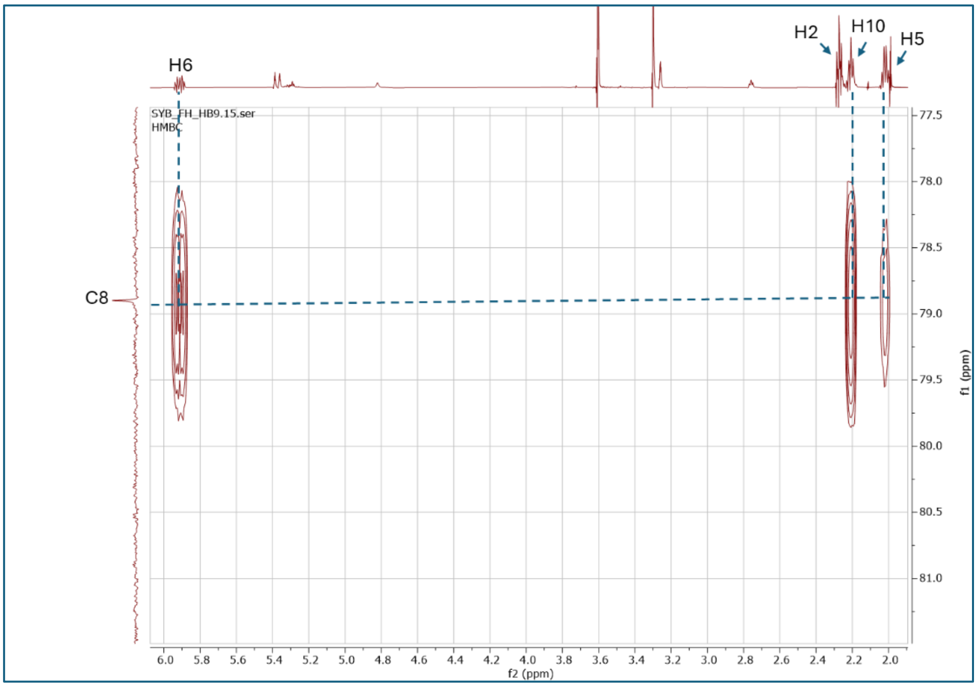

Figure S10. HMBC correlations between H-5/H-6/H-10 to the carbon at C-8 for **1** establishing alkyne unsaturation at C8/C9.

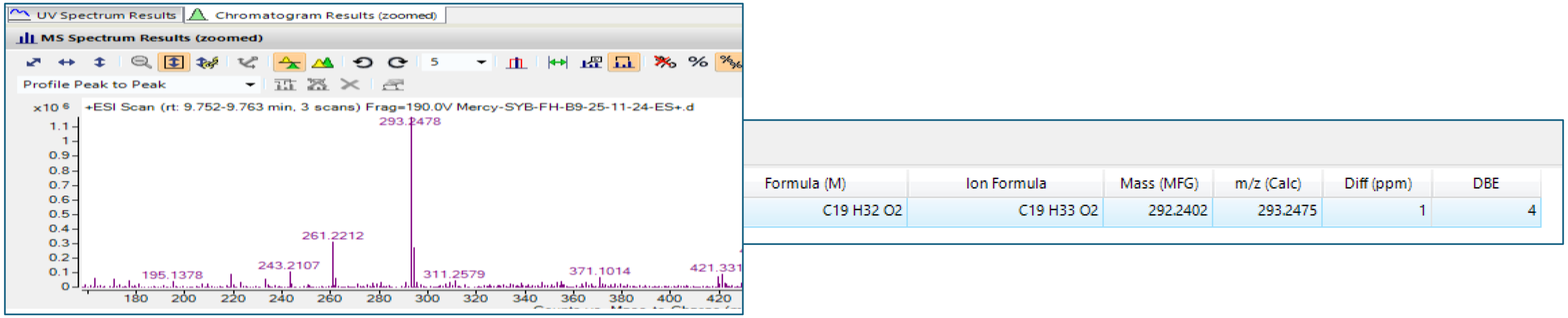

Formula (M)	Ion Formula	Mass (MFG)	m/z (Calc)	Diff (ppm)	DBE
C19 H32 O2	C19 H33 O2	292.2402	293.2475	1	4

Figure S11. Extracted ion chromatogram (EIC), HR-ESIMS (ES^+) spectrum, and molecular formula calculation for **1** using Agilent Mass Hunter softwar[1].

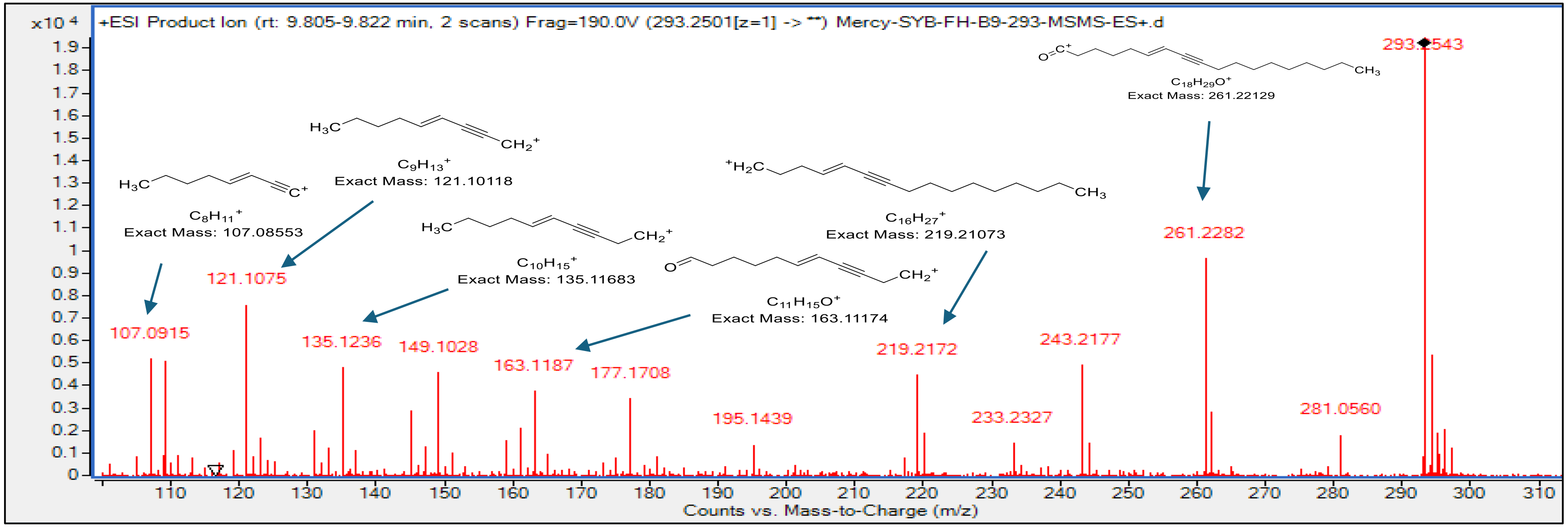

Figure S12. HR-ESIMS-MS/MS (ES^+) spectrum for MZ 293.1478 and assignment of fragments using ACD/Labs MS Fragmenter[2] for **1**.

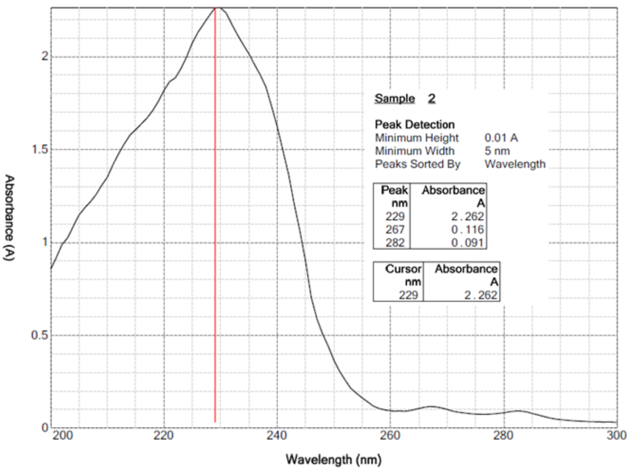

Figure S13. IR spectrum UV spectrum for **1** in MeOH.

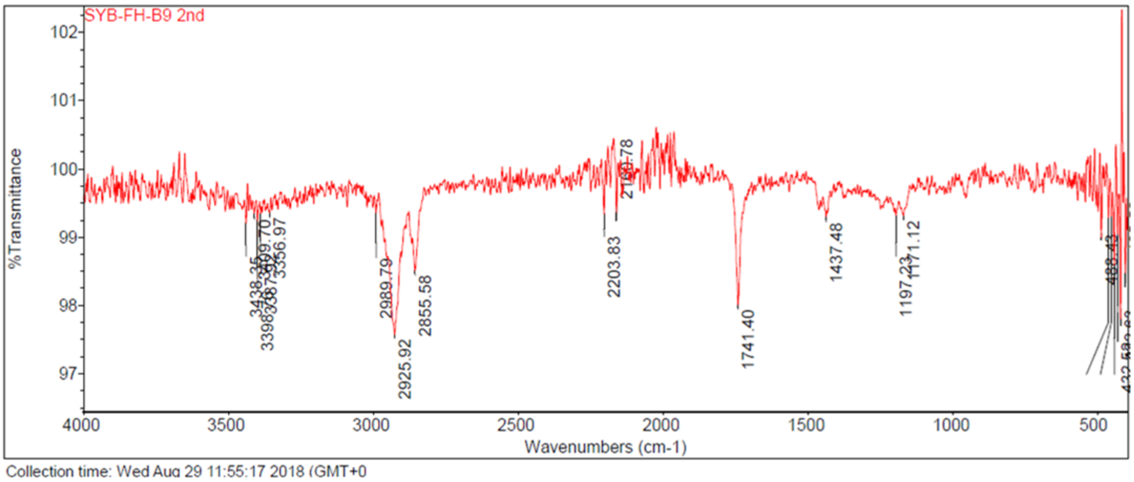

Figure S14. IR spectrum for compound **1** in MeOH.

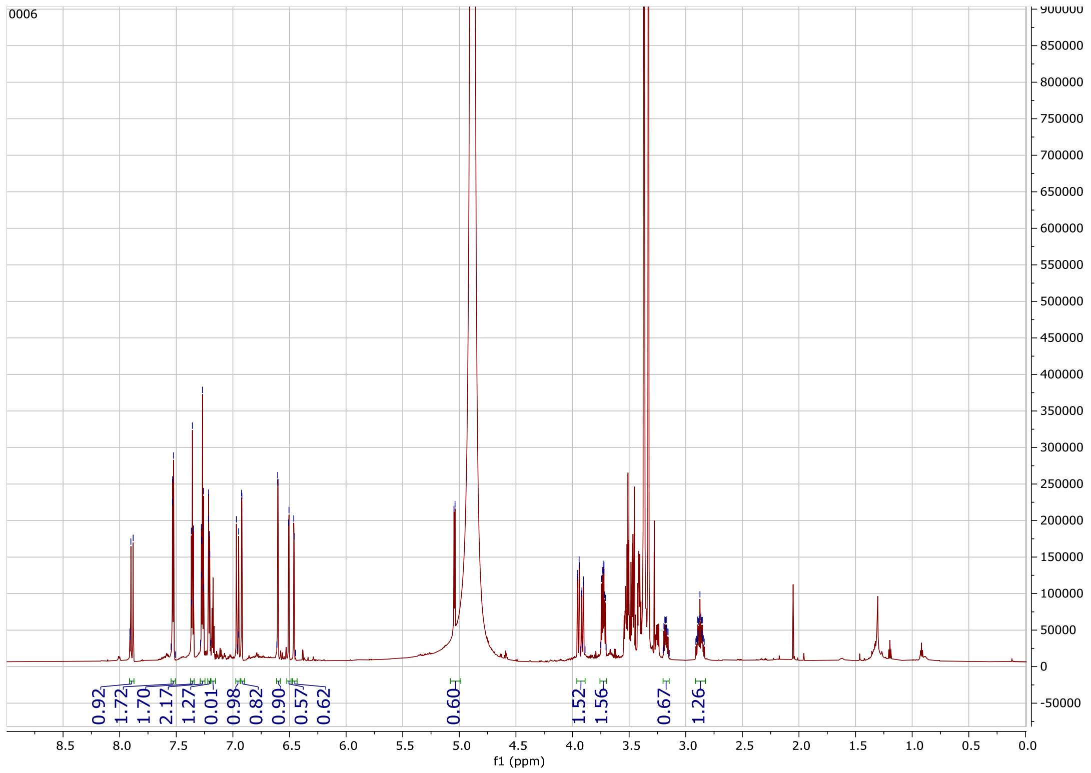

Figure S15. ^{1}H NMR spectrum for **4** in CD_3OD at 600 MHz.

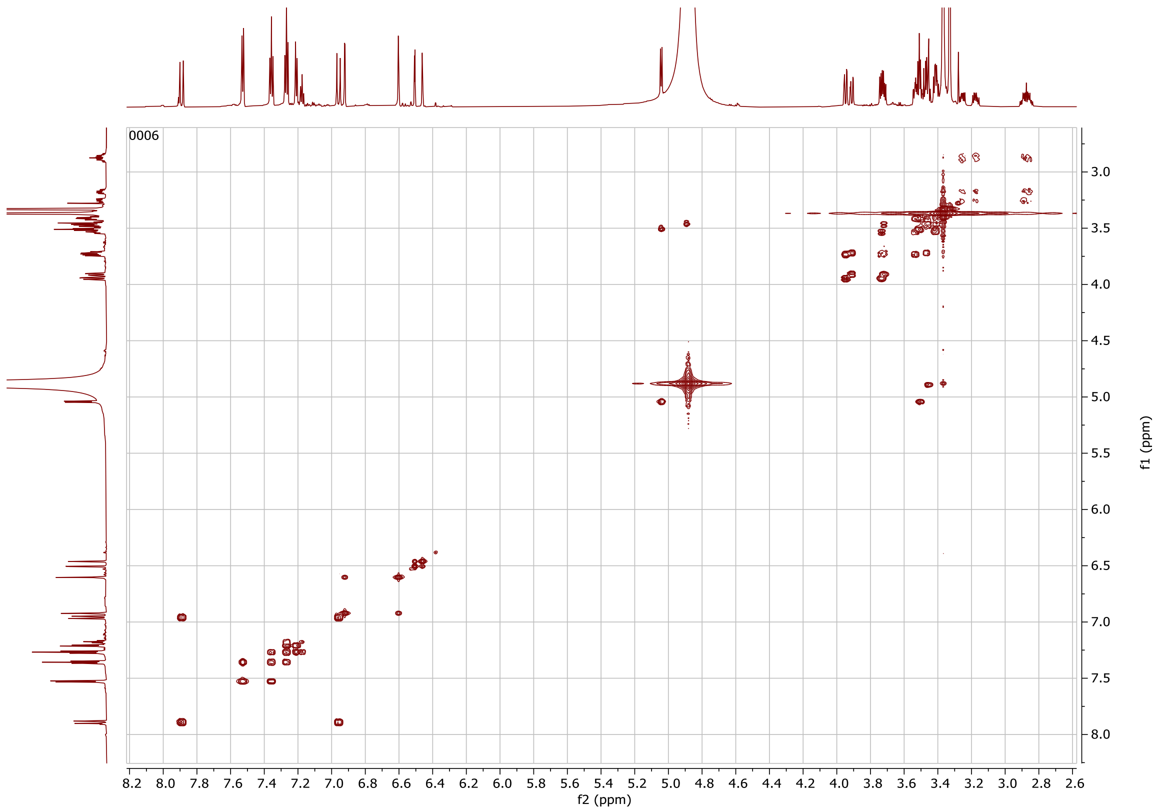

Figure S16. ^{13}C NMR spectrum for **4** in CD_3OD at 150 MHz.

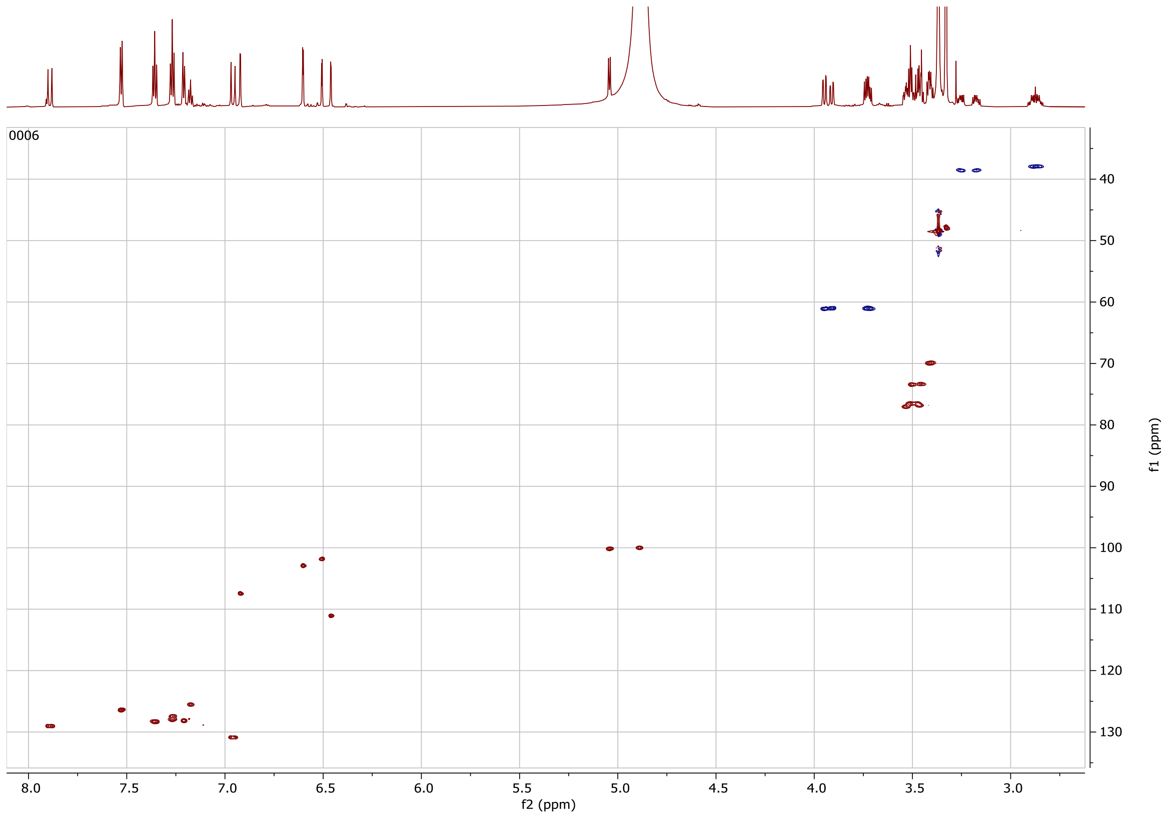

Figure S17. Edited-HSQC spectrum for **4** in CD_3OD at 600 MHz.

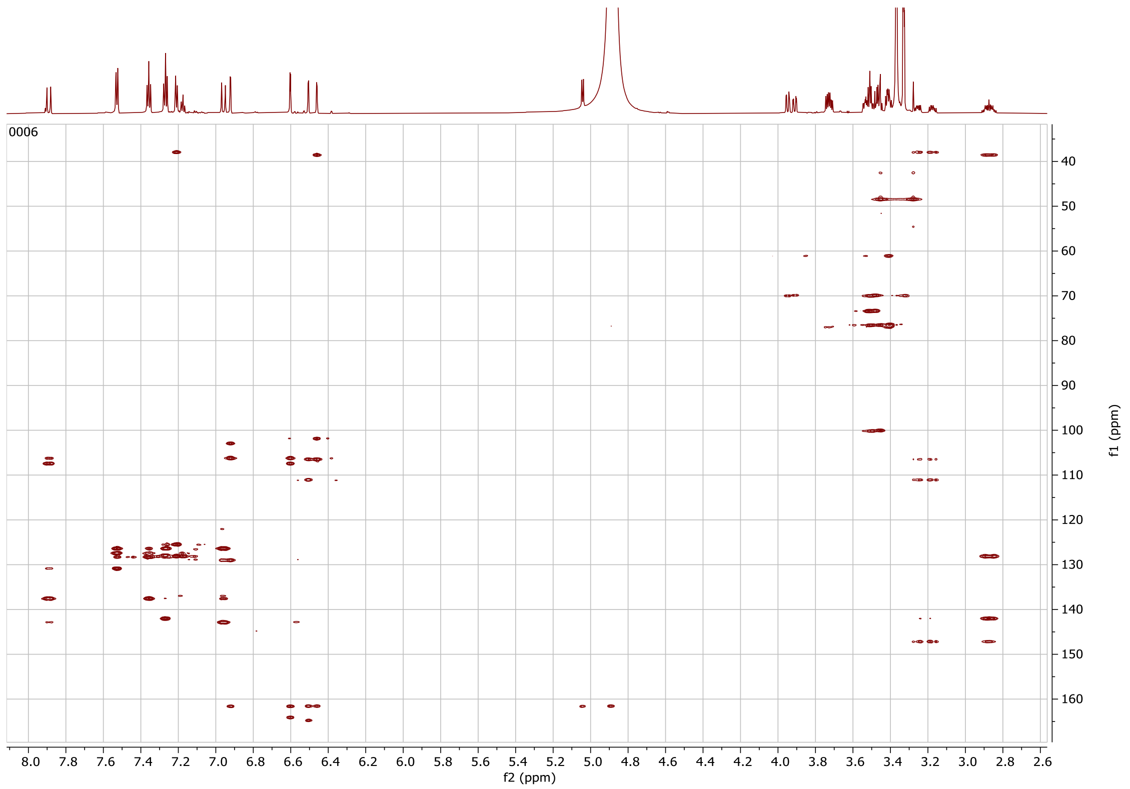

Figure S18. HMBC spectrum for **4** in CD_3OD at 600 MHz.

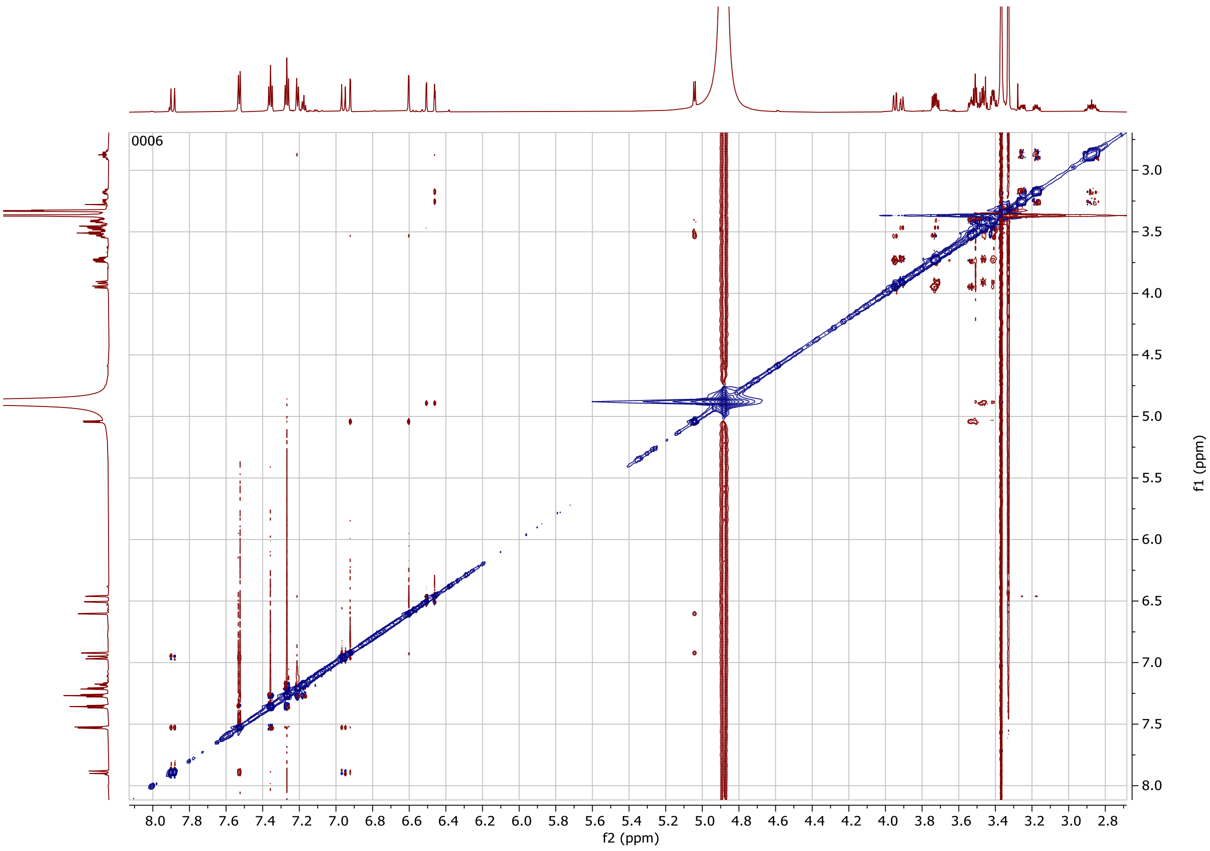

Figure S19. 2D ROESY spectrum for **4** in CD_3OD at 600 MHz.

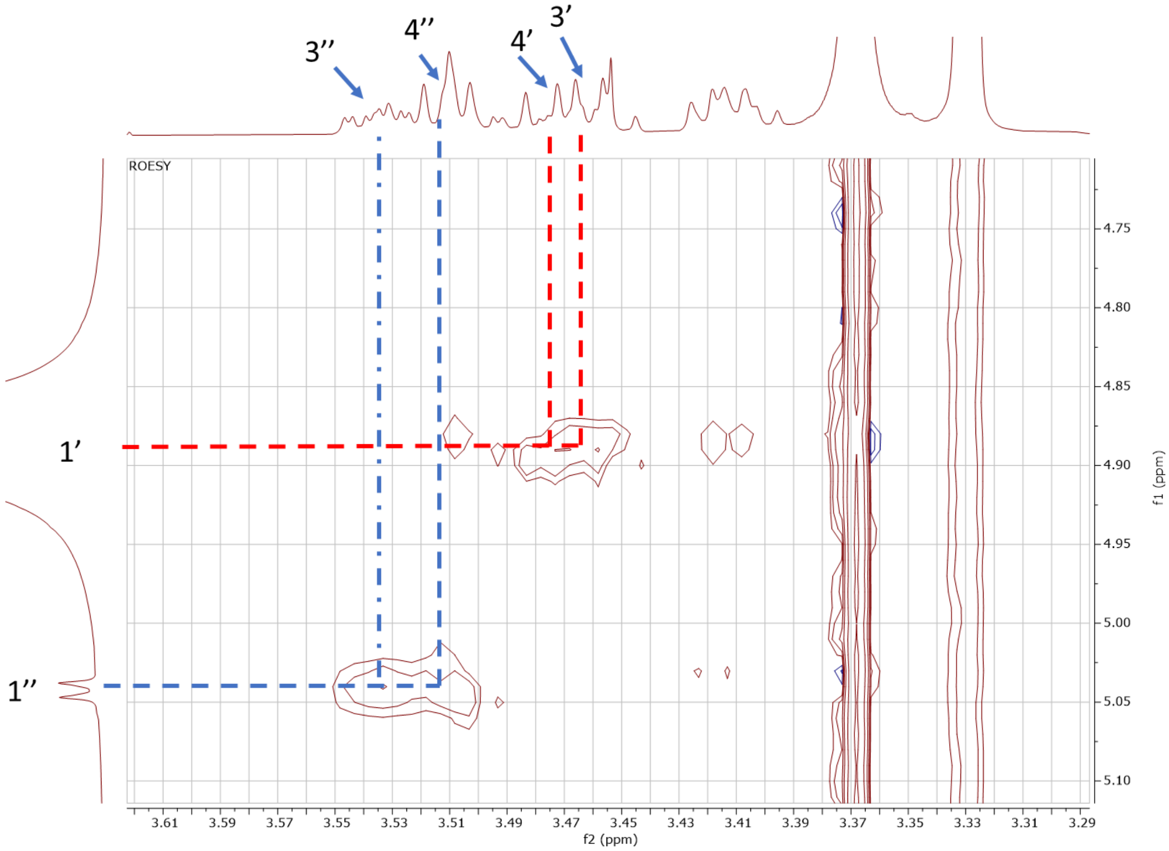

Figure S20. NOESY correlations for glycosidic anomeric protons for **4.**

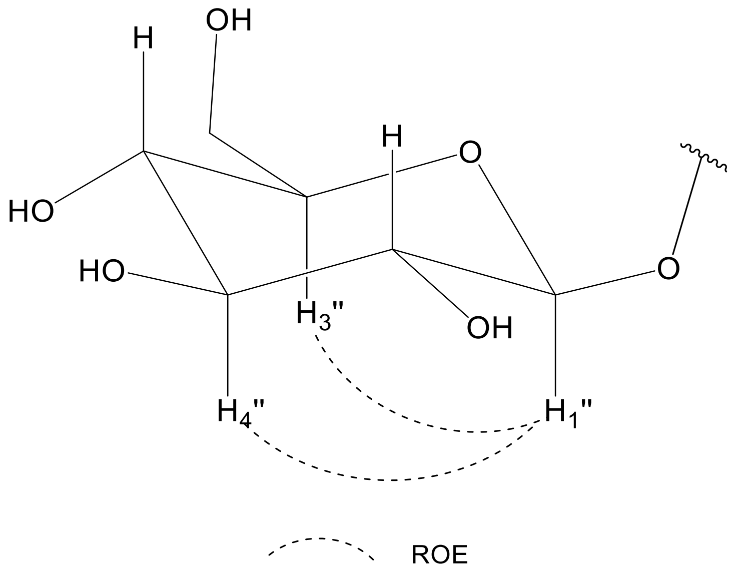

Figure S21. Relative configuration for glycoside 2'' in **4.**

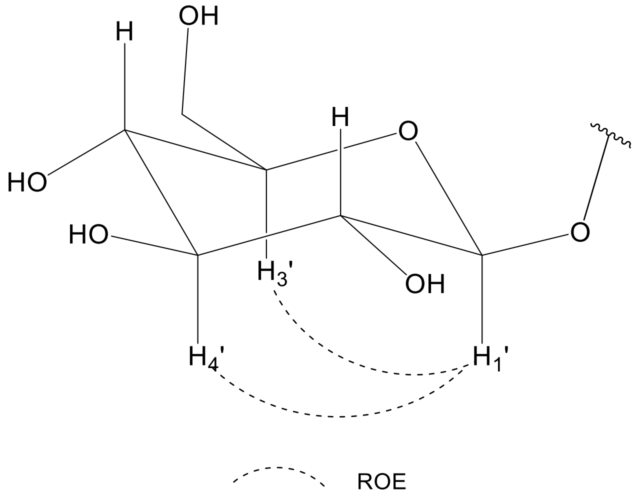

Figure S22. Relative configuration for glycoside 1' in **4.**

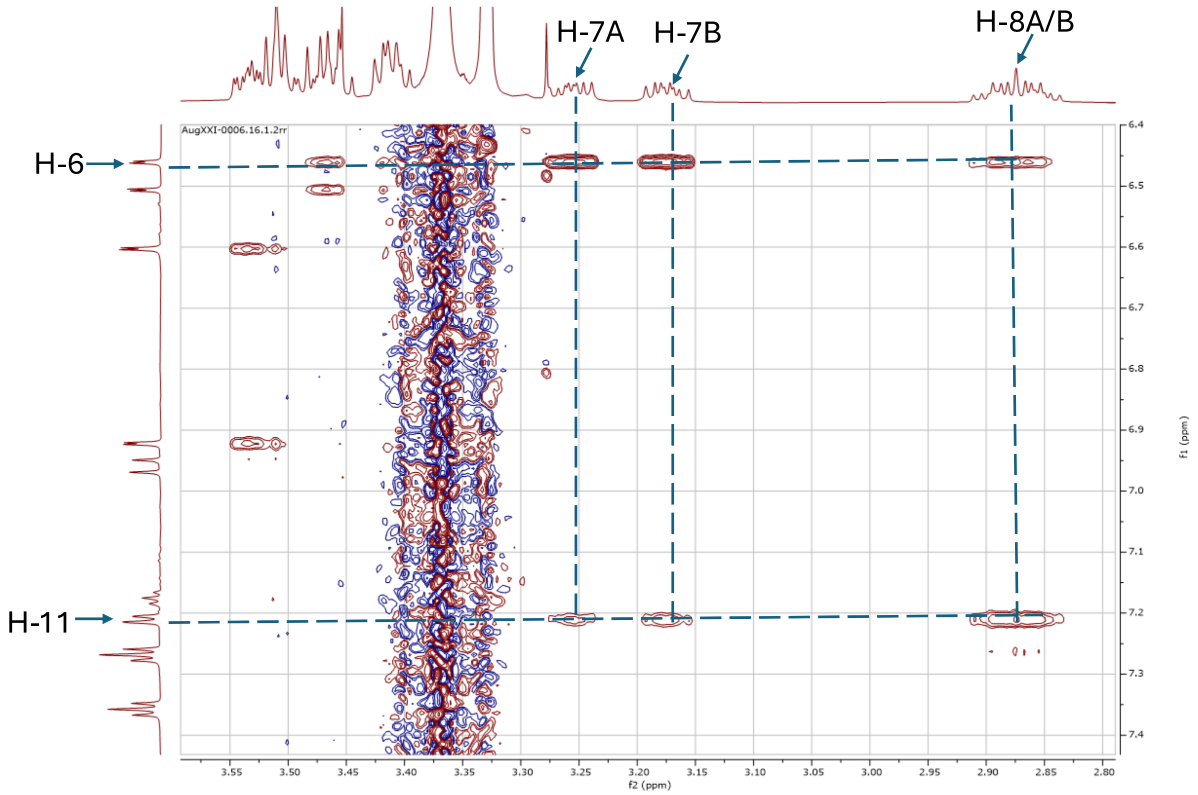

Figure S23. 2D ROESY spectrum at 600 MHz in CD_3OD for compound 4 showing key NOE correlations (Figure S24).

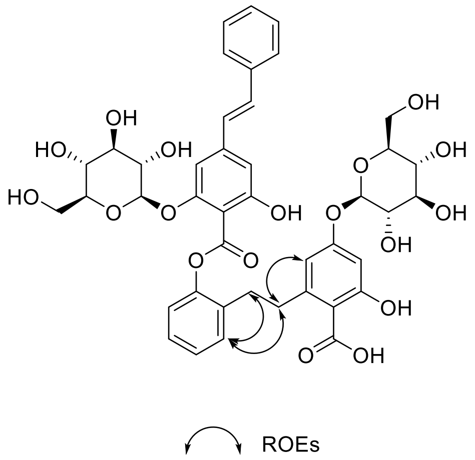

Figure S24. Key observed ROEs in structure of 4.

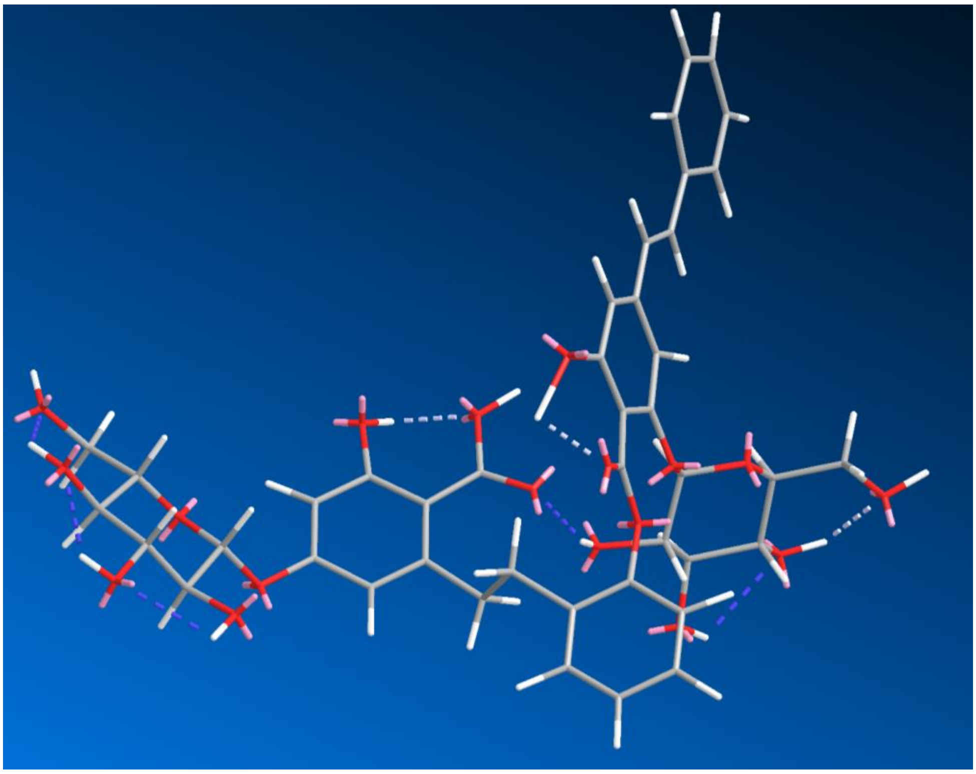

Figure S25. Key hydrogen bonding between carbonyl groups at C-1 to -OH at C-4'' reducing free rotation leading to atropisomerism in **4** as observed in ^{1}H NMR diastereotopic splitting of H-8/H-9 (Figures S15-S19).

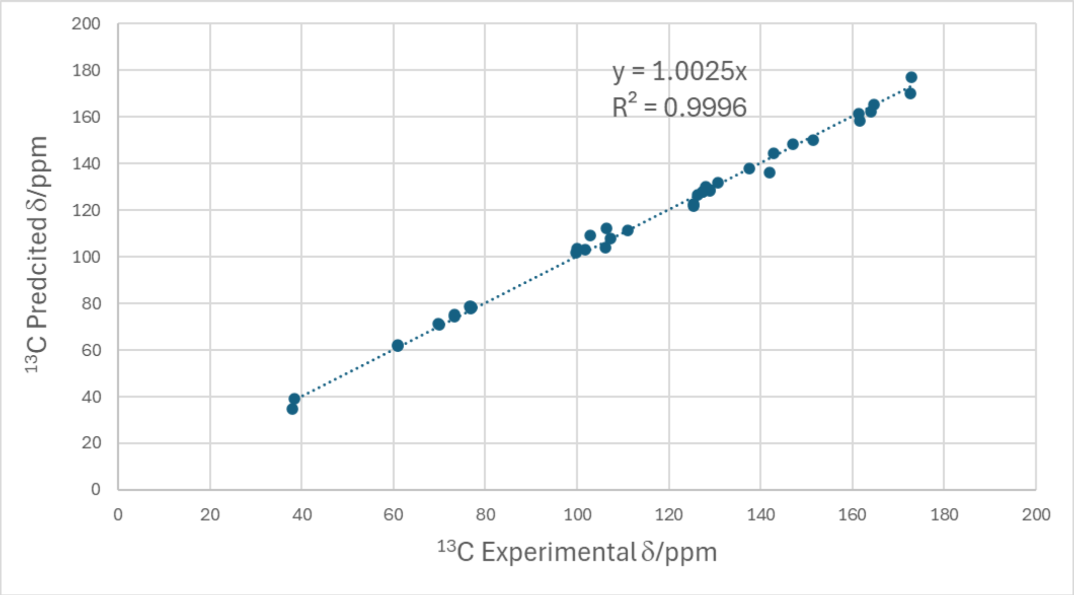

Figure S26. ^{13}C experimental vs predicted (δ/ppm) for **4.**

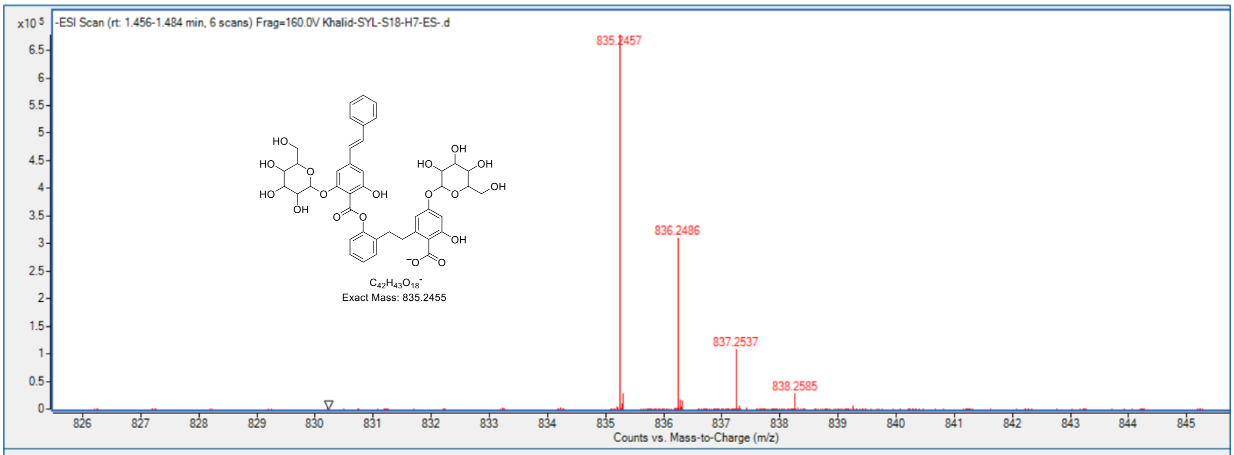

Formula (M)	Ion Formula	Mass (MFG)	m/z (Calc)	Diff (ppm)	DBE
C42 H44 O18	C42 H43 O18	836.2528	835.2455	0.25	21
C60 H36 O5	C60 H35 O5	836.2563	835.249	-3.94	43

Figure S27. HR-ESIMS (ES$^-$) spectrum and molecular formula generated by Agilent Tech. Mass Hunter[1] for compound **4.**

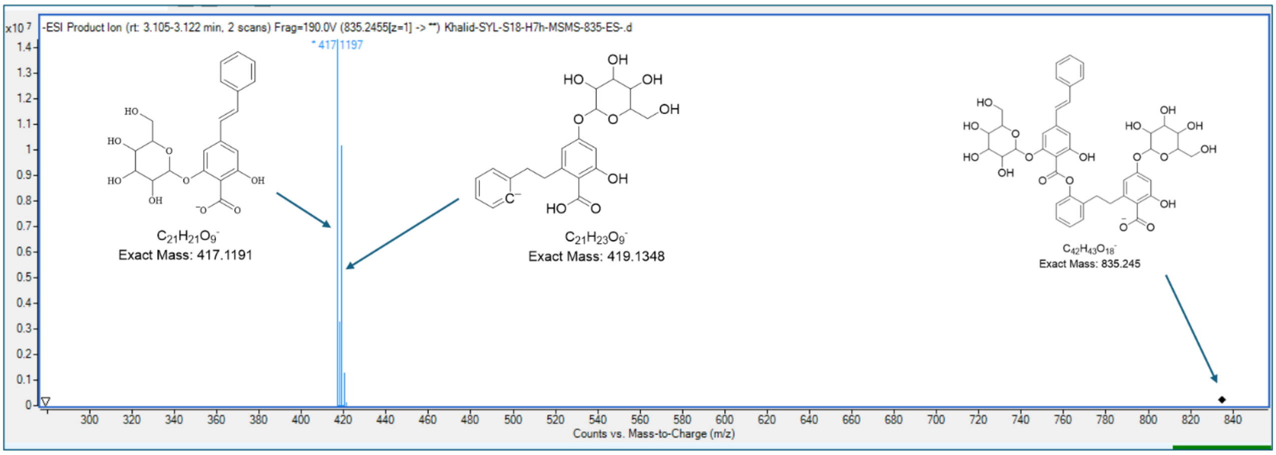

Figure S28. HR-ESIMS-MS/MS (ES$^-$) fragmentation spectrum and annotations of structural fragments for compound **4** using ACD/Labs Fragmenter.[2]

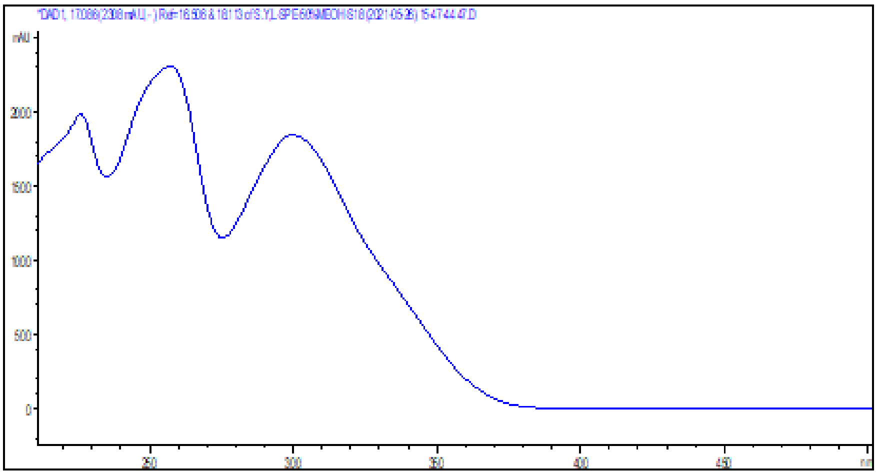

Figure S29. UV spectrum for **4** in CH_3CN-H_2O (60-40).

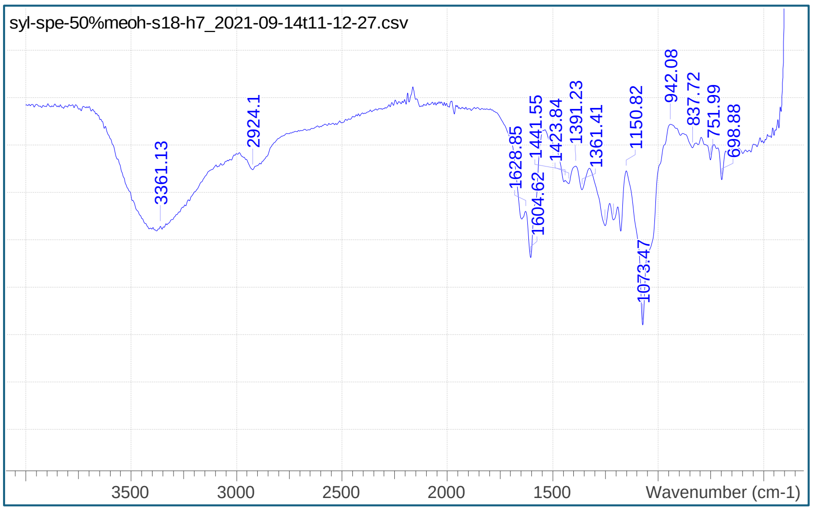

Figure S30. IR spectrum for **4** taken in MeOH.

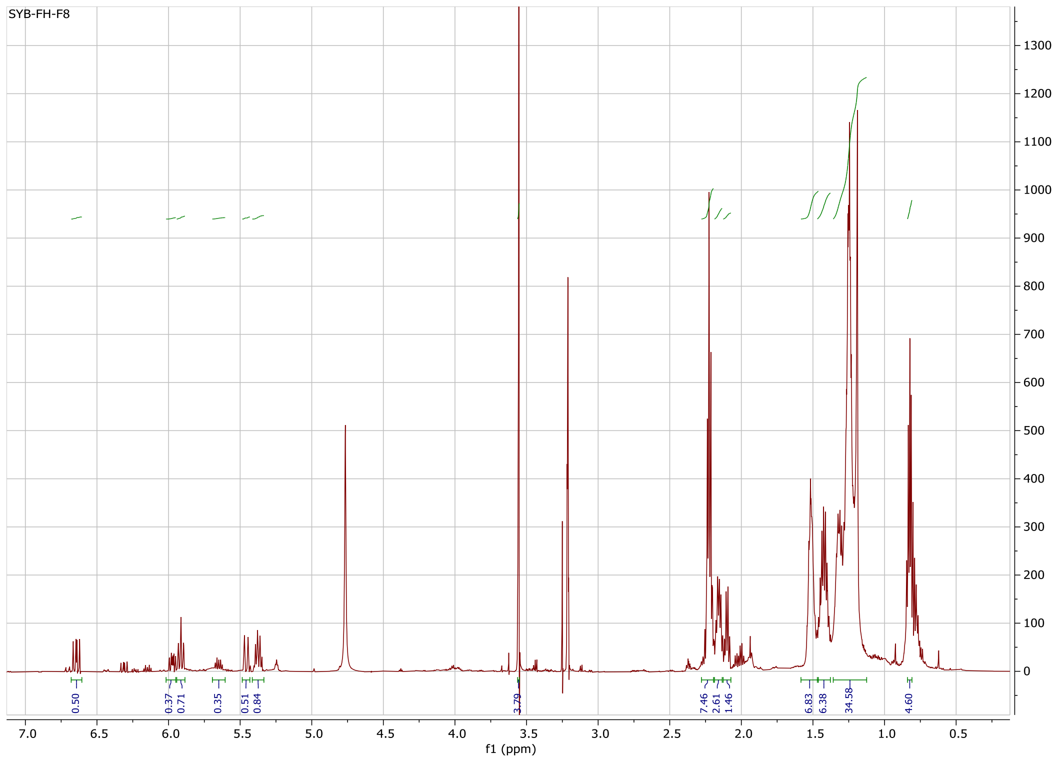

Figure S31. ^{1}H spectrum for compound **2** in CD_3OD at 600 MHz.

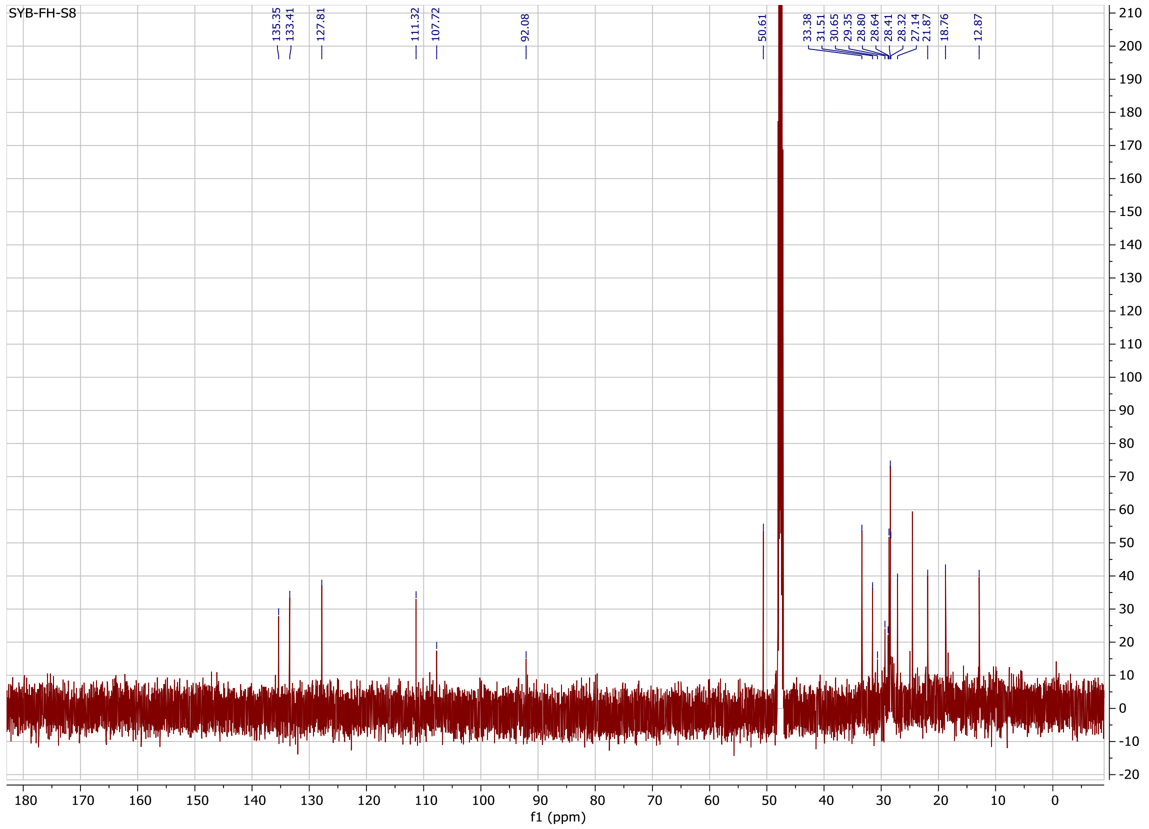

Figure S32. ^{13}C spectrum for compound **2** in CD_3OD at 150 MHz.

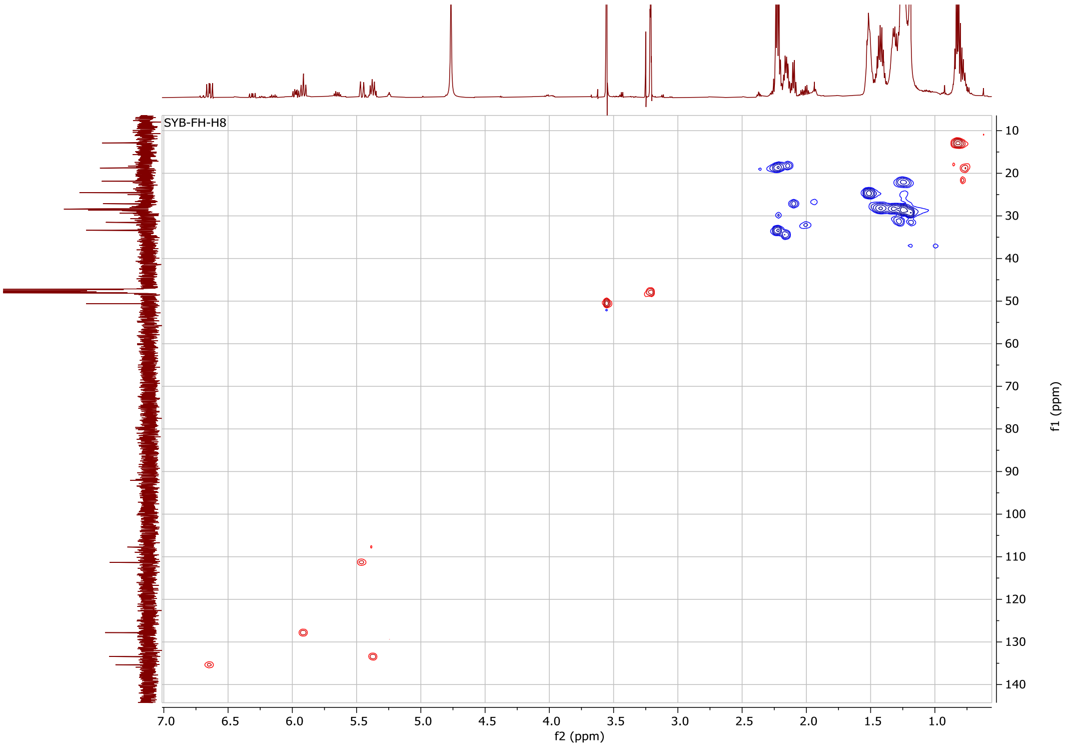

Figure S33. Edited-HSQC spectrum for compound **2** in CD_3OD at 600 MHz.

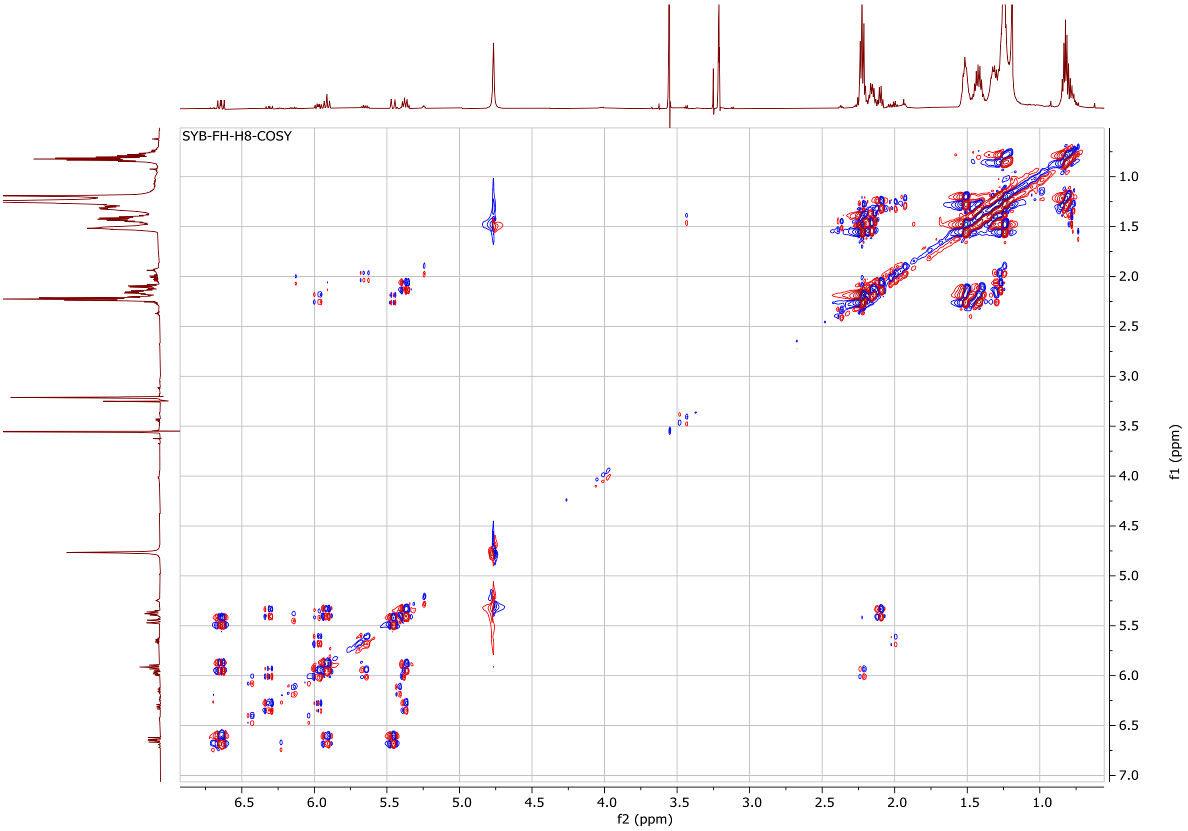

Figure S34. COSY spectrum for compound **2** in CD_3OD at 600 MHz.

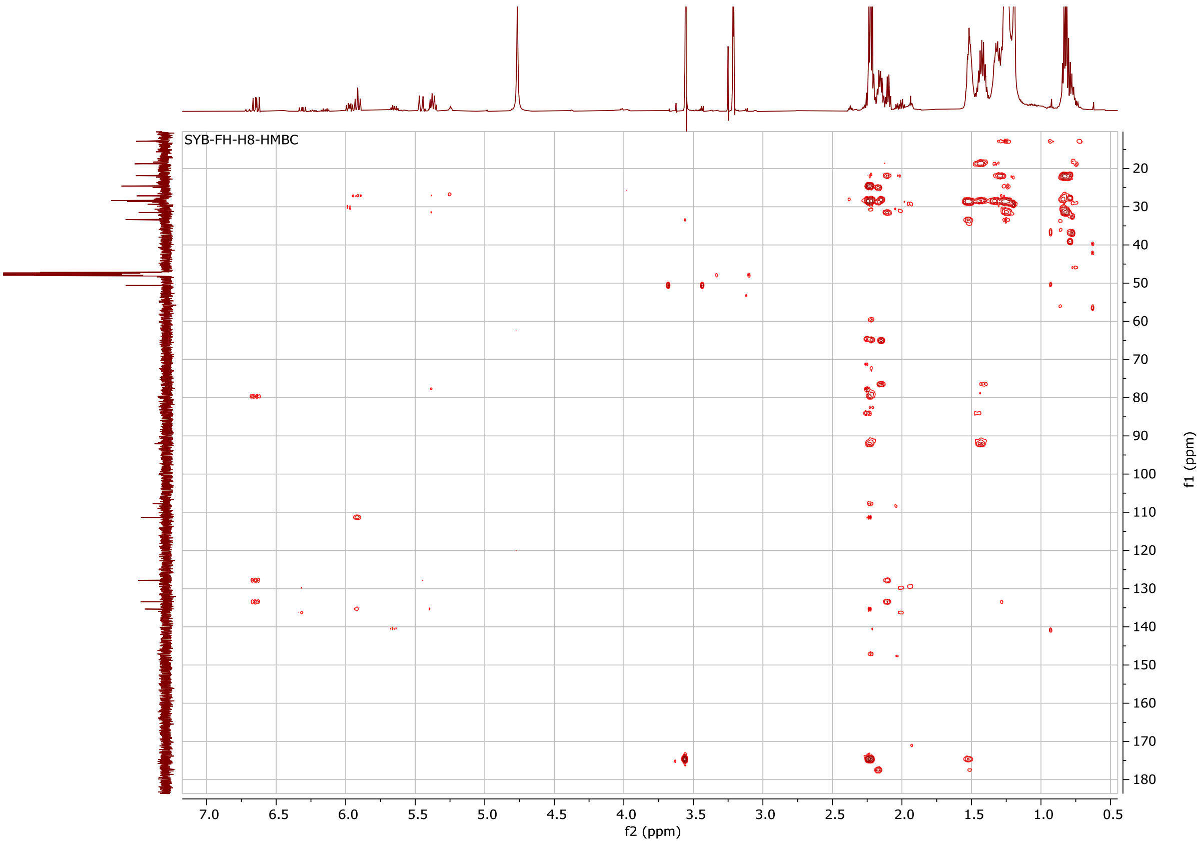

Figure S35. HMBC spectrum for compound **2** in CD_3OD at 600 MHz.

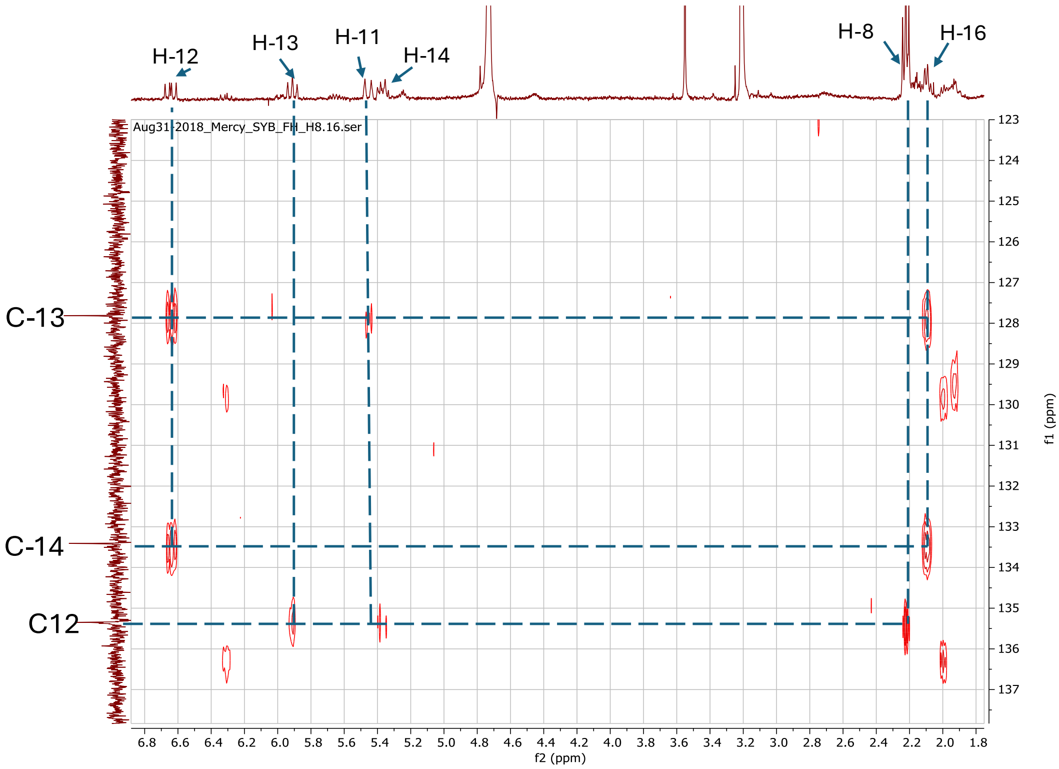

Figure S36. HMBC correlations establishing position of alkene system toward terminal methyl group end of **2**.

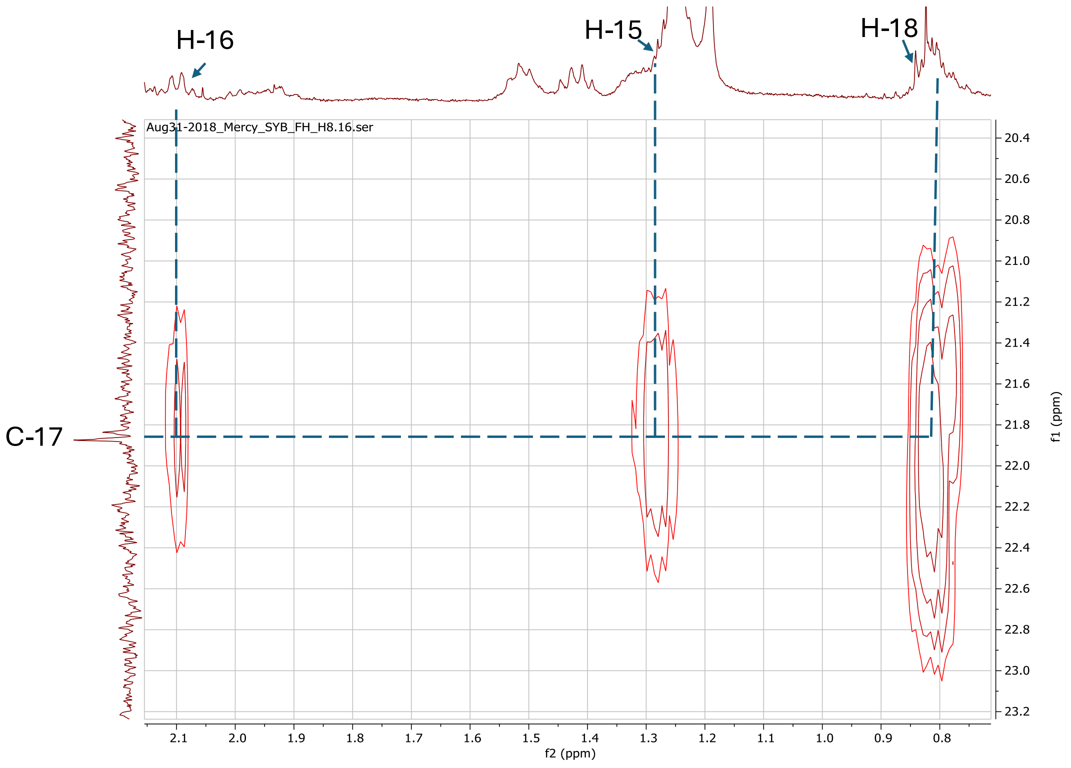

Figure S37. HMBC correlations establishing position of alkene system toward terminal methyl end of **2**.

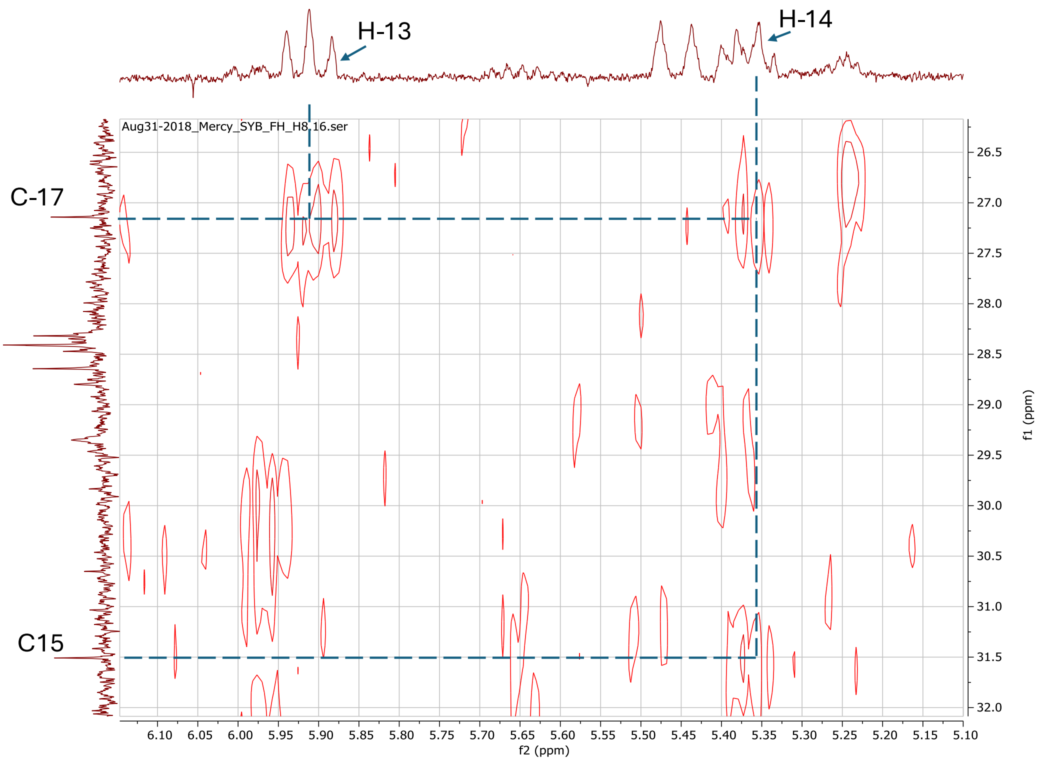

Figure S38. HMBC correlations establishing position of alkene system toward terminal methyl end of **2.**

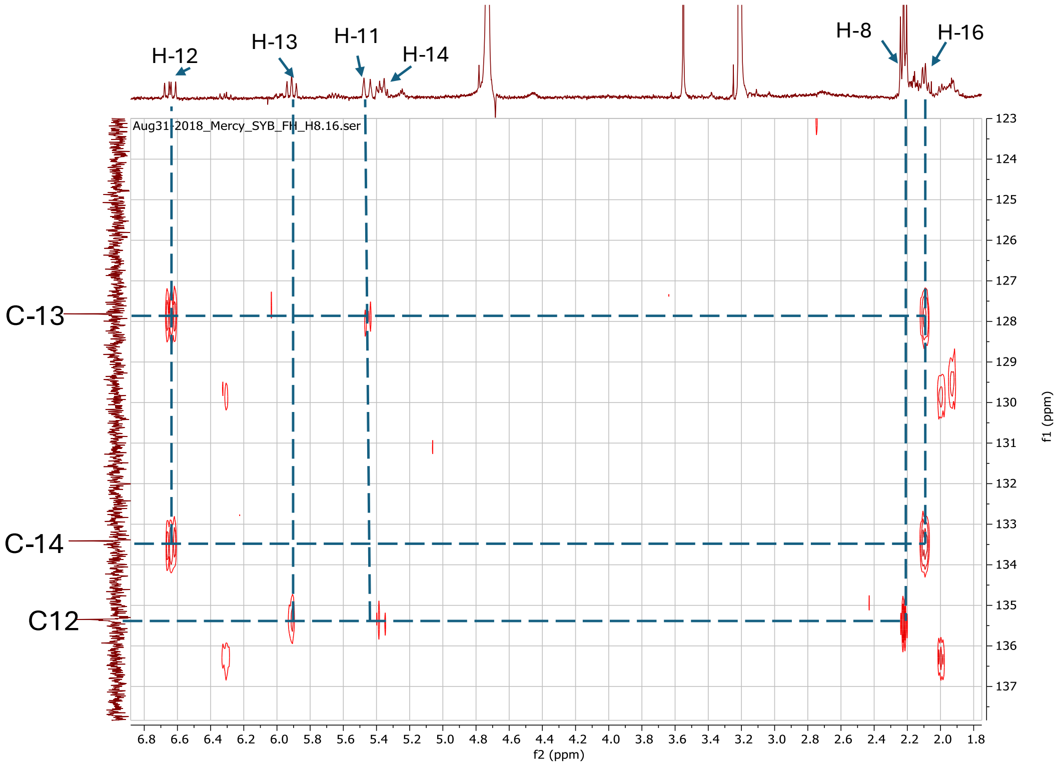

Figure S39. HMBC correlations establishing position of alkene system toward terminal methyl end of **2.**

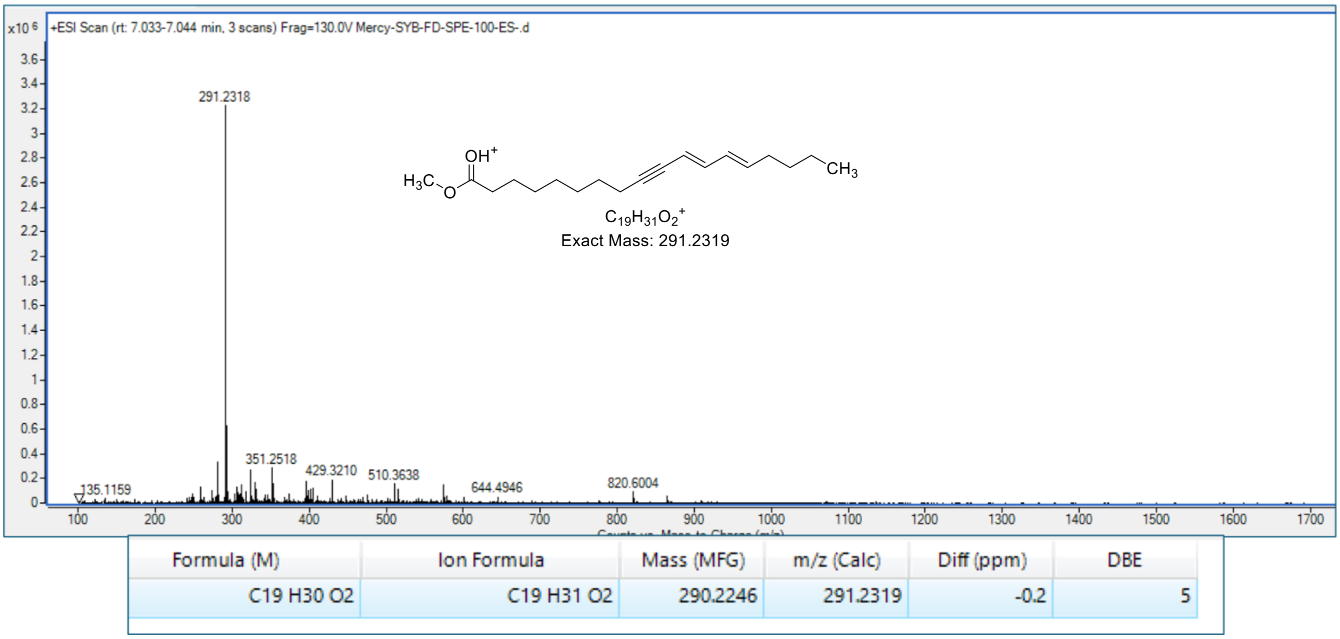

Formula (M)	Ion Formula	Mass (MFG)	m/z (Calc)	Diff (ppm)	DBE
C19 H30 O2	C19 H31 O2	290.2246	291.2319	-0.2	5

Figure S40. HR-ESIMS (ES^+) spectrum showing molecular ion and molecular formula generated by Agilent Mass Hunter software for compound **2.**

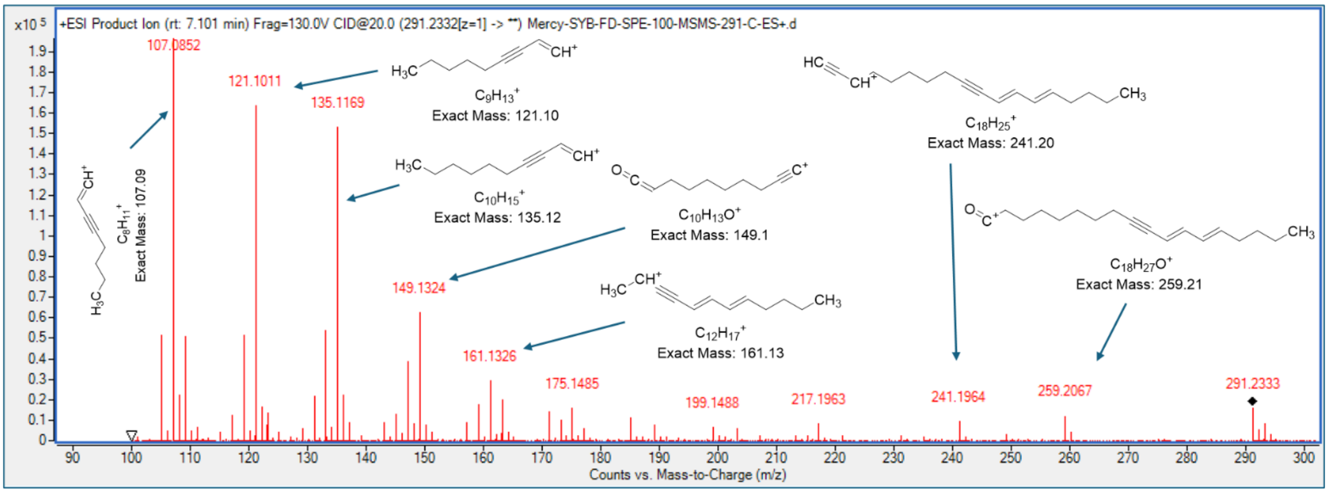

Figure S41. HR-ESIMS-MS/MS spectrum for m/z 291.2332 for **2** and fragment assignment using ACD/Labs MS Fragmenter[2].

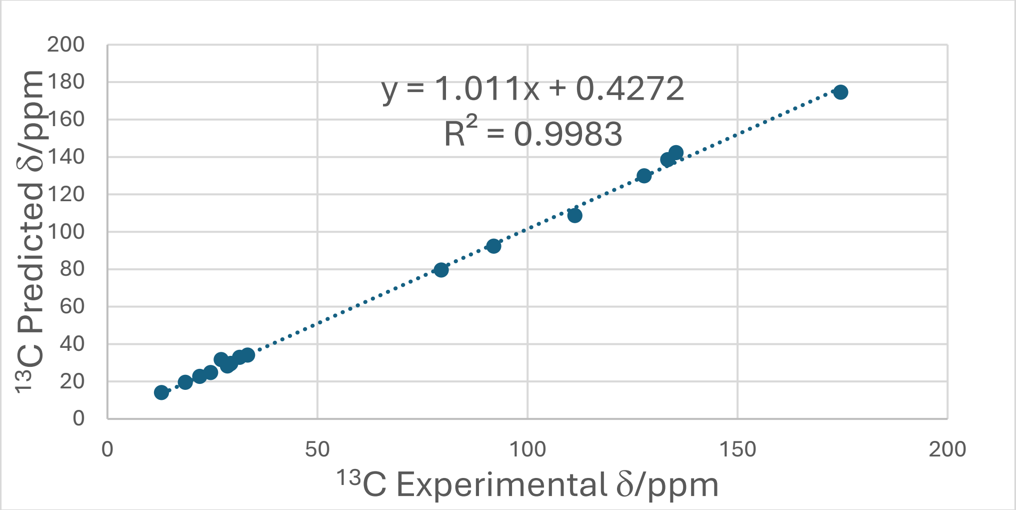

Figure S42. Experimental vs predicted chemical shifts for **2**. Predicted data calculated by ACD/Labs Structure Elucidator HOSE Code algorithm[3].

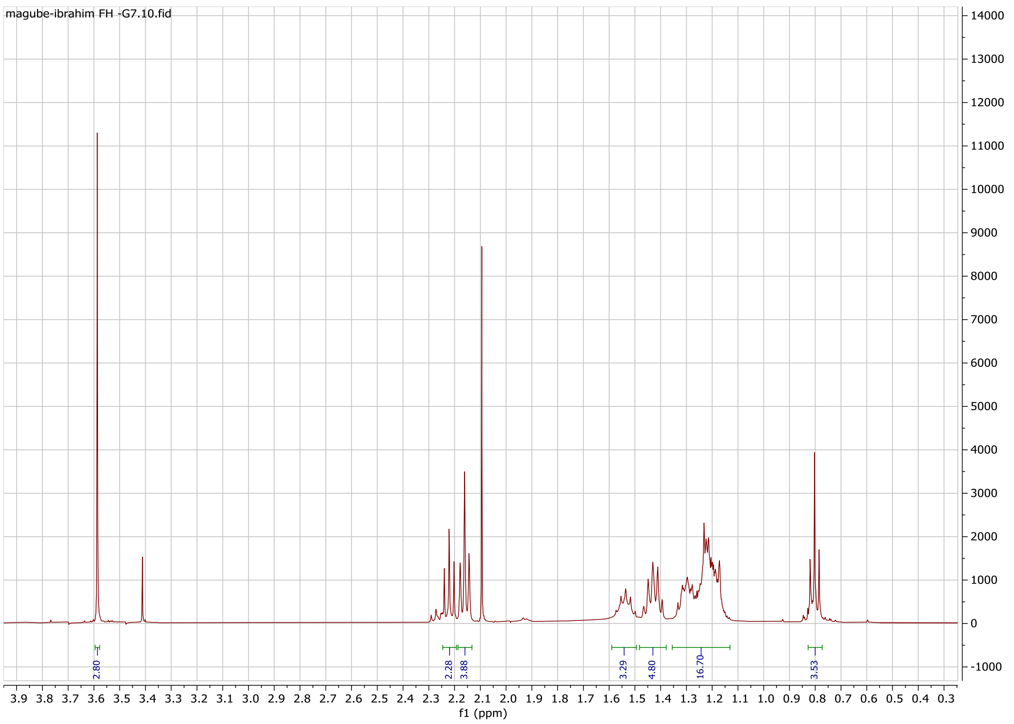

Figure S43. ^{1}H NMR spectrum for compound **3** in $CDCl_3$ at 400 MHz.

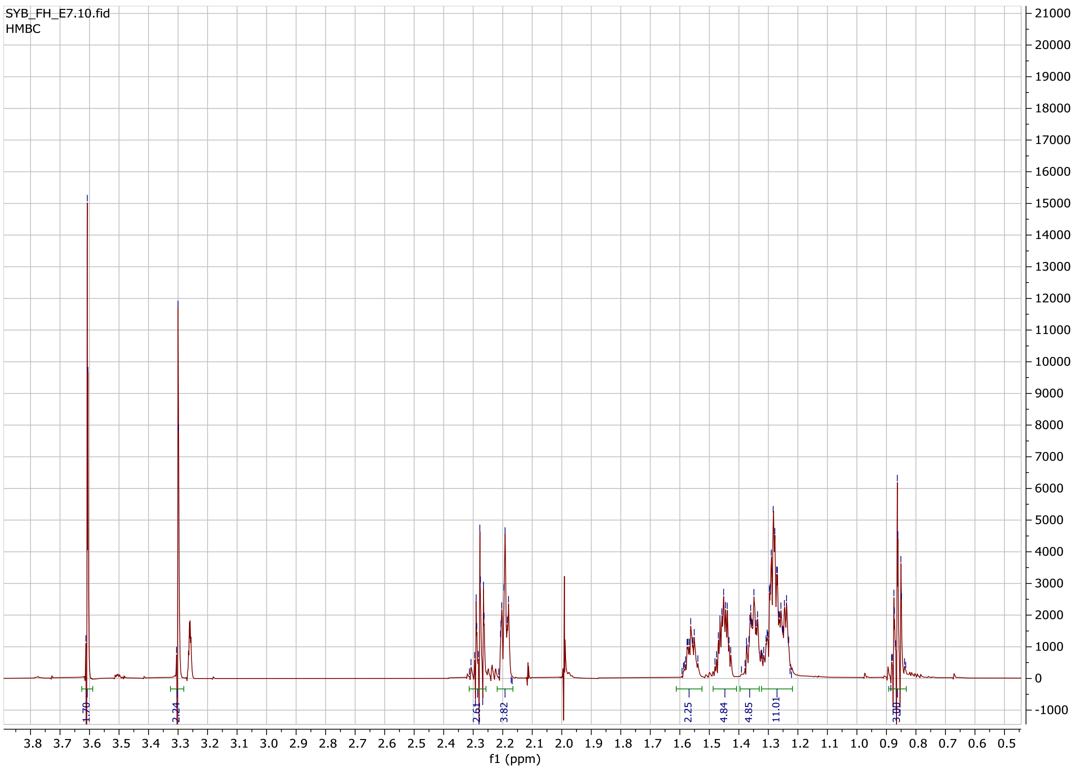

Figure S44. ^{1}HNMR (water suppression) spectrum for compound **3** in CD_3OD at 600 MHz.

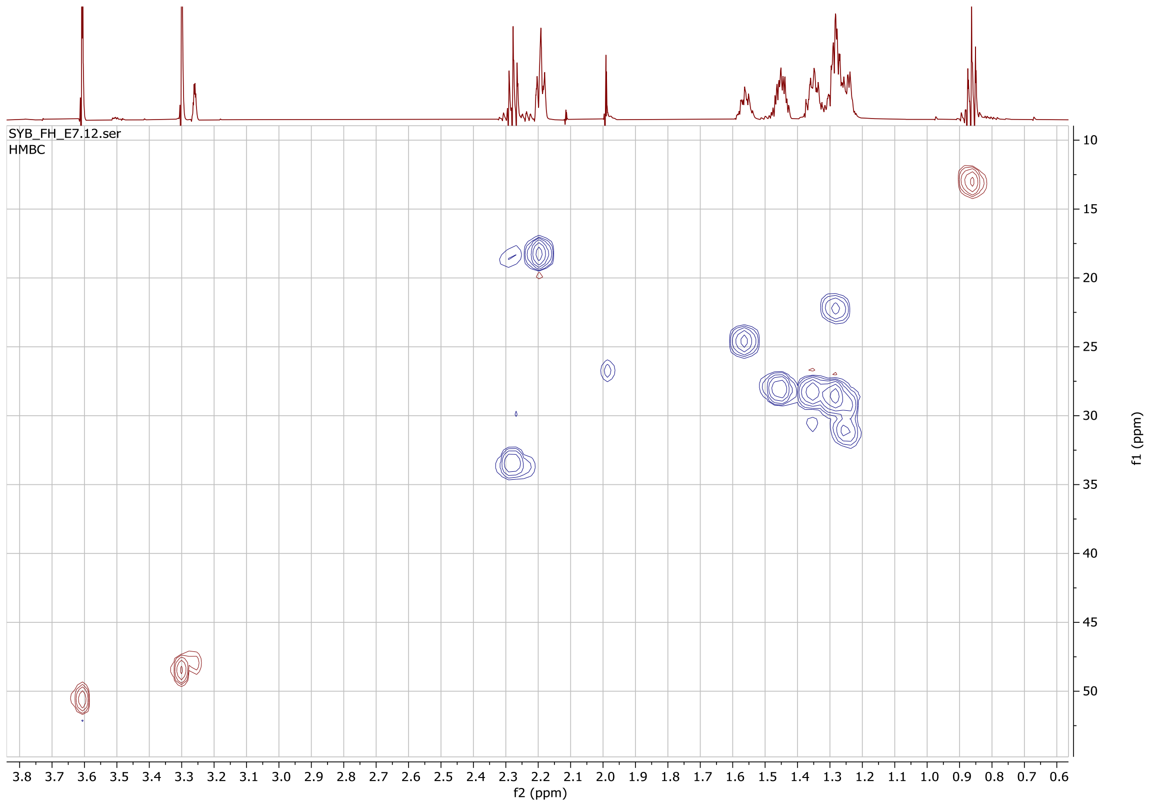

Figure S45. Edited-HSQC spectrum for compound **3** in CD_3OD at 600 MHz.

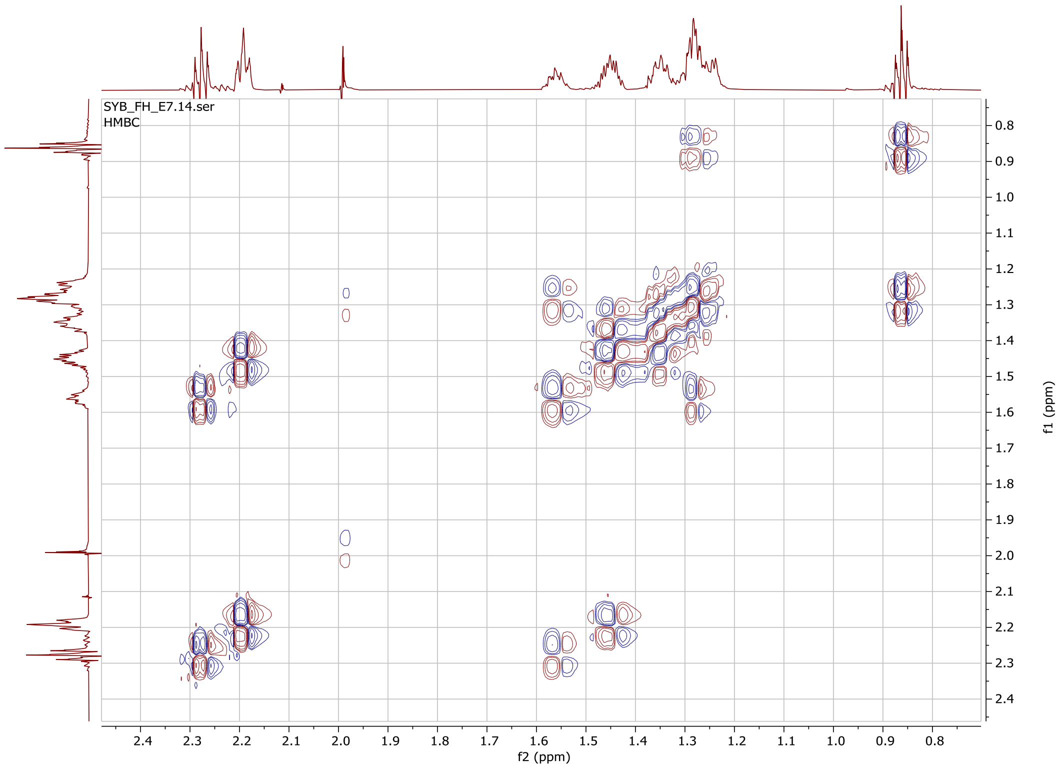

Figure S46. DQF-COSY spectrum for compound **3** in CD_3OD at 600 MHz.

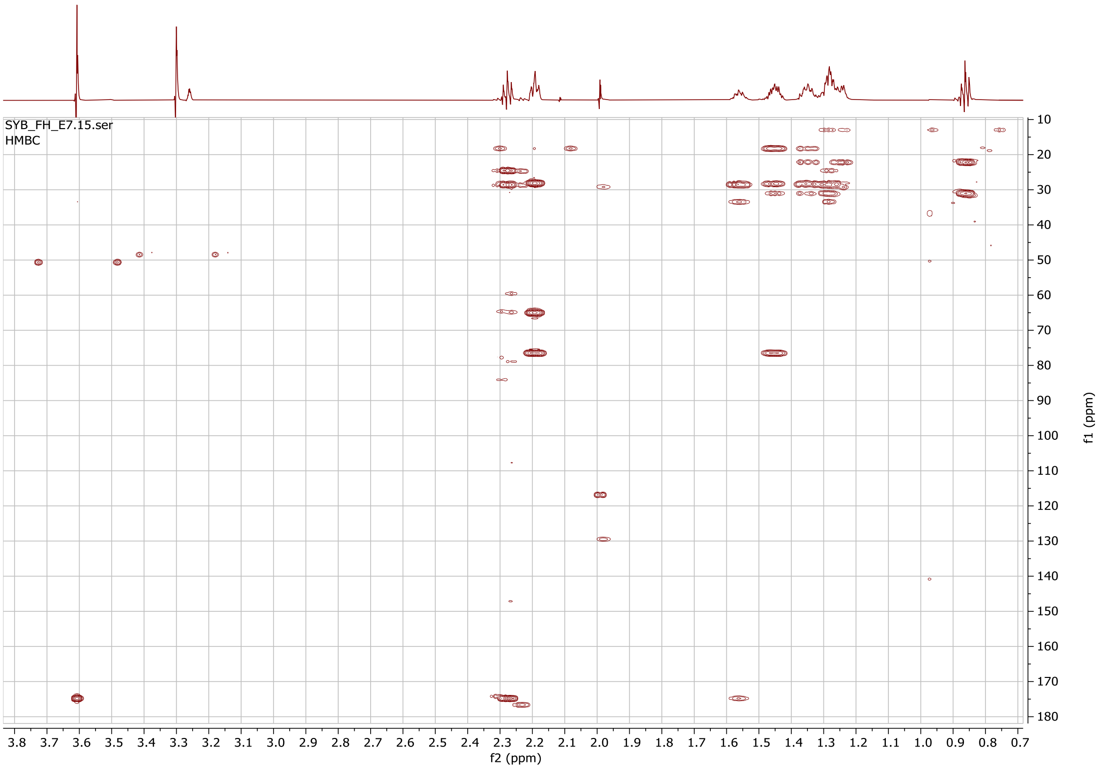

Figure S47. HMBC spectrum for compound **3** in CD_3OD at 600 MHz.

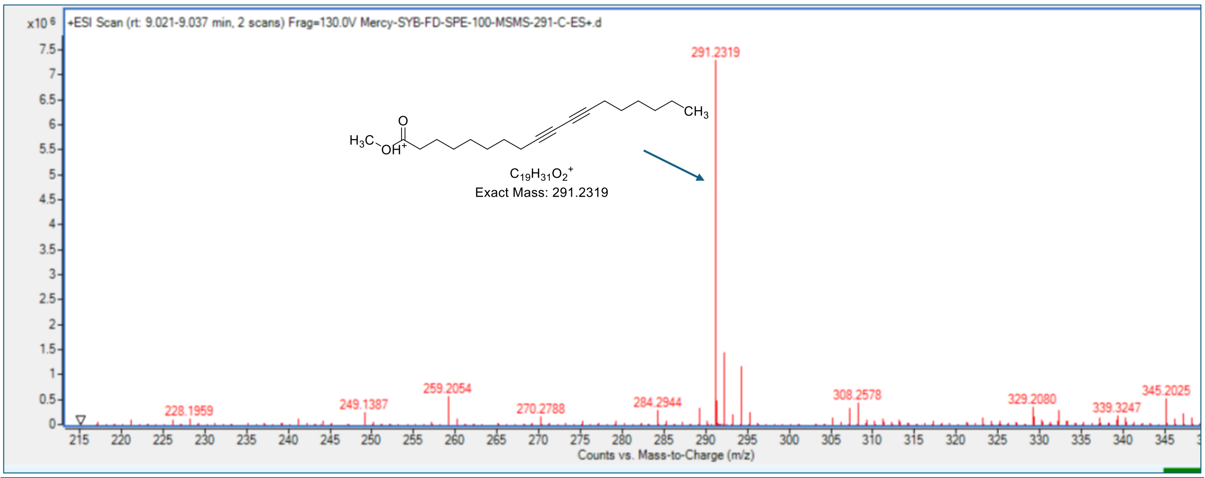

Formula (M)	Ion Formula	Mass (MFG)	m/z (Calc)	Diff (ppm)	DBE
C19 H30 O2	C19 H31 O2	290.2246	291.2319	0.49	5

Figure S48. HR-ESIMS (ES^+) spectrum and molecular formula generated by Agilent Mass Hunter[1] for **3**.

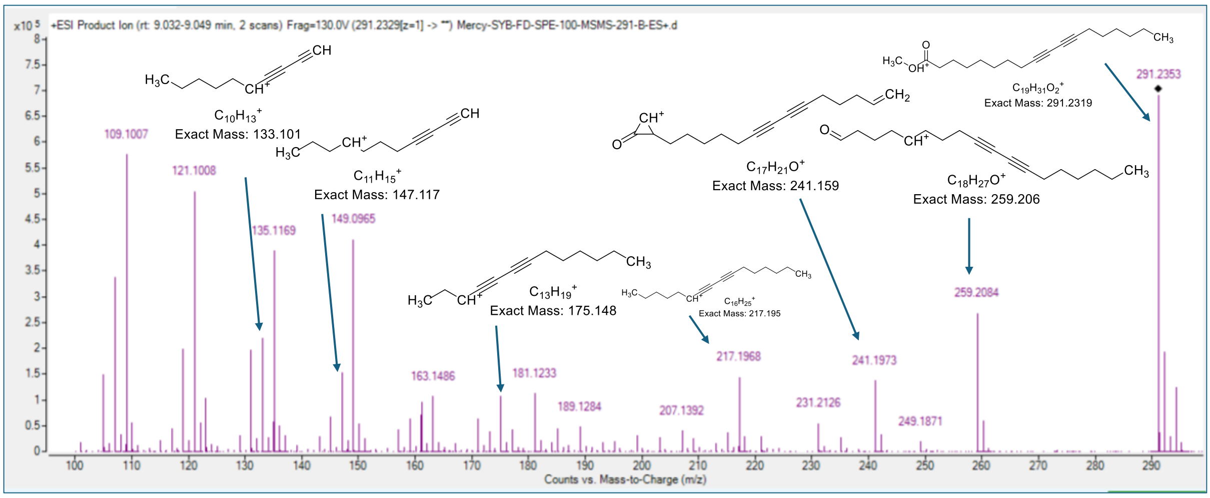

Figure S49. HR-ESIMS-MS/MS spectrum for **3** and fragment assignment using ACD/Labs MS Fragmenter[2].

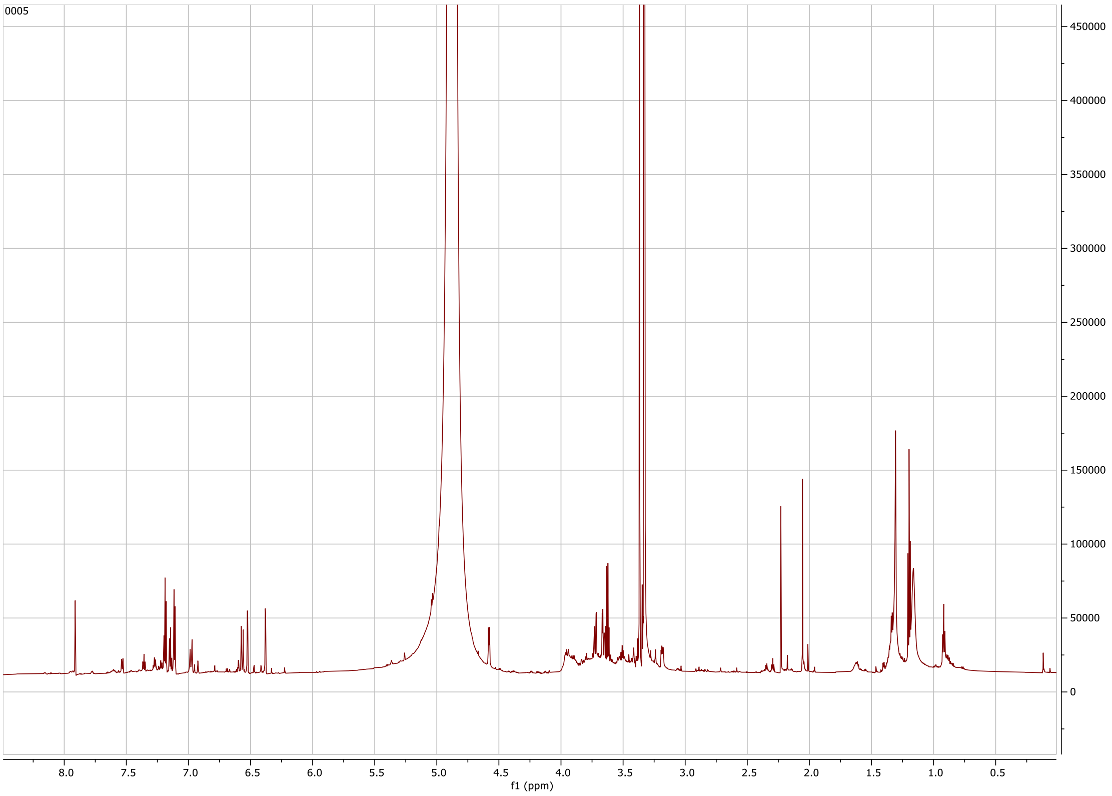

Figure S50. ^1H NMR for compound 5 in CD_3OD at 800 MHz.

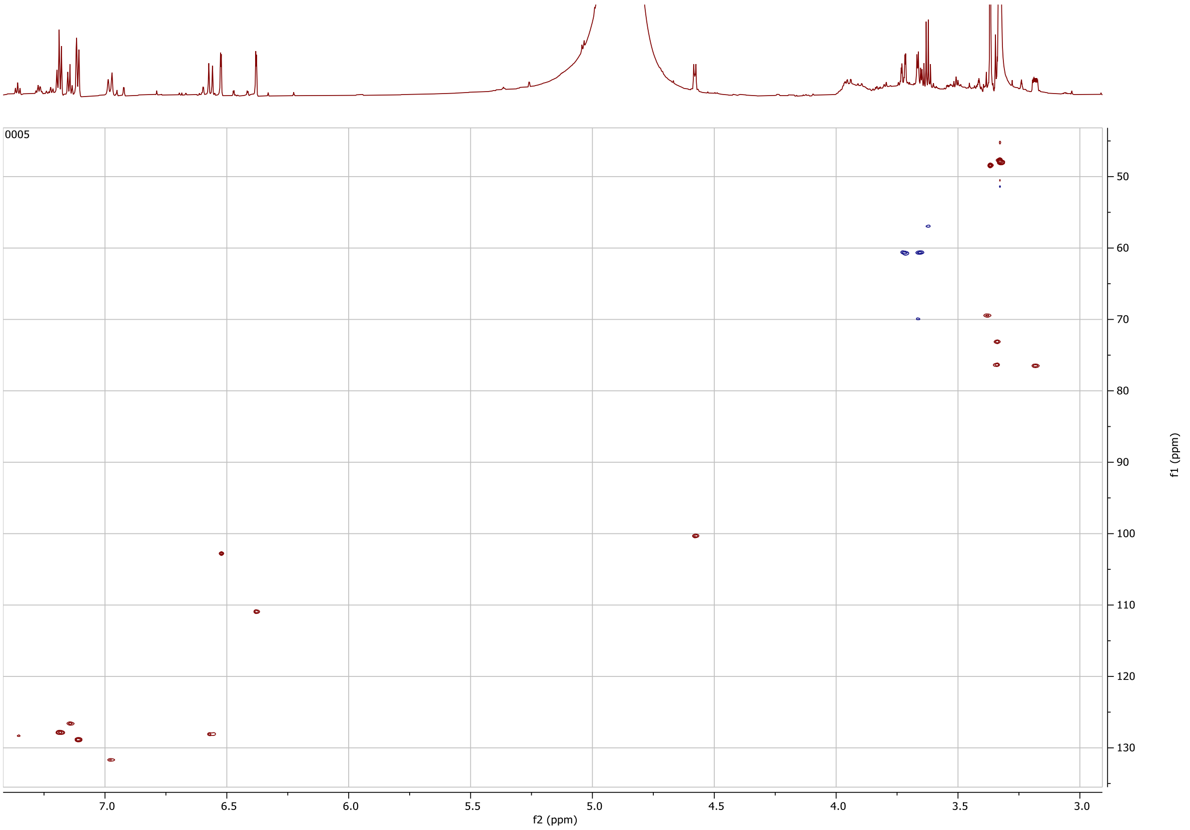

Figure S51. Edited-HSQC spectrum for compound 5 in CD_3OD at 800 MHz.

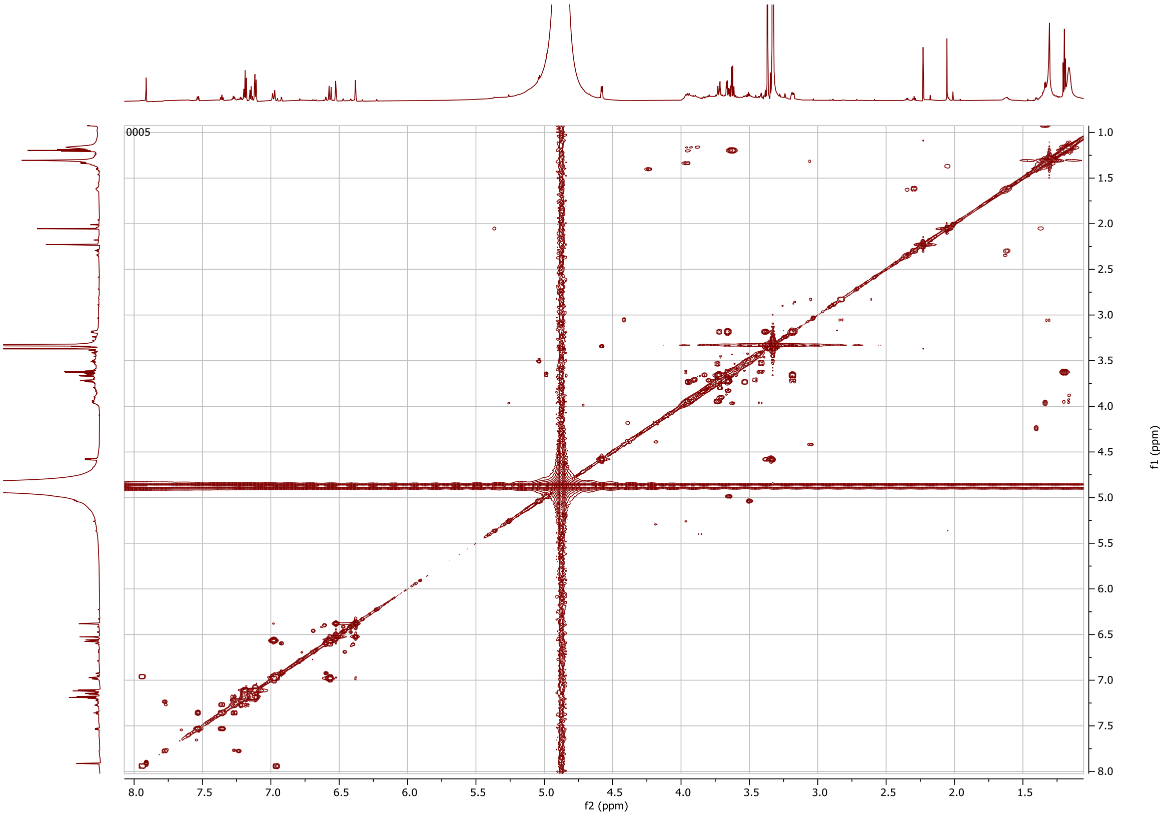

Figure S52. COSY spectrum for compound 5 in CD_3OD at 800 MHz.

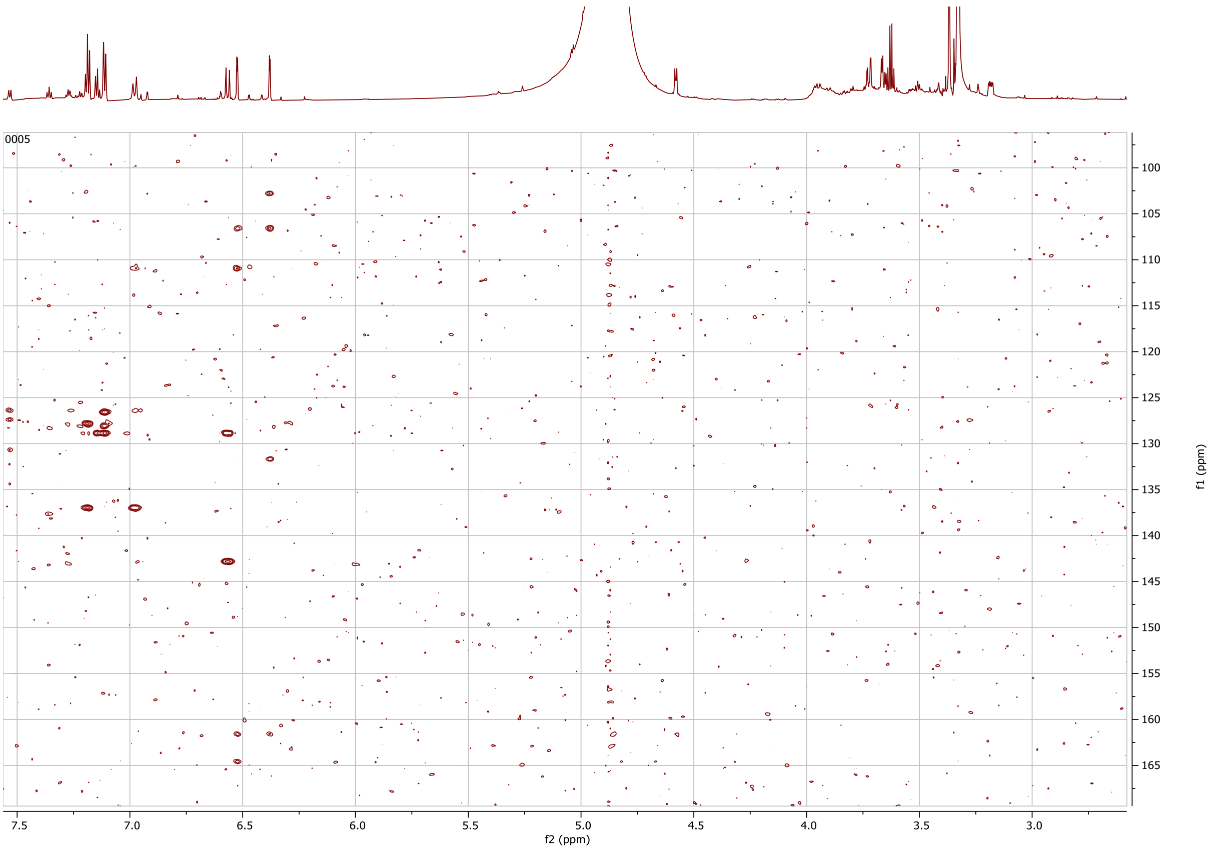

Figure S53. HMBC spectrum for compound 5 in CD3OD at 800 MHz.

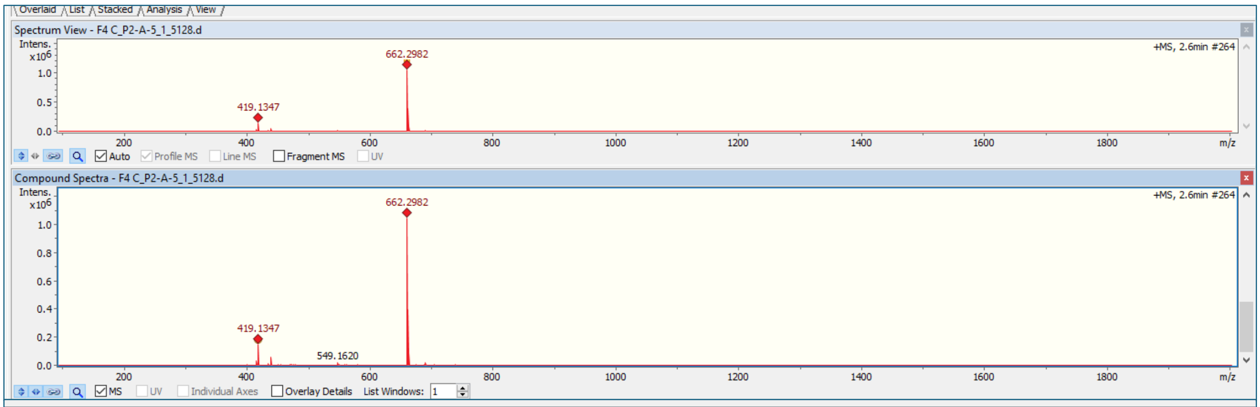

Meas. m/z	#	Ion Formula	m/z	err [ppm]	mSigma	# mSigma	Score	rdb	e⁻ Conf	N-Rule
419.1347	1	$C_{21}H_{23}O_9$	419.1337	-2.5	6.6	1	100.00	11.0	even	ok

Figure S54. HRESIMS (ES^+) spectrum for compound 5.

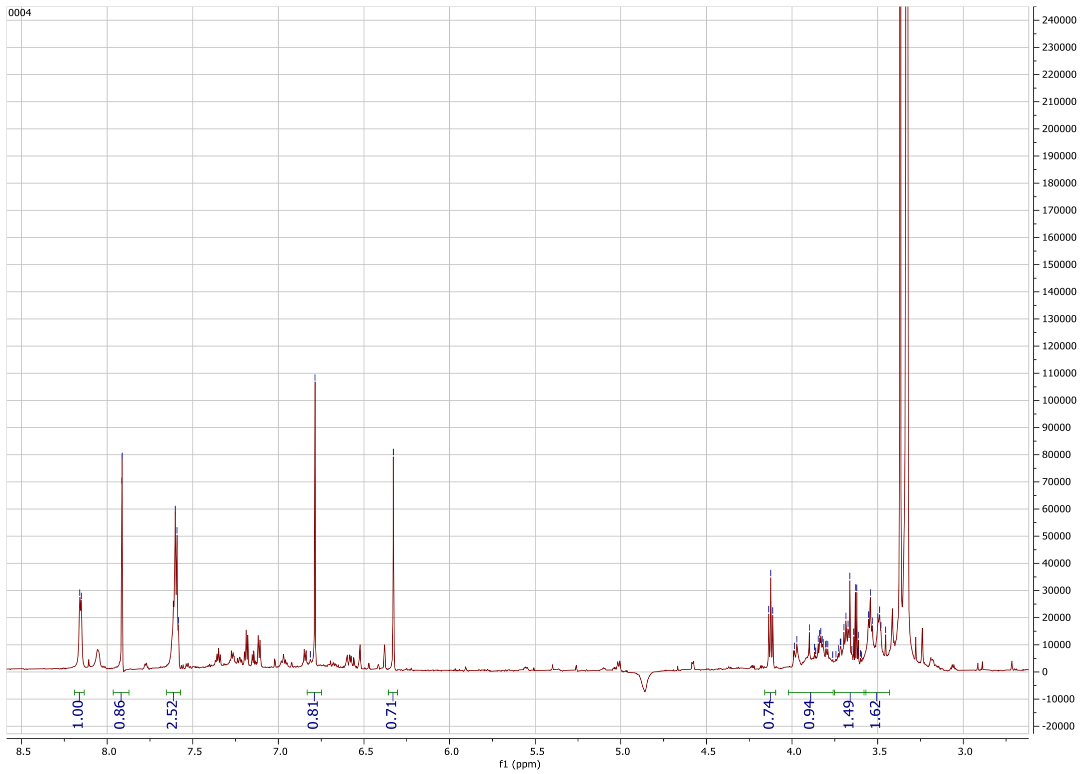

Figure S55. ^{1}H NMR for compound 6 in CD_3OD at 800 MHz.

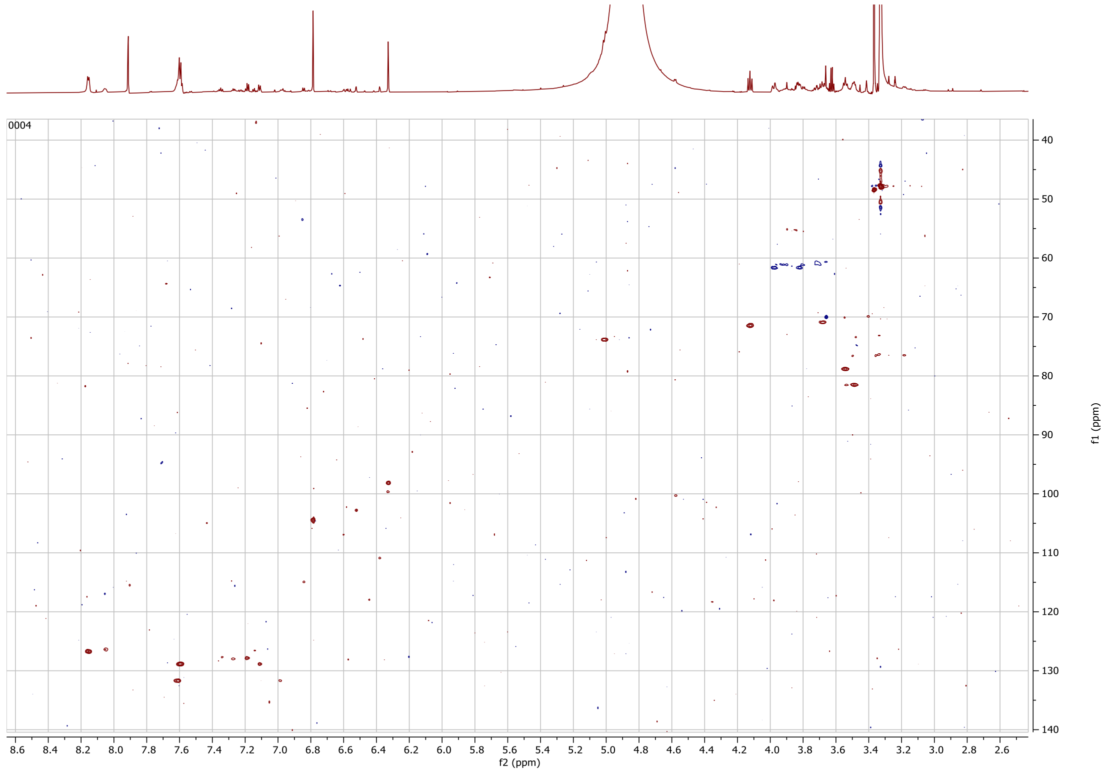

Figure S56. Edited-HSQC spectrum for compound 6 in CD_3OD at 800 MHz.

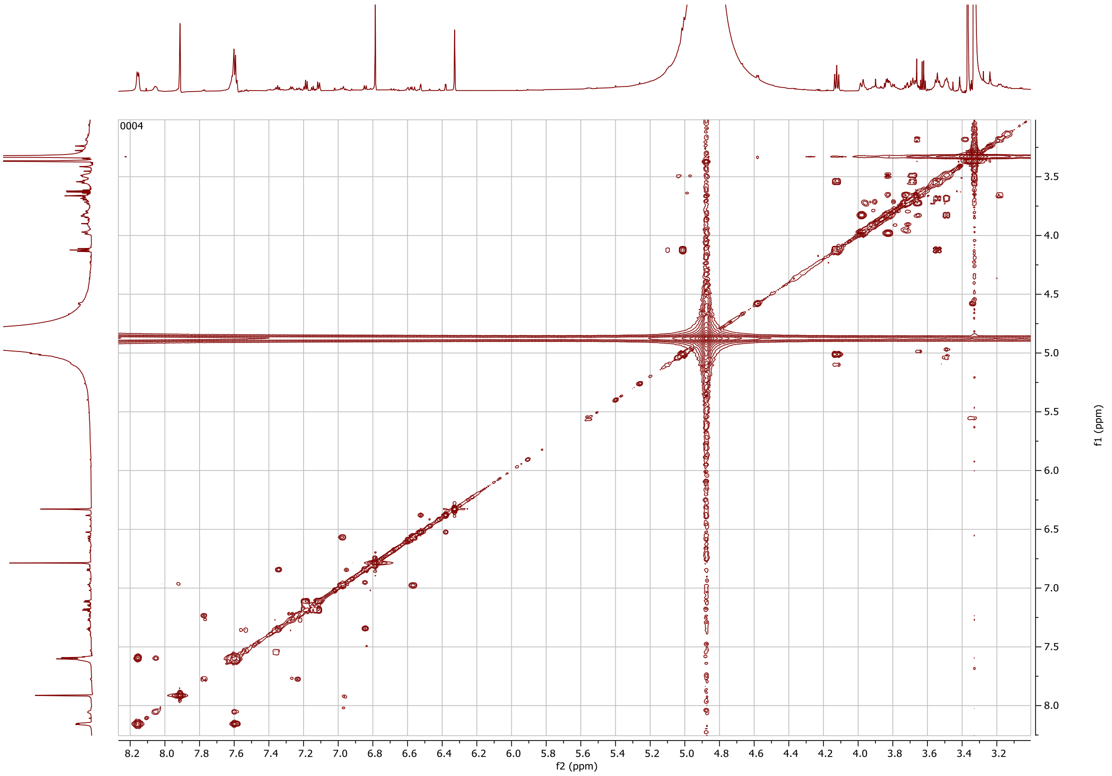

Figure S57. Edited-HSQC spectrum for compound 6 in CD_3OD at 800 MHz.

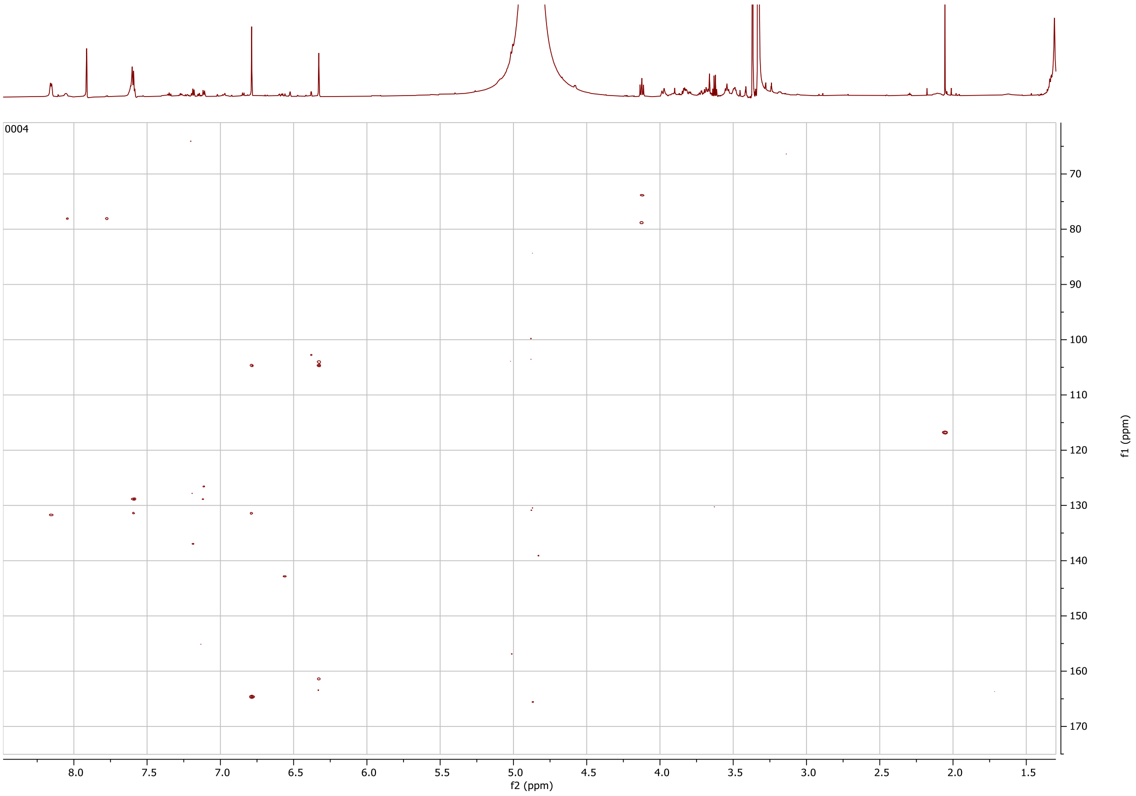

Figure S58. HMBC spectrum for compound 6 in CD_3OD at 800 MHz.

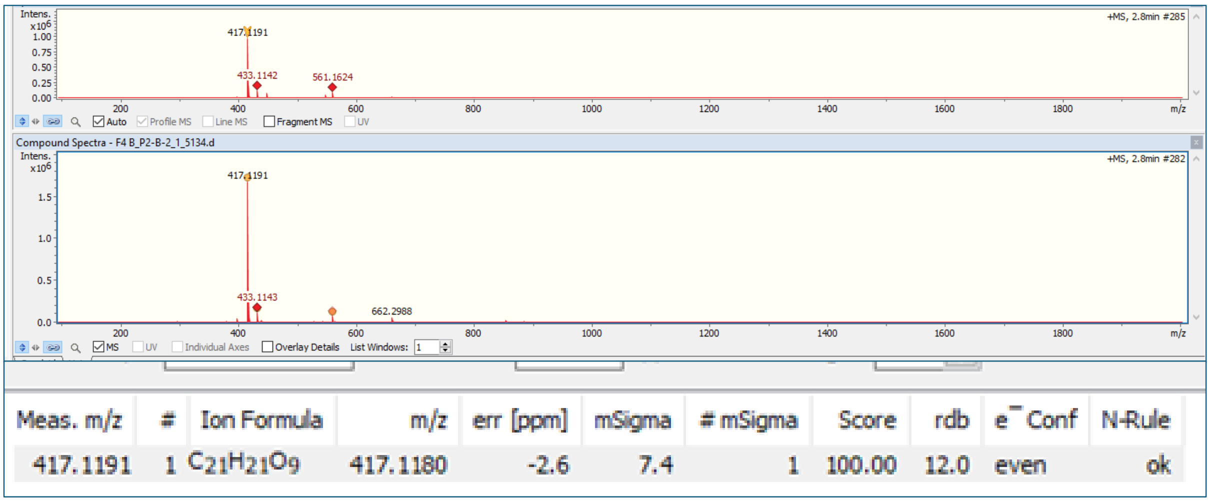

Meas. m/z	#	Ion Formula	m/z	err [ppm]	mSigma	# mSigma	Score	rdb	e⁻ Conf	N-Rule
417.1191	1	$C_{21}H_{21}O_9$	417.1180	-2.6	7.4	1	100.00	12.0	even	ok

Figure S59. HRESIMS (ES^+) spectrum for compound 6.

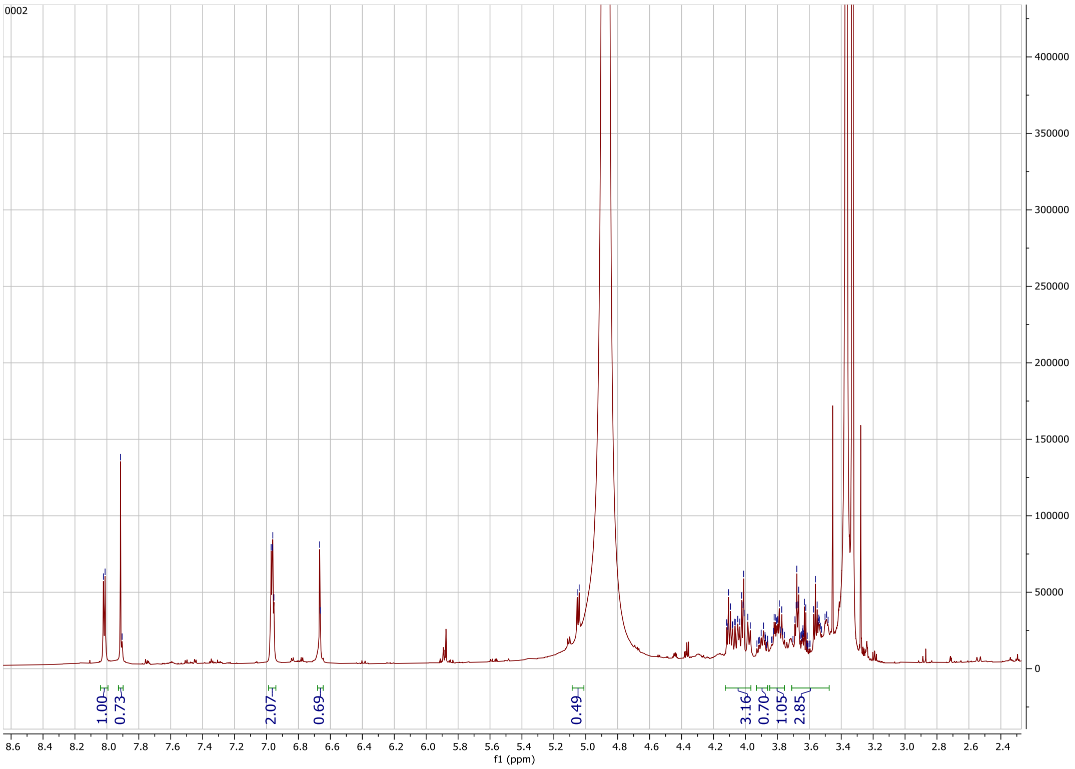

Figure S60. ^{1}H NMR for compound 7 in CD_3OD at 800 MHz.

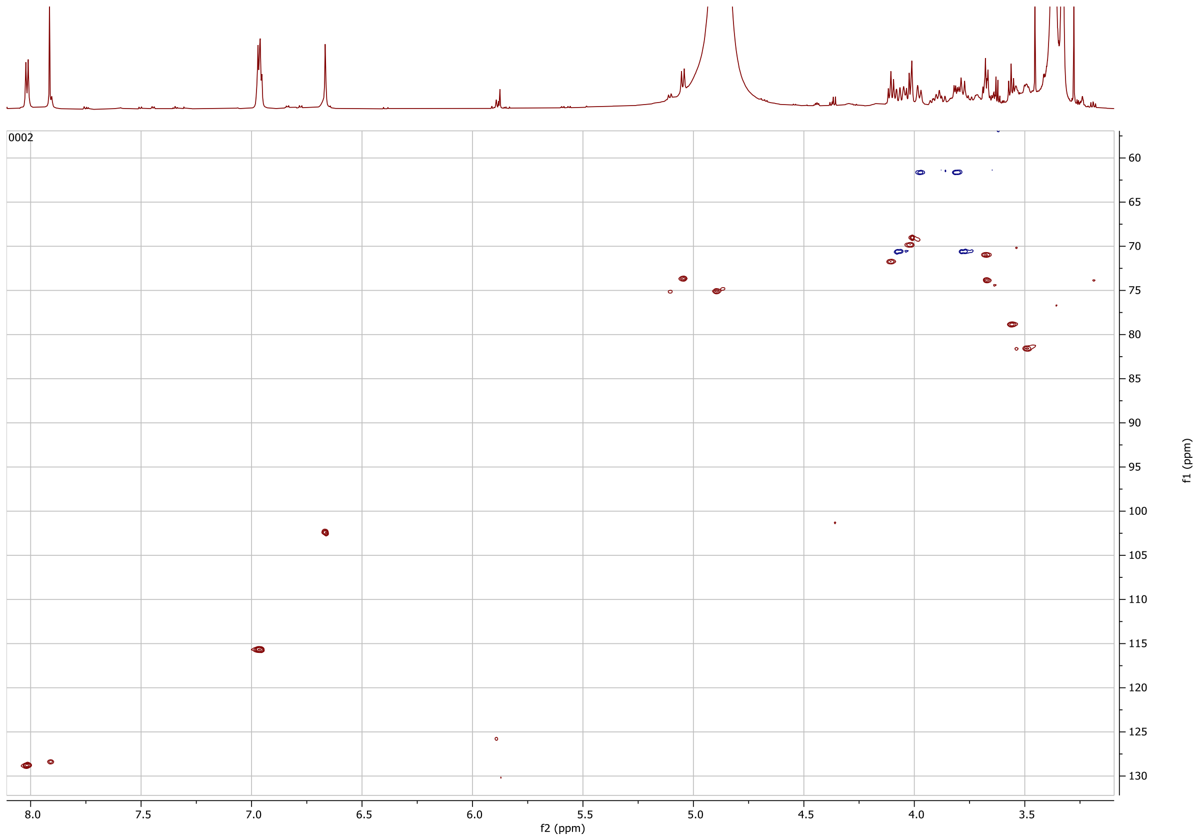

Figure S61. Edited-HSQC spectrum for compound 7 in CD_3OD at 800 MHz.

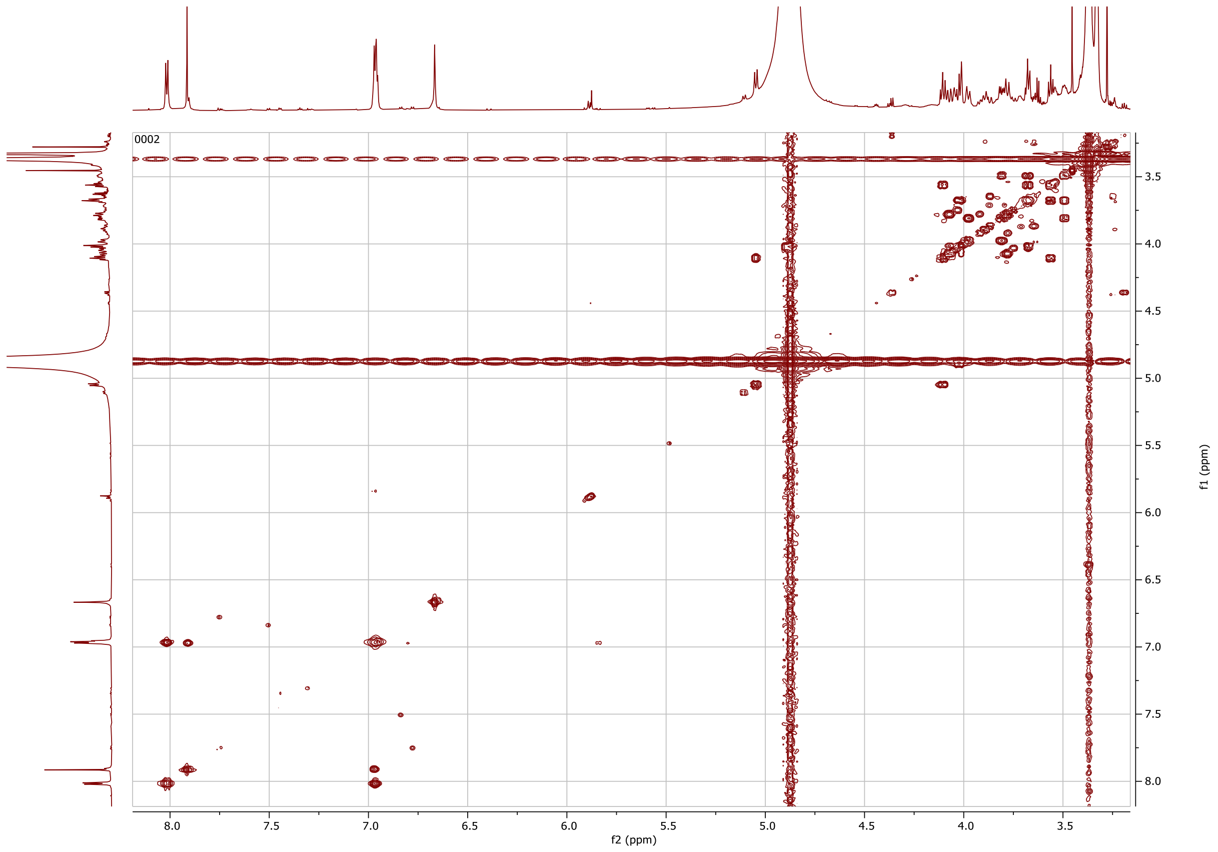

Figure S62. COSY spectrum for compound 7 in CD_3OD at 800 MHz.

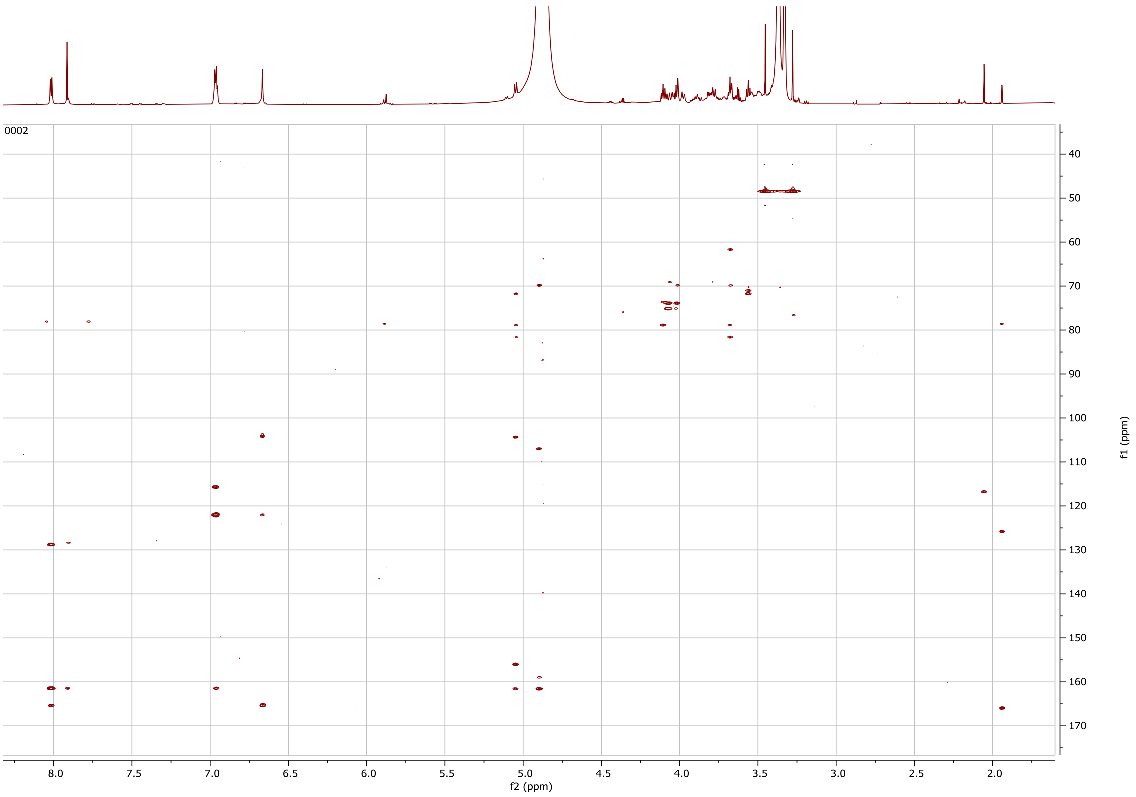

Figure S63. HMBC spectrum for compound 7 in CD_3OD at 800 MHz.

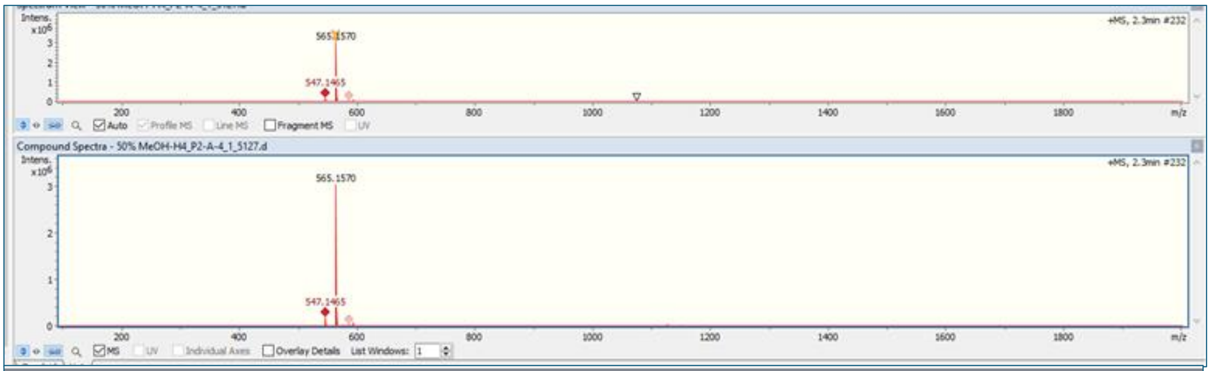

Meas. m/z	#	Ion Formula	m/z	err [ppm]	mSigma	# mSigma	Score	rdb
565.1570	2	$C_{44}H_{21}O$	565.1587	2.9	104.5	2	4.83	35.0
565.1570	1	$C_{26}H_{29}O_{14}$	565.1552	-3.3	8.6	1	100.00	13.0

Figure S64. HRESIMS (ES^+) spectrum for compound 7.

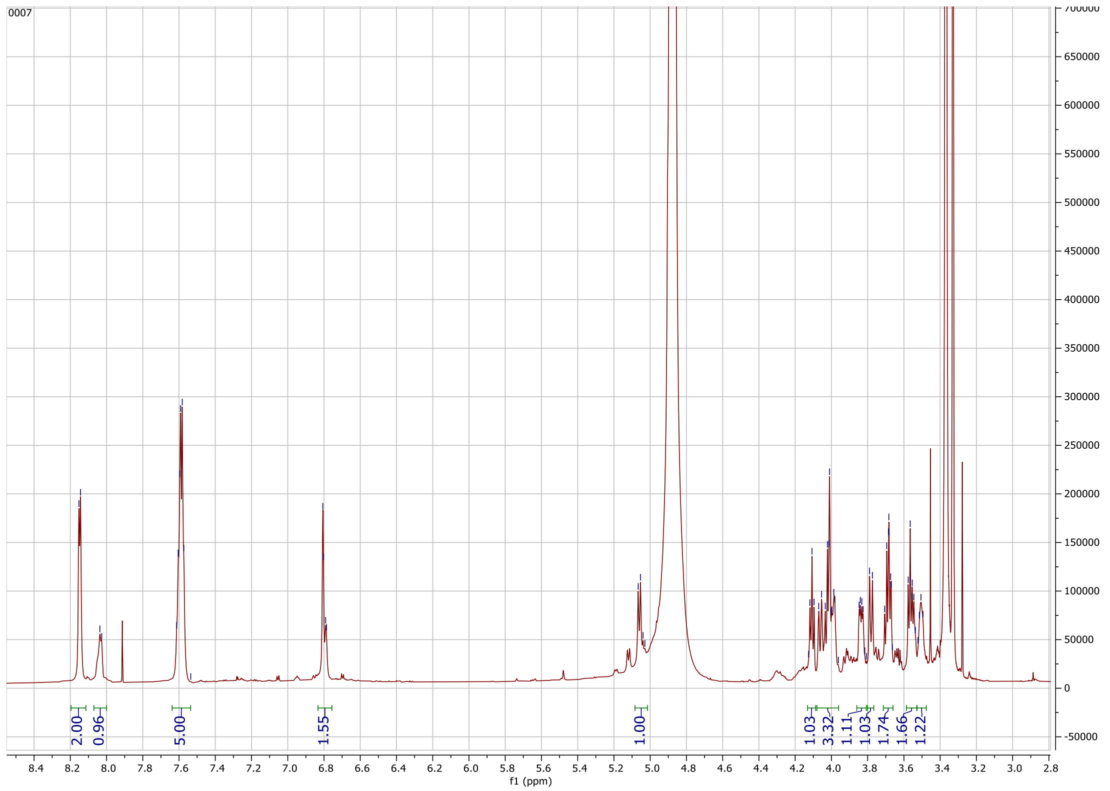

Figure S65. ^{1}H NMR for compound 8 in CD_3OD at 800 MHz.

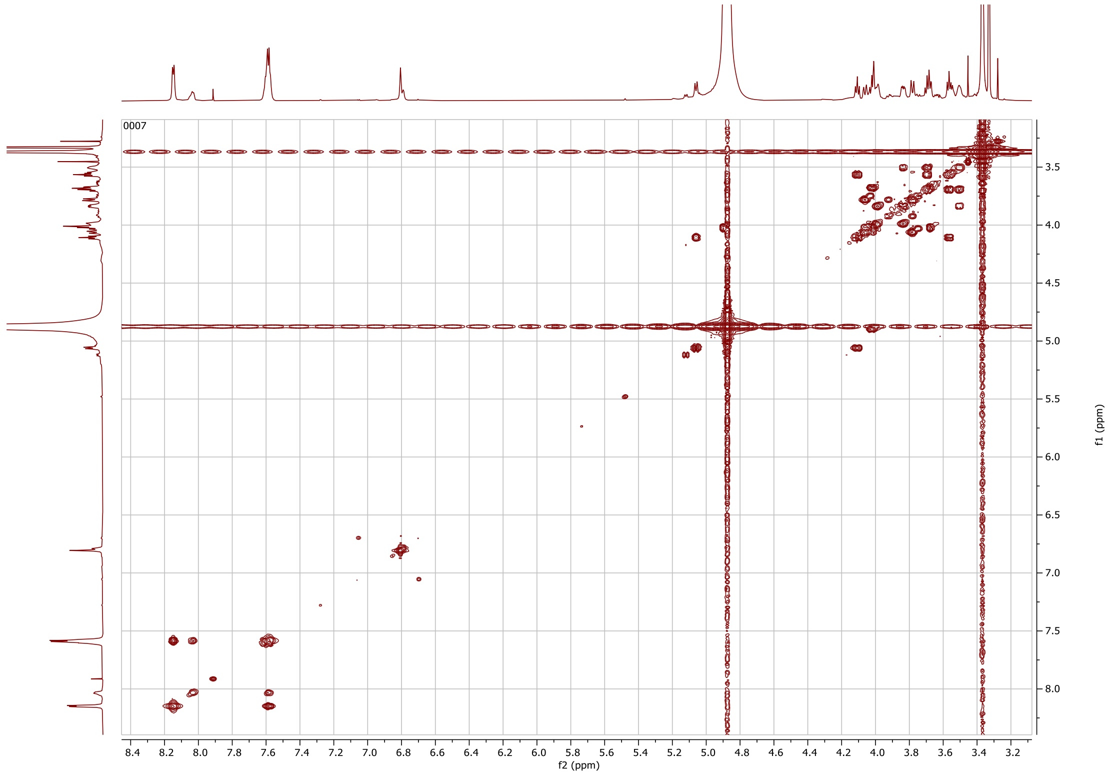

Figure S66. COSY spectrum for compound 8 in CD_3OD at 800 MHz.

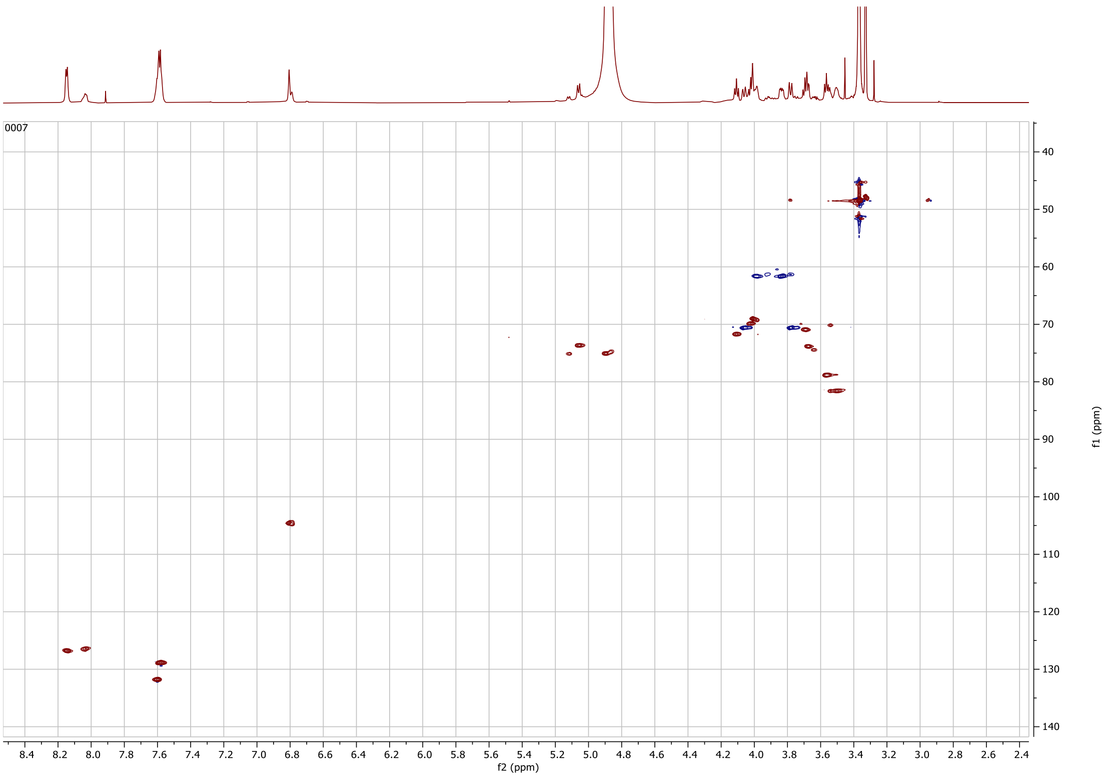

Figure S67. Edited-HSQC spectrum for compound 8 in CD3OD at 800 MHz.

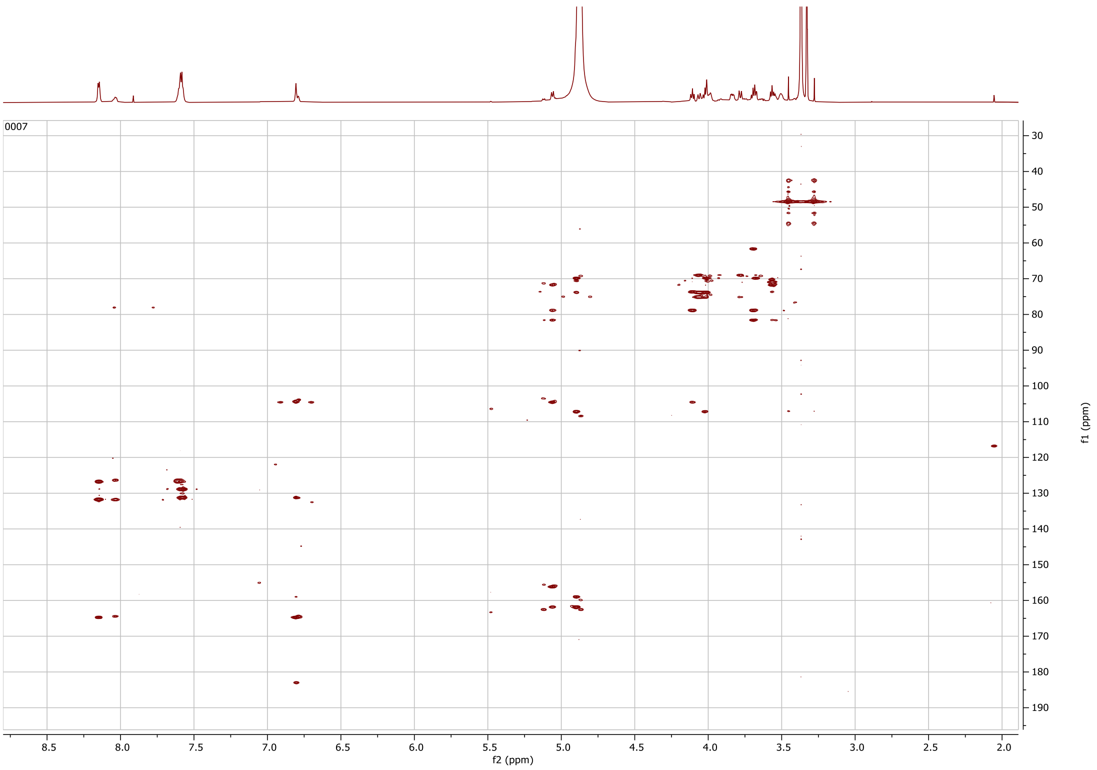

Figure S68. HMBC spectrum for compound 8 in CD3OD at 800 MHz.

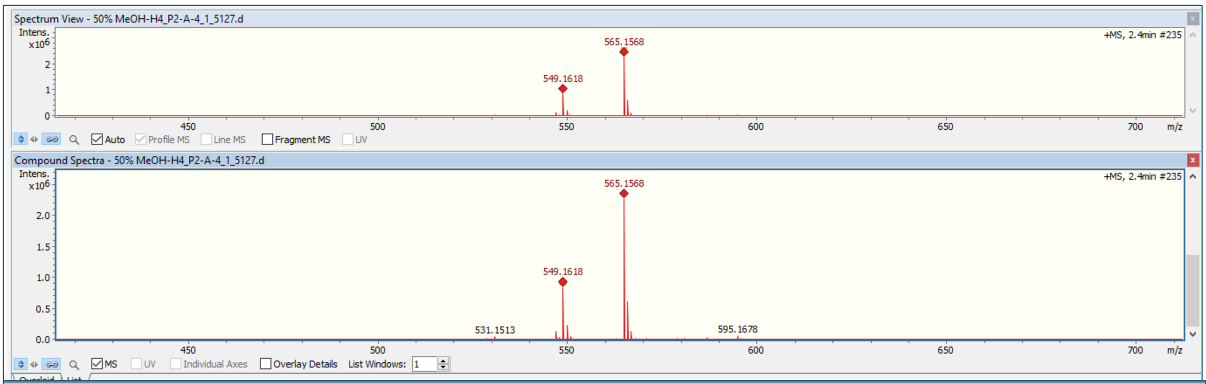

Meas. m/z	#	Ion Formula	m/z	err [ppm]	mSigma	# mSigma	Score	rdb
549.1618	1	$C_{26}H_{29}O_{13}$	549.1603	-2.9	8.6	1	100.00	13.0
549.1618	2	$C_{44}H_{21}$	549.1638	3.5	105.6	2	3.08	35.0

Figure S69. HRESIMS (ES$^+$) spectrum for compound 8.

	Gram-negative pathogen								Gram-positive pathogen					Yeast pathogen		Fungal pathogen	
Sandalwood fractions		E. coli ATCC 25922		K. pneumoniae ATCC700603		P. aeruginosa PAO-1		A. baumannii MB5973		MSSA ATCC29213		MRSA MB5393		C. albicans ATCC64124		A. fumigatus ATCC46645	
EXT_EXTERNAL_ID	Conc. [ug/mL]	% INH_1:	% INH_2:	% INH_1:	% INH_2:	% INH_1:	% INH_2:	% INH_1:	% INH_2:	% INH_1:	% INH_2:	% INH_1:	% INH_2:	% INH_1:	% INH_2:	% INH_1:	% INH_2:
SyB-FH	64	-22	-28	-11	3	7	-12	47	36	-91	-93	1	-7	-29	0	-37	-24
ShB-FH	64	0	-21	20	10	26	6	64	46	-97	-87	-1	-12	-3	-4	-29	-29
SaB-FH	32	-15.5	-21.1	-24.2	-10.6	15.8	2.5	76.6	45.4	-60.8	11.7	3.1	-16.7	-9.4	13.2	-26.4	-18.89269
SyB-FD	32	-22.0	-19.4	-28.9	-11.9	9.7	-0.1	49.8	31.0	9.5	9.0	0.8	-3.5	1.7	16.0	-1.6	-6
ShB-FD	32	-16.0	-17.8	-26.5	-20.3	3.6	-11.0	52.6	44.5	4.4	9.0	0.3	-3.2	-4.7	2.8	2.2	-12
SaB-FD	32	-10.3	-22.8	-27.1	-14.7	14.7	-1.1	57.8	43.8	-3.2	16.6	5.9	0.0	2.6	19.4	-10.2	-10
ShB-FM	64	16.8	-13.6	12.8	18.4	26.7	2.3	6.4	-2.3	0.0	4.6	-5.6	-8.2	-0.9	-10.1	1.2	-5
SaB-FM	32	-14.0	-19.0	-15.0	8.4	24.6	-8.1	71.7	46.0	-7.0	8.4	2.2	-4.7	-4.3	-11.8	1.3	-8
SaL-FM	64	-20.2	-28.0	-25.5	-8.1	36.4	-6.4	78.0	40.7	-22.8	-28.6	2.0	-3.2	-11.1	10.1	-40.6	-25
SyB-WB	64	-18.6	-23.2	-28.7	-20.6	-7.6	-18.3	-18.2	-30.1	-12.0	0.8	-2.0	-8.5	-66.2	-71.5	-1.6	-8
ShB-WB	32	22.0	-11.1	1.1	15.0	23.8	1.6	-12.0	-0.8	-4.4	3.5	-4.8	-8.2	9.0	-6.9	-1.3	-6
SaB-WB	32	-7.4	-16.5	-24.6	-12.2	4.2	-21.7	2.3	-11.7	-16.5	7.4	0.3	-6.5	0.9	-15.3	-5.8	-5
SyL-FH	32	-3.8	-20.7	-28.3	-17.5	26.9	6.0	94.3	61.9	5.1	11.7	-5.9	-7.6	34.2	29.5	-1.0	-3
ShL-FH	32	-1.4	-11.5	-14.8	7.8	23.6	3.0	74.8	51.6	8.9	10.1	-1.4	-6.2	12.0	28.1	-3.0	-6
SaL-FH	32	14.0	-2.8	0.5	13.8	28.6	16.9	94.3	78.5	-16.5	-23.2	-3.4	-2.6	47.0	25.3	5.0	-7
SaL-FD	32	-16.6	-21.1	-22.2	-8.7	16.6	-10.8	48.4	36.3	15.2	21.0	-1.4	-4.4	-17.5	2.1	-2.1	-9
SaL-WB	64	20.7	-13.4	-22.0	-14.4	27.8	15.2	77.6	50.7	13.9	11.2	-3.4	-5.3	28.6	35.8	-6.5	-16
ShL-WB	64	-8.2	-22.1	-22.8	-12.8	30.9	0.9	83.9	50.1	13.3	15.0	-2.5	-7.0	17.1	20.8	-14.5	-15

Table S1. Sandalwood anti-microbial screening results. SyB (*Santalum yasi* bark), ShB (*Santalum yasi-album* hybrid bark), SaB (*Santalum album* bark), SyL (*Santalum yasi* leaf), ShL (*Santalum yasi-album* hybrid leaf), SaL (*Santalum album* leaf). FH (hexane fraction), FD (dichloromethane fraction), FM (methanol-water fraction), WB (water butanol fraction). Highlighted in green are the active at >50% inhibition.

			A549	A2058	HepG2	HT29	MCF-7	MiaPaca-2	PC3
Sandalwood fraction	Compound ID	Conc. MG/ML	Activity	Activity	Activity	Activity	Activity	Activity	Activity
	DMSO		-1	0	0	0	-1	0	0
	MMS		-100	-100	-100	-100	-100	-99	-100
	DMSO		-0.665357	-0.209213	-0.403714	0.0385836	-0.67237	0.142682597	-0.31111
SyB-FH	Ext-ED37765-a01	0.1	-40	-76	-90	-39	-44	-52	-53
ShB-FH	Ext-ED37768-a01	0.1	-66	-60	-71	-59	-68	-2	-45
SaB-FH	Ext-ED37746-a01	0.05	-45	-52	-43	-55	-31	3	-41
SyB-FD	Ext-ED37755-a01	0.05	-8	2	4	6	15	2	-6
ShB-FD	Ext-ED37749-a01	0.05	-25	-17	-6	-3	-15	1	-21
SaB-FD	Ext-ED37748-a01	0.05	-31	-24	-7	-19	-2	-4	-27
SaB-FM	Ext-ED37756-a01	0.05	-24	1	16	-3	20	-2	-20
SaB-WB	Ext-ED37747-a01	0.05	-12	-11	-5	-3	16	3	-20
ShB-FM	Ext-ED37761-a01	0.1	-16	5	-17	0	-1	2	-15
SyB-WB	Ext-ED37758-a01	0.1	-13	2	24	3	26	0	-9
ShB-WB	Ext-ED37753-a01	0.05	-6	5	9	-3	3	2	-15
ShL-FH	Ext-ED37754-a01	0.05	-9	5	13	1	-3	2	-17
SaL-FH	Ext-ED37752-a01	0.05	-39	-4	-56	-29	-48	-7	-47
SaL-FD	Ext-ED37751-a01	0.05	-12	-14	13	-4	-31	-1	-8
SyL-FH	Ext-ED37745-a01	0.05	-28	-20	-18	-22	-48	3	-32
ShL-WB	Ext-ED37762-a01	0.1	-29	2	-34	-7	19	0	-21
SaL-FM	Ext-ED37764-a01	0.1	-42	-77	-88	-42	-60	-60	-72
SyL-FM	Ext-ED37744-a01	0.05	-45	-40	-39	-22	-45	0	-62
SaL-WB	Ext-ED37760-a01	0.1	-13	1	-19	3	-5	0	-21

Table S2. Cytotoxicity results of sandalwood fractions. A549 (Human lung carcinoma), A2058 (Human Caucasian metastatic melanoma), HepG2 (human liver carcinoma cells), MCF7 (human breast adenocarcinoma), MiaPaca-2 (Human Caucasian pancreatic carcinoma), and PC-3 (Human Caucasian prostate adenocarcinoma), SyB (*Santalum yasi* bark), ShB (*Santalum yasi-album hybrid* bark), SaB (*Santalum album* bark), SyL (*Santalum yasi* leaf), ShL (*Santalum yasi-album hybrid* leaf), SaL (*Santalum album* leaf). FH (hexane fraction), FD (dichloromethane fraction), FM (methanol-water fraction), WB (water butanol fraction).

Extraction Scheme

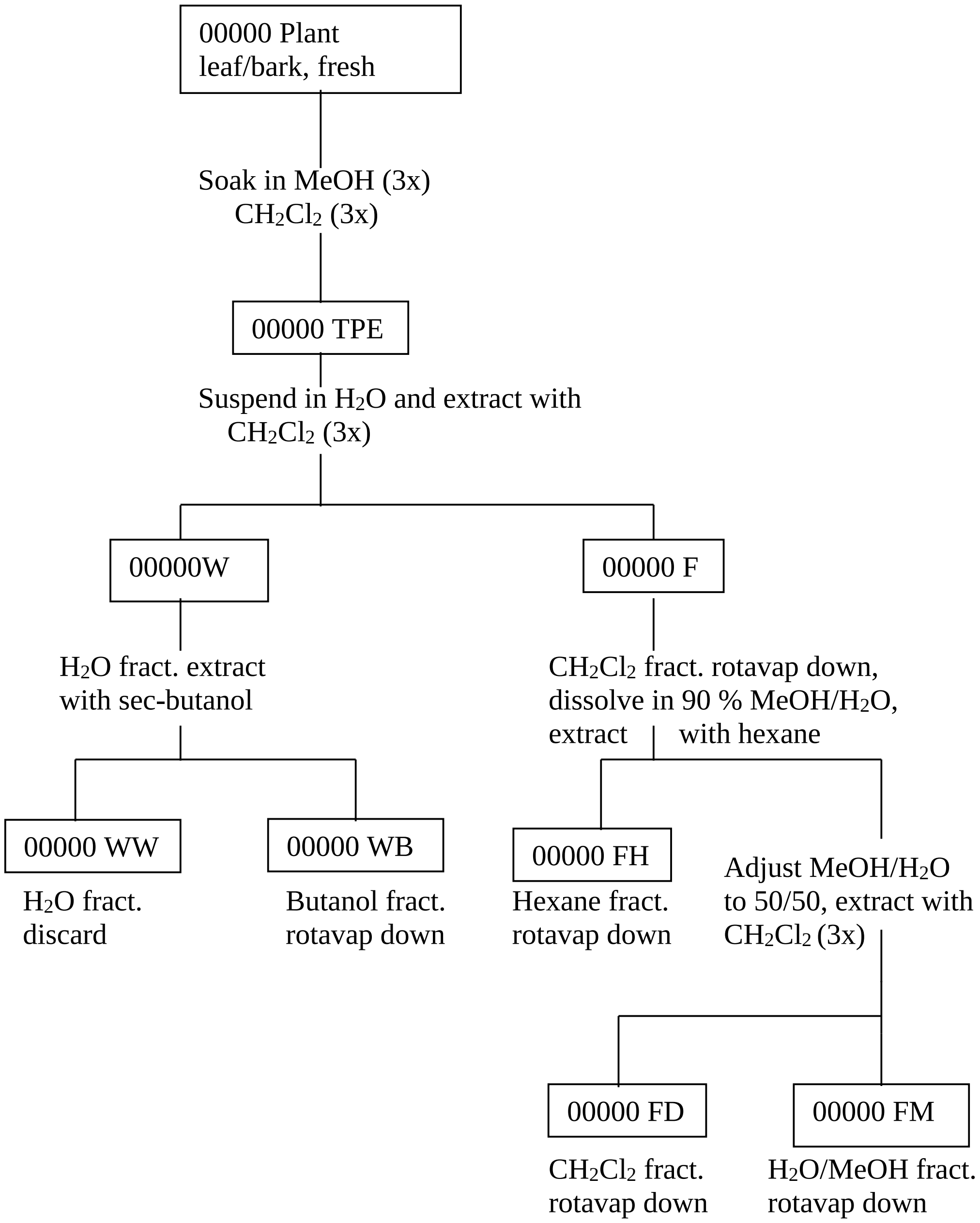

Figure S70. General extraction and partitioning scheme

For IC_{50}	Conc ug/mL	Surv1		Surv2		Surv3	
Compd. 1	1.56	0.4616	91	0.4386	84	0.4811	96
Compd. 1	3.13	0.4809	96	0.4923	99	0.5204	107
Compd. 1	6.25	0.4822	96	0.4852	97	0.5157	106
Compd. 1	12.5	0.5163	106	0.5157	106	0.5449	114
Compd. 1	25	0.5155	105	0.4979	101	0.5196	107
Compd. 1	50	0.4468	87	0.4468	87	0.4421	85
Compd. 1	100	0.2882	43	0.2912	44	0.2747	40
Compd. 1	200	0.1776	13	0.1751	12	0.1669	10
IC_{50}	91.19						
R square	0.9638						

Table S3. Determination of IC_{50} for compound **1** at various concentrations.

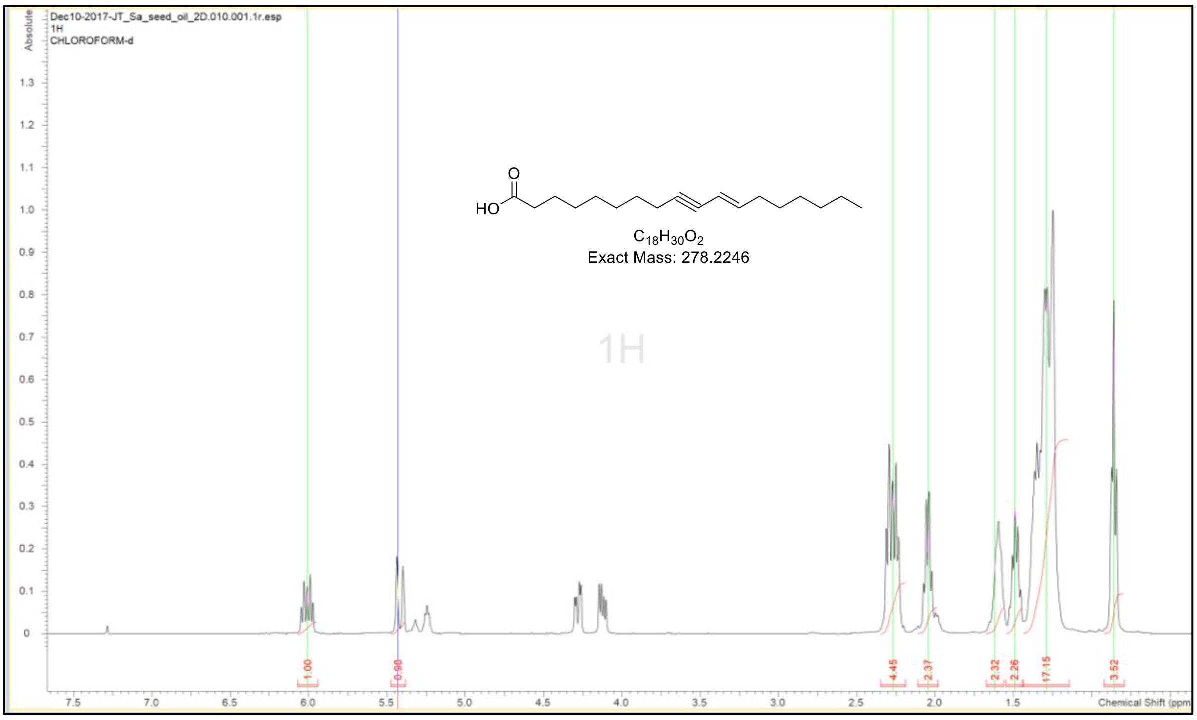

Figure S71. ^{1}H NMR spectrum of ximenynic acid (isolated from *Santalum album* seed) in $CDCl_3$ at 600 MHz. Seed oil was extracted with both methanol and dichloromethane, the same solvents used in the extraction bark and leaves of sandalwood plants. However, methyl ximenynate was not detected by ^{1}H NMR indicating that the conversion of acid to ester as an artifact of extraction did not take place during the extraction and purification process.

Reference

(1) *Robust Mass Spectrometry Application Software, MassHunter | Agilent*. https://www.agilent.com/en/product/software-informatics/mass-spectrometry-software (accessed 2025-12-10).
(2) *Mass Spec Fragment Prediction Software | MS Fragmenter*. ACD/Labs. https://www.acdlabs.com/products/spectrus-platform/ms-fragmenter/ (accessed 2023-10-19).
(3) *CASE NMR Software | Structure Elucidator Suite*. ACD/Labs. https://www.acdlabs.com/products/spectrus-platform/structure-elucidator-suite/ (accessed 2023-09-15).
